# Interpretable and accurate prediction models for metagenomics data

**DOI:** 10.1093/gigascience/giaa010

**Published:** 2020-03-09

**Authors:** Edi Prifti, Yann Chevaleyre, Blaise Hanczar, Eugeni Belda, Antoine Danchin, Karine Clément, Jean-Daniel Zucker

**Affiliations:** 1 IRD, Sorbonne University, UMMISCO, 32 Avenue Henri Varagnat, F-93143 Bondy, France; 2 Institute of Cardiometabolism and Nutrition, ICAN, Integromics, 91 Boulevard de l'Hopital, F-75013, Paris, France; 3 Paris-Dauphine University, PSL Research University, CNRS, UMR 7243, LAMSADE, place du Mal. de Lattre de Tassigny, F-75016, Paris, France; 4 IBISC, University Paris-Saclay, University Evry, Evry, 23 Boulevard de France, F-91034, France; 5 Institut Cochin INSERM U1016−CNRS UMR8104−Université Paris Descartes, 24 Rue du Faubourg Saint-Jacques, F-75014, Paris, France; 6 Sorbonne University, INSERM, Nutrition and Obesities; Systemic Approach Research Unit (NutriOmics), 91 Boulevard de l'Hopital, F-75013, Paris, France; 7 Assistance Publique-Hôpitaux de Paris, Nutrition Department, CRNH Ile de France, Pitié-Salpêtrière Hospital, 91 Boulevard de l'Hopital, F-75013, Paris, France

**Keywords:** prediction, interpretable models, metagenomics biomarkers, microbial ecosystems

## Abstract

**Background:**

Microbiome biomarker discovery for patient diagnosis, prognosis, and risk evaluation is attracting broad interest. Selected groups of microbial features provide signatures that characterize host disease states such as cancer or cardio-metabolic diseases. Yet, the current predictive models stemming from machine learning still behave as black boxes and seldom generalize well. Their interpretation is challenging for physicians and biologists, which makes them difficult to trust and use routinely in the physician–patient decision-making process. Novel methods that provide interpretability and biological insight are needed. Here, we introduce “predomics”, an original machine learning approach inspired by microbial ecosystem interactions that is tailored for metagenomics data. It discovers accurate predictive signatures and provides unprecedented interpretability. The decision provided by the predictive model is based on a simple, yet powerful score computed by adding, subtracting, or dividing cumulative abundance of microbiome measurements.

**Results:**

Tested on >100 datasets, we demonstrate that predomics models are simple and highly interpretable. Even with such simplicity, they are at least as accurate as state-of-the-art methods. The family of best models, discovered during the learning process, offers the ability to distil biological information and to decipher the predictability signatures of the studied condition. In a proof-of-concept experiment, we successfully predicted body corpulence and metabolic improvement after bariatric surgery using pre-surgery microbiome data.

**Conclusions:**

Predomics is a new algorithm that helps in providing reliable and trustworthy diagnostic decisions in the microbiome field. Predomics is in accord with societal and legal requirements that plead for an explainable artificial intelligence approach in the medical field.

## Background

An increasing wealth of data from high-throughput molecular and imaging technologies is connecting biomedical sciences and machine learning (ML). The latter is affecting numerous areas of medicine, including disease diagnosis and prognosis [1–3]. It is now argued that ML, and more globally artificial intelligence (AI), will dramatically improve prognosis within the coming years [4].

Simultaneously, progress made in high-throughput technologies has contributed to developing new fields such as metagenomics. The association of the gut microbiota with human health and disease has been widely discussed [5], and links with numerous diseases are described [6–13]. Specifically, ecological relationships among bacterial species such as mutualism, parasitism, and competition [14] may change along with a shift in microbial equilibrium. Although these signatures allow predicting disease onset and states, many of these findings are only correlative and require controlling for confounding factors—a task that remains challenging [15].

Metagenomics data must be interpreted carefully because they are often analyzed in a small number of samples (*N*) compared to a very large number of variables (*p*). Current microbial catalogues, which are composed of millions of genes [16] and thousands of bacterial species and functional profiles [17], allow the characterization and comparison of sampled ecosystems. Consequently, most models tend to overfit the training data and result in predictions arising from random sampling fluctuations [18, 19]. To reduce overfitting and allow for better generalization in unseen data, some authors use learning algorithms that include a dimension reduction or regularization methods, e.g., logistic regression with elastic-net regularization (ENET) [13] or Support Vector Machine (SVM) [15]. While these algorithms are more straightforward than others, they generate complex models that are difficult to interpret. ML research has focused on building accurate models for large data collections, often at the expense of interpretability.

Providing an explanation of the prediction process is increasingly requested [20] when not mandatory [21], especially in precision medicine [20, 21]. Interpretable models have 2 desirable properties: conciseness and readability by non-experts. They should contain simple operations and be limited in size [22–24].

Causality, as the holy grail of modern biology, is beyond the scope of the interpretability property of a predictive model. Here, we investigated whether models inspired by ecosystem relationships and sparse microbial signatures can be both accurate and more interpretable than more complex well-established state-of-the-art (SOTA) models, including logistic regression with ENET and SVM.

## Data Description

We used public datasets to test the "predomics" algorithm and compare it with SOTA methods. For the classification tasks we used curated metagenomic datasets from ExperimentHub [25] (see Supplementary Table S1 for more information). The code used to query and process the data is provided in the supporting dataset of this article doi: 10.55.24/100698. In total, 54 datasets were derived (i.e., 6 different cohorts and for each 6 taxonomic levels, a marker gene, and a pathway table along with a fused taxonomic dataset). Moreover, these datasets were transformed as "presence/absence" for additional experiments (*n* = 54). Baseline microbiome data were also used to predict the clinical outcome of bariatric surgery on morbidly obese patients [26]. Their microbiome was sequenced at baseline and at 1, 3, and 12 months after surgery (see original paper for methods).

## Analyses

### A new family of models for metagenomics data

**Figure 1:**
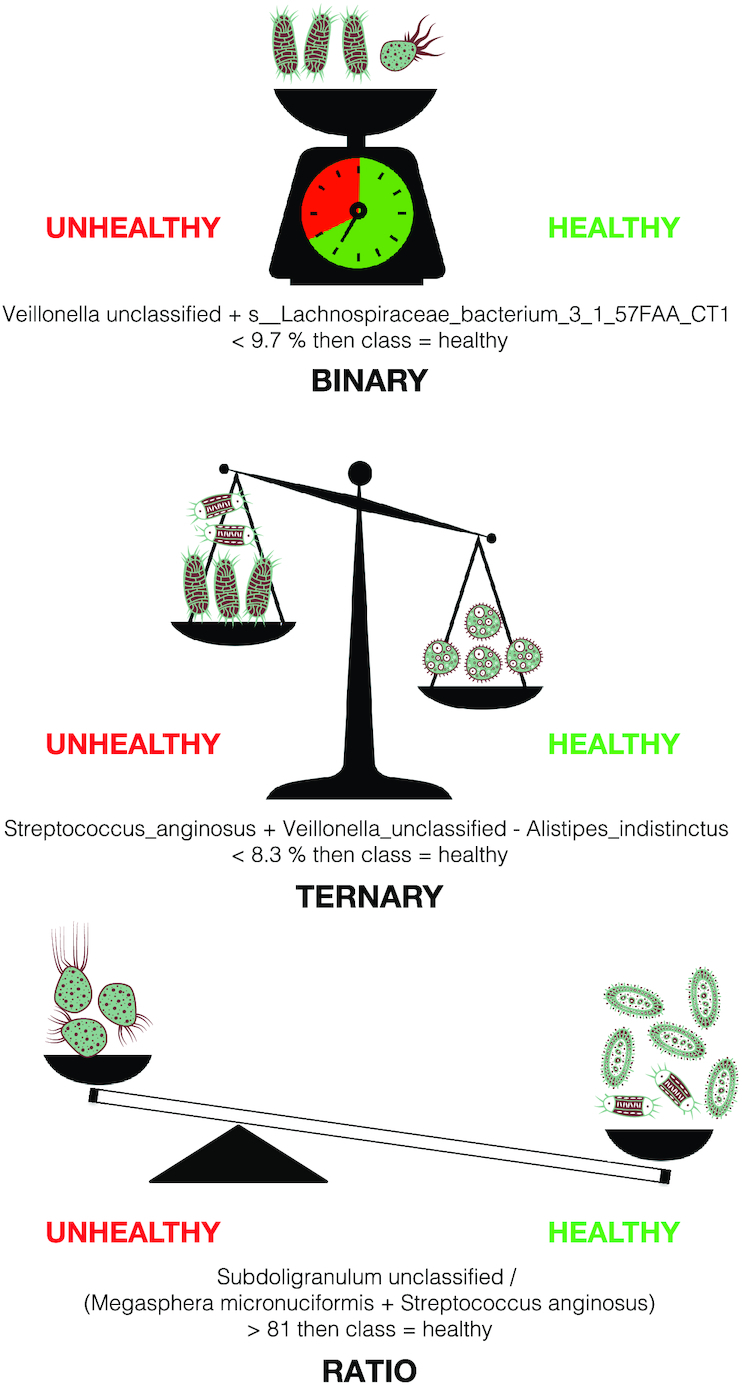
The 3 balance concepts depicting the BTR models. *Top*: The binary model tests whether the cumulated abundance of a set of species is below or above a certain threshold. *Middle*: The ternary model tests whether the cumulated abundance of a first set of species is below or above the cumulated abundance of a second set of species plus a certain threshold. *Bottom*: The ratio model tests whether the cumulated abundance of a first set of species over the cumulated abundance of a second set of species is above a given threshold.

We propose a new family of models, named BTR for binary/ternary/ratio, which are a simplification of linear models aiming at making their output even more interpretable. For each ecosystem ${y_{1\ }} \ldots \ {y_n}$, the abundance or presence of genes, taxonomy levels, functions, or other microbial qualities are represented by ${X_{1\ }} \ldots \ {X_p}$ predictor variables. In a linear model, a patient is predicted in a disease group with a probability of *p* > 0.5 if ${\beta _0} + \sum\nolimits_{j = 1}^p {\ {\beta _j}\ {X_j} > 0} $, where ${\beta _{0\ }} \ldots \ {\beta _p}\ \in R$ are real coefficients. The biological assumption is that the contribution of each bacterial species to the prediction is proportional to its abundance and that only a limited number of species is sufficient to support the prediction. BTR models are much simpler and are inspired by 3 hypotheses emphasizing relationships between species and associated ecosystem (Fig. 1).

Hypothesis 1: The unweighted cumulative abundance of a group of species can predict disease states. We define "binary models" (Bin) as linear models with the additional constraint that each coefficient ${\beta _1} \ldots {\beta _p}$ (omitting the intercept ${\beta _0}$) must be binary—{0, 1} (Fig. 1 top). An example is in (1) and is interpreted as “if the cumulative abundance of *s_Veillonella_unclassified* and *s_Lachnospiraceae_bacterium_3_1_57FAA_CT1* is <9.7% of the total microbial abundance, then the individual is classified as healthy.” These species may share the same ecological niche or interact directly with one another [27, 28].

(2) ***I**f s_Veillonella_unclassified + s_Lachnospiraceae_bacterium_3_1_ 57FAA_CT1 **<** 0.097 then class **=** healthy*

Hypothesis 2: The difference of unweighted cumulative abundance of 2 groups of species can predict disease state. This assumption is implemented by "ternary models" (Ter), also linear models with the constraint that each coefficient ${\beta _1} \ldots {\beta _p}$ (omitting the intercept ${\beta _0}$) be limited to the values {−1, 0, 1} (Fig. 1 middle). An example of a ternary model in (2) can be interpreted as follows: “if the cumulative abundance of *s_Streptococcus_anginosus* and *s_Veillonella_unclassified* minus the abundance of *s_Alistipes_indistinctus* is ≥8.3% of the total microbial abundance, then the patient is classified as healthy.”

(2) *If(s_Streptococcus_anginosus + s_Veillonella_unclassified)/s_Alistipes_indistinctus ≤ 0.083thenclass****=****healthy*

Hypothesis 3: The ratio of unweighted cumulative abundance of 2 groups of species can predict disease state. This assumption is implemented by "ratio models" (Ratio), also linear models with an additional constraint: each coefficient ${\beta _1} \ldots {\beta _p}$ is limited to a value of −$\theta $, 0, or 1, where $\theta $ is a positive real number, and the intercept ${\beta _0}$ is set to zero (Fig. 1 bottom). An example in (3) can be interpreted as follows: “if the abundance of *s__Subdoligranulum_unclassified* is $\theta \ = \ 81$ times greater than the cumulative abundance of *s__Megasphaera_micronuciformis*+*s__Streptococcus_anginosus* then the individual is classified as healthy.”

(3) Ifs__Subdoligranulum_unclassified > 81 * (s__Megasphaera_ micronuciformis + s__Streptococcus_anginosus)thenclass***=****healthy*

Biologically, both Ter and Ratio models can correspond to interactions of different types of species, including cooperation and competition among species. BTR models can be illustrated as balances, where species abundance is symbolized by the cumulative weights (Fig. 1). The concept of balance is not new in ecology and was first proposed to address the compositionality problem in microbiome data. A balance-based representation can bypass this issue and reveal pertinent biological patterns [29]. Recently, other authors have applied the balance representation in the classification context [30]. Here, we propose a more general framework of models that encompass such balances. Indeed, they would correspond to our Ter models when applied to log-transformed data—named TerLog (see Supplementary Fig. S14). Learning linear models on log-transformed relative abundance data corresponds to identifying balances of multiplicative relationships. However, which characterizes best microbial ecosystems (i.e., multiplicative or additive) remains an open question. We propose here different types of models that could be useful in tackling such questions. The predomics algorithm was developed to specifically learn BTR models.

### BTR models are sparse and accurate and improve with taxonomic specificity

**Figure 2:**
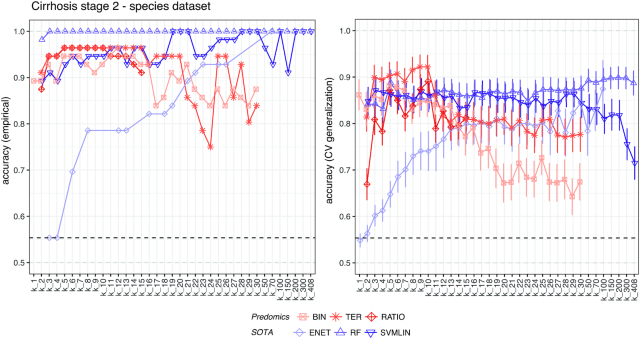
Model performance across different model size. *Left:*Training accuracy of the best models (on the y-axis) in the Cirrhosis Stage-2 dataset for different model size *k* (indicated *k_*# on the x-axis). *Right:*Testing accuracy of the best models for each model size as the average of 10-times, 10-fold cross-validation (CV) sets ± standard error of the mean. Dashed line indicates the majority class (i.e., the accuracy obtained when simply predicting the majority class through chance alone).

We tested our approach on 6 different public metagenomic datasets (Table S1) and 9 derived types of variables (6 different taxonomic levels, a merged multi-taxonomic level, marker genes, and a functional MetaCyc pathway table, i.e., a total of 54 datasets; see Methods). We trained and tested models with different numbers of features (i.e., model size, k_#) and noticed an effect on accuracy. As expected, the testing performance on unseen data was lower compared to training performance. However, this difference was more pronounced for the SOTA, indicating a significant overfitting effect, compared with BTR models as discussed previously [19]. The simplicity and sparsity of the BTR models reduces overfitting on studied datasets (Fig. 2). Because BTR models come with an embedded feature selection strategy, we used a Mann-Whitney test to select the k_# most correlated features for random forest (RF) and SVM to allow comparison. For ENET we used the embedded regularization path and selected the first k_# from it.

**Figure 3:**
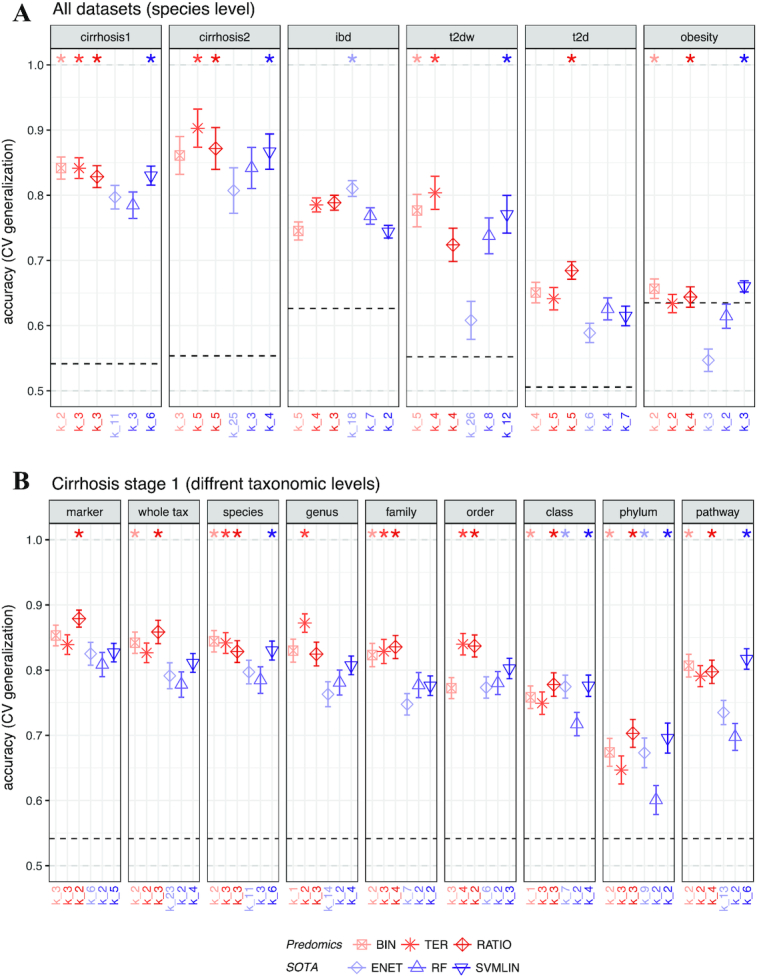
BTR (i.e. Bin/Ter/Ratio) models vs SOTA (i.e. State-of-the-art: ENET= ElasticNet; RF=Random Forrests; SVMLIN=Support Vector Machines with linear kernel) models performance across different diseases and taxonomic levels. **A**: Accuracy measured in the test datasets at the species level across 6 different datasets. The stars on top indicate whether the corresponding BTR or SOTA algorithms are significantly better than others (i.e., without stars). **B**: Accuracy measured in the test datasets in different taxonomic levels of gut microbiome quantification (species, genus, family, order, class, and phylum, whole taxonomy) as well as in marker gene and pathway abundance tables. Dashed bars indicate the majority class and *k_*# indicates the model-size. The 10-times 10-fold validation test values are summarized as mean ± standard errors.

We applied a model size penalization technique on the empirical (training) accuracy to select the best model. BTR models performed at least as well as the SOTA in 46 of 54 (85%) of the cases. They outperformed SOTA in 19 of 54 (35%) and were outperformed in 8 of 54 (15%) (Fig. 3; Fig. S1A–C). Similar results were observed even when all the variables in the dataset were used (no penalization) for the SOTA (Fig. S2A–C) or when fixing the same model size for all the compared models (Fig. S3A–C). Similar results are obtained for additional performance scores including recall, precision, and f1 score (Figs S6 and S7).

When learning from the different types of variables based on taxonomic levels (Cirrhosis Stage 1), the performance of the models varies accordingly. Higher performance is obtained at the gene marker, species, and genus levels and decreases with higher taxonomic levels. Similar results are reported elsewhere [31]. Moreover, when applied to a multi-taxonomic level dataset (from strain to phylum as generated by Pasolli et al. [25] with different specificity levels mixed together; i.e., "whole tax"), models displayed surprisingly good performance (Fig. 3B). Indeed, in this space, models can be powerful because they can summarize more complex rules such as: “if (abundance of all Firmicutes − abundance of all Clostridiales order) **>** threshold then disease*.”*

We tested the generalization of Bin, Ter, Ratio and also TerLog models trained in Cirrhosis Stage 1, in a second, independent dataset (i.e., Cirrhosis Stage 2). Results illustrated in Fig. S5 indicate very good external validation with an average training accuracy = 0.89 (SD = 0.02) and testing accuracy = 0.85 (SD = 0.04). Ter and Ratio models generalized better compared to Bin and TerLog.

In addition to the abundance datasets described above, we trained and tested similar models on presence/absence binary data derived from the previous 54 abundance datasets. Overall results are similar, indicating that the detection of species alone can be powerful enough in some prediction tasks (see Supplementary Figs S1D–F, S2D–F, S3D–F, and S4). It is noteworthy that, when applied to presence data, BTR models indicate relationships between sub-ecosystem complexity or richness. These can be useful to detect switch-like mechanisms in the microbiome.

### BTR models generate straightforward interpretations in contrast to state-of-the-art models

**Figure 4:**
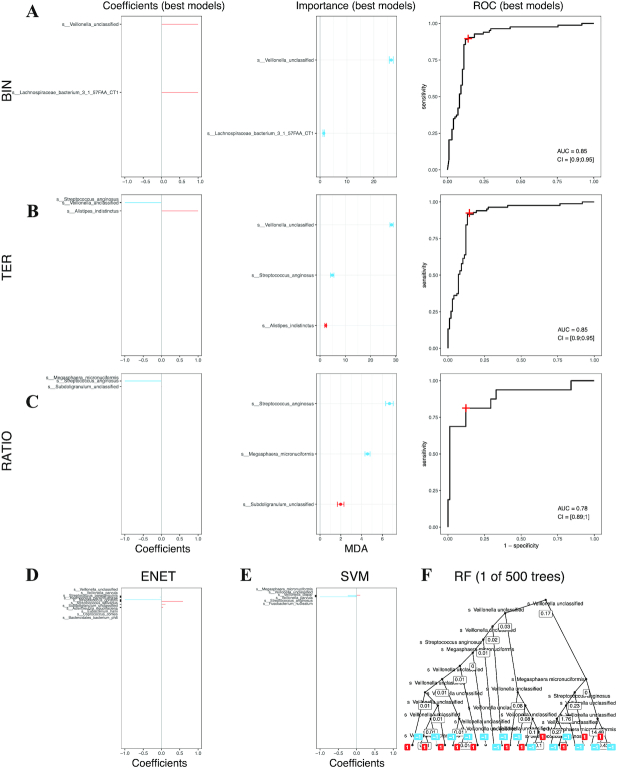
BTR models are interpretable compared to state of the art. **A–C***left*: Barcode graphical representations indicating the coefficients (1 or −1) of the BTR model features sorted by decreased correlation strength with the class to predict. **A–C***middle*: Mean decrease accuracy (MDA) plots indicating feature importance computed during the cross-validation process. Blue and red colours indicate enrichment in patients and controls, respectively. **A–C***right*: Receiver operator characteristic (ROC) plots for the same BTR models. The red cross indicates the specificity and sensitivity of the model. **D–F**: A visualisation attempt of the SOTA models with barcode plots indicating the coefficients (values in [−1, 1]) for ENET and SVMLIN, and only 1 tree out of the 500 used in the RF model. AUC: area under the curve; CI: confidence interval.

A graphical barcode representation illustrates the simplicity of BTR models. In Fig. 4A–C*left*, the models are represented by red and blue horizontal lines, corresponding, respectively, to positive and negative coefficients (either 1 or −1). The same representation is used to visualize the normalized coefficients of ENET and SVMLIN models (the line length is proportional to the coefficient in the interval [−1, 1]) (Fig. 4D–E). For the RF model only 1 of the 500 decision trees used in the model is illustrated (Fig. 4F). Additionally, for each variable selected by BTR models, we assessed their importance in prediction, using a variant of the well-known mean decrease accuracy (MDA) (Fig. 4A–C, *middle*). The feature importance (FI) score of BTR models correlates strongly with the FI of the well-established but more complex RF model (respectively, *R* = 0.68, *R* = 0.81, *R* = 0.7, with Bin, Ter, and Ratio models; Figs S11 and S12). This information allows prioritizing further exploration of the features in the context of the predicted phenomenon.

**Figure 5:**
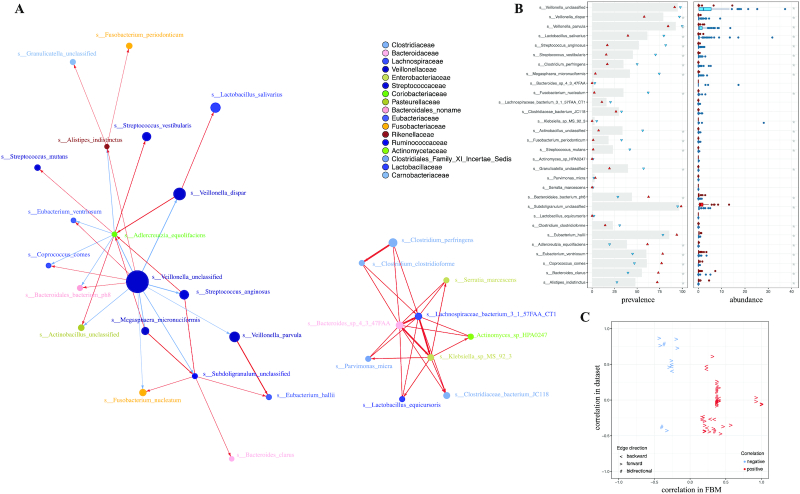
Feature co-occurrence network in the family of best models. **A**: This network displays feature co-occurrence patterns in FBM models. Only the top 5% strongest edges inferred using the ScaleNet network reconstruction approach (parameterized with bayes_hc and aracne algorithms; see Methods) are shown. The size of the nodes is proportional to the average importance (MDA) in the Bin, Ter, and Ratio experiments. The colours of the nodes indicate the taxonomic family assignation as indicated in the legend. The red and blue edges indicate co-presence and co-absence in the models, respectively. **B**: For each feature present in the network we show (*left*) the prevalence of the features in the whole dataset (grey bar) and in the prediction classes (disease, healthy) depicted as blue and red dots, respectively, and (*right*) the feature abundance distribution in the prediction classes (disease, healthy) depicted as blue and red box plots, respectively. Grey stars indicate significant differences. **C**: A scatter-plot indicating for each edge of the network the correlation between the 2 features in the data and FBM, respectively, in the y- and x-axis. The colour is the same as for the edges in the network, while the shape indicates the direction of the edges in the network.

Predomics generates a family of BTR models with equivalent predictive power in a given model size range (i.e., family of best models [FBM]; Fig. S8; see methods and supplementary material). The FBM is analyzed to identify the common features that are found in the models. For instance, in the Cirrhosis Stage 1 (species) dataset, the 268 models in the FBM with model size < 6 rely on only 67 features (i.e., 16% of the whole dataset), which can be used to infer a "feature co-presence network in models" (Fig. 5A). An emerging property of this network is the clustering of phylogenetically related species, such as Firmicutes enriched in patients (blue tones) and Proteobacteria and Actinobacteria enriched in controls (green tones). Co-presence of the features indicate complementarity in prediction (red edges), while replacement of the features by one another indicate redundancy (blue edges). This can also be observed with the inverse relation of feature pairs in the data and in the models (Fig. 5C)—the most correlated pairs in the dataset are those that do not occur together in the models. This network provides precious information to decipher the sub-ecosystem that is associated with the disease (Figs S8–S10).

### BTR models provide biological insights

We focused on the liver cirrhosis dataset [11], where major patient dysbiosis was observed with decreased microbial richness, depletion of gut commensals, and an invasion of oral bacteria. Several markers at taxonomic and functional levels were associated with the disease.

**Figure 6:**
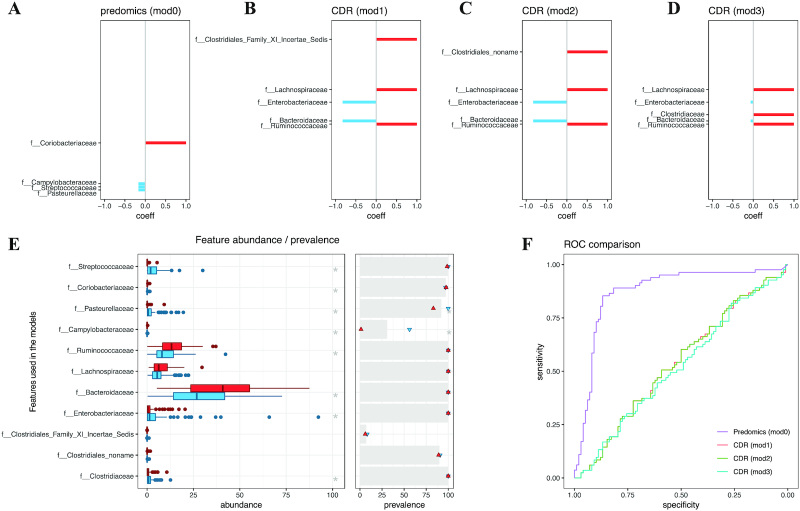
Cirrhosis Dysbiosis Ratio (CDR) index compared to predomics ratio model. **A–D**: Barcode plots indicating the coefficients of the Ratio models (S13–S15) built with features from the CDR index and predomics discovered model (S16). Red and blue colours indicate, respectively, the numerator and denominator of the ratio model and are, respectively, enriched in the controls and patients with liver cirrhosis. The length of the lines is proportional to the ratio factor optimized in the model. **E***left*: Box plots indicating the abundance distribution by class for all features used in these models (red is enriched in controls and blue in the liver cirrhosis group). *Right*: For the same features the prevalences of non-zero values are depicted in grey for the whole cohort and red and blue dots, respectively, in the control and patient groups. Grey stars indicate significant difference. **F**: Receiver operating characteristic (ROC) curves for the 4 models (S13–S16).

Some authors have modelled liver cirrhosis–associated microbiome features using curated information from the literature, such as the ratio of autochthonous (butyrate-producer bacteria) to non-autochthonous (oral bacteria, opportunistic pathogens). They used these taxa to build a cirrhosis dysbiosis ratio (CDR) score [32]. On the basis of their description we built 3 redundant ratio models using family taxonomic features to reproduce their score and applied them in the Liver Cirrhosis Stage 1 (family) dataset [11] (Fig. 6B–D). We searched the same family-level dataset for Ratio models. The models that were identified provided superior performance (accuracy = 0.86; Fig. 6A) compared with CDR-based models (i.e. mod1, mod2, mod3; accuracy = 0.56 in average; Fig.   6F). The reason for the CDR lower performance can be explained by the inclusion of the Bacteroidaceae family in the liver cirrhosis group by the authors, while we observe the opposite association in the current dataset. Bacteroidetes-related features are enriched in the control group, and this is consistent for different taxonomic levels (Fig. 6E, see supplementary material).

At the phylum level, the Ratio model (S6) points at a mutual exclusion between Bacteroidetes and the combination of Proteobacteria and Viruses, which is also picked up by the Bin model (S4). These models are in line with a decrease in Bacteroides and an increase in Proteobacteria and Fusobacteria in the liver cirrhosis group, reported in the original study. The decrease in Bacteroidetes indicates a decrease in highly prevalent gut bacteria, whereas the increase of Proteobacteria has been repeatedly reported in dysbiotic microbiomes of patients and has been associated with chronic inflammation and serum lipopolysaccharides [33, 34]. The Virus prevalence in the liver cirrhosis group may reflect the oral microbiome signature or increased incidence of viral infections together with opportunistic pathogens.

The potential competition between oral and gut microbes reported in previous studies [35] is best reflected by Ter and Ratio models with genus abundance data, which combine *Veillonella* (oral bacteria; opportunistic pathogen) enriched in liver cirrhosis at 1 side and *Bacteroides* plus *Eubacterium*(S9) or *Coprococcus*(S8) enriched in controls. The latter represent butyrate producers (*Coprococcus* and *Eubacterium*) and complex polysaccharide degraders (*Bacteroides* genus) [36]. Among the most important genera in the FBM we find *Veillonella*, *Streptococcus*, *Haemophilus*, *Coprococcus*, and *Lactobacillus*, all more abundant/prevalent in patients.

The best Ratio and Ter models (1–3) include oral bacterial species of the genus *Veillonella*(*Veillonella*unclassified)*, Streptococcus*(*S. parasanguinis* and *S. anginosus*), and opportunistic pathogens like *Megasphaera micronuciformis* that proliferate in patients with liver cirrhosis, whereas butyrate producers of the genus *Subdoligranilum* (*Subdoligranilumunclassified)* closely related to *Faecalibacterium prausnitzii* [37] and complex polysaccharide-degrading species like *Bacteroides cellulosilyticus* [38] characterize control subjects. *M. micronuciformis* was previously associated with primary biliary cirrhosis on the basis of 16S ribosomal RNA quantification [39]. A more in-depth exploration of the FBM (Figs S8–S10) and the feature-model co-occurrence network (Fig. 5) delineates detailed relations of the predictive sub-ecosystem.

At the functional level, predictive models (S10–S12) from MetaCyc pathway abundance data include pathways that suggest an increased aerobic metabolism (HEMESYN2−PWY: heme biosynthesis II [anaerobic], essential for cytochromes and heme-containing globins; PWY−922: mevalonate pathway I, needed for the biosynthesis of ubiquinone and menaquinone complexes of respiratory chains). Interestingly, increase in aerobic respiration profiles has also been identified as a metabolic signature of inflammation-associated dysbiosis in models of colitis [40]. Moreover, we observe the presence of modules related to bacterial peptidoglycan biosynthesis in the FBM (PWY−6470: peptidoglycan biosynthesis V). It has been described as an elicitor of inflammatory response associated with the progression of liver cirrhosis [41], in agreement with a more inflammatory profile of cirrhotic patients.

Altogether, these results indicate that BTR models discover important features with relevant biological information. BTR models are more accurate than literature-based ones and have the ability to distil and capture the predictive biological information embedded in the data.

## Discussion

Here, we introduced “predomics,” an original ML approach, inspired by microbial ecosystem interactions. We have demonstrated that it discovers not only accurate predictive signatures that generalize well, but also provides unprecedented interpretability. Moreover, the FBM offers the ability to distil biological information and to decipher the predictability signatures of the studied condition.

In principle, BTR models could be applied to any type of data. However, they are best suited to commensurable measurements (i.e., variables measurable by the same standard or measure). In the growing field of metagenomics, issues related to compositionality and data processing still remain to be solved. An advantage of the Ratio models that we proposed is that they are scale-invariant given they do not depend on absolute measurements, thus avoiding compositionality issues.

Other issues related to data quantification can affect predictive models. Recent work has shown the importance of data acquisition in subsequent analytical inferences. In particular, microbial loads differ significantly between individuals and are associated with specific types of microbial ecosystems [42]. Moreover, varying sequencing depth can over- or under-estimate less abundant taxa that may be selected by the models. It is thus advisable to pre-filter rare taxa from the dataset before training the models. However, the sparsity constraint in our approach will force important taxa to be selected by the models, improving their generalization.

The simplicity of BTR models may come with the risk of over-interpretation. The existence of *k* species in a model may correspond to different explanations ranging from simple correlation to causal relation. They may or may not interact, as in the case of a niche differentiation [30]. For instance, the oral-originated species found in the gut of patients with liver cirrhosis [11] along with the absence of usual commensals may reflect a global difference in the environment where they live rather than direct interaction [11]. Explicit identification of co-varying species provides important knowledge that can be used to propose functional interactions between species or between the species of interest and the host. Yet, even if BTR models represent real interactions between species, it is not recommended to propose a causal interpretation without further experimental validation.

We also stress that the quality of reference datasets used in establishing predictive models is crucial for model interpretability. The propagation of errors and inaccuracies in genomic datasets is a well-known issue that negatively affects the outcome of automated methods used for functional annotation [43]. Moreover, orphan enzymatic activities, by definition, cannot be associated with gene sequences while the number of sequences with unknown functions is extremely large and keeps increasing, making error percolation a widespread feature [44] (see supplementary material).

Another important issue that plagues microbiome studies is the influence of potential confounders modulating microbial ecosystems. For instance, it has been shown that metformin can alter the bacterial ecosystem in such a way that some bacterial species (e.g., *Escherichia coli*) are increased in abundance while others are depleted [15]. In this context, we advise users to filter out confounder-related species from the data or to discard models that are sensitive to confounders.

Finally, besides quantifying taxa abundance through whole shotgun or 16S ribosomal RNA sequencing, BTR models can be used to develop specific acquisition technologies such as microarray DNA chips or qPCR-based tests, built with primers that are specific to the species/taxa found in the models [45]. From a clinical perspective, because BTR models rely on a small number of variables, quantifying a relatively small subset of variables (genes, species, pathways, operational taxonomic units, etc.) can be sufficient to simultaneously predict multiple tasks, and this can be developed for a limited cost. Such applications, after being properly validated, will be important to the medical community in their translational quest in improving patient care. Our approach brings us a step closer towards useful clinical predictions while preserving interpretability.

### Potential implications

In our article, we propose an original ML method, called predomics, which is tailored for metagenomics data. Because it is generic, it can be applied to other fields as well. We strongly believe that this original approach will have significant impact on both the development of predictive models based on metagenomics data as well as their applications to medical conditions. This approach will support clinical decisions in the context of precision medicine. The interpretability of the models will ease acceptability and suggest candidates for microbiome targeted treatments. Moreover, it will serve as a bridge to further develop cross-fertilization between AI, biology, and precision medicine.

## Methods

### The predomics optimization algorithm

Here, we propose a new family of models, named "BTR" for Binary/Ternary/Ratio. Learning optimal BTR models is computationally difficult. Because weights are discrete, usual techniques coming from convex optimization do not apply. A naive way would be to perform an exhaustive search through the whole space of models. Unfortunately, this is not practically feasible because the computation time would increase exponentially with the number of features. The BTR learning problem is known as NP-Hard, which means that no algorithm can solve this problem exactly in polynomial time [46].

We can nevertheless apply heuristics that provide good models without guarantee on their optimality. Genetic algorithm is a stochastic optimization technique that can be of great use in such context. It adopts concepts from evolutionary biology—populations, reproduction, mutation, and generations. The outline of the algorithm is described in the supplementary materials section. After the evolution process, a final population of predictive models is provided. The best model is obtained by applying a so-called "model size penalization" (accuracy_penalized_ = accuracy − ƛ *k*), where *k* is the number of features in the model (i.e., parsimony) and ƛ is a hyperparameter controlling the penalization of the accuracy. Here, we used ƛ = 1%, which means that a model that is using 1 additional feature will only be preferred if it improves the accuracy by >1%.

For classification, predomics may be set to optimize different parameters such as the accuracy (default), AUC, F1, precision, or recall, while for regression it can optimize *R*^2^ (default), Spearman ρ, or the standard error of the regression.

### Experimental design

The experimental pipeline proceeds as follows:

1. Feature normalization: frequency tables are used as processed by Pasolli et al [25].
2. Features with low standard deviation are filtered out. The threshold corresponds to the maximum second derivative of the distribution of the feature's standard deviation.
3. The generalized performance of each method is estimated by 10-times 10-fold cross-validation for the classification tasks and a 20-times 5-fold cross-validation for the regression tasks.
4. The feature selection is embedded for the BTR models and Elastic Net (ENET). For SVM and RF, feature selection is based on the Mann-Whitney score as introduced in Aron-Wisnewsky et al. [26].
5. Algorithm performances are compared with a paired *t*-test using the 100 CV estimations. Those that are not significantly different (*P* < 0.05) are considered equivalent.

The BTR models are tested on 109 different datasets (see Table S1) and compared with the methods from the SOTA algorithms: SVM with linear and Gaussian kernel (data not shown), random forest, and ENET (an improvement of Lasso, α = 0.5). All algorithms were evaluated by measuring test accuracy in a cross-validation setting and compared among them using paired *t*-tests. A specific comparison between TerLog models (i.e., Ter model with log-transformed data) and the geometric mean balance algorithm is provided in the supplementary material.

### Family of best models

An FBM is defined as the set of models returned by the algorithm, whose accuracy is within a statistically equivalent window, defined by a threshold assuming a binomial distribution (*P* < 0.05). An FBM can be analyzed in detail to distil biological information in the predictive context (see supplementary material).

### Feature importance

Similar to RF, "feature importance" is defined as the usefulness of features to be predictive, given all other features and best models of the FBM. During each cross-validation fold, the out-of-bag error on each model of the FBM is computed. The importance of the ${j^{th}}$ feature is measured by permuting all features within the out-of-bag data. The out-of-bag error is computed on these perturbed data for each FBM model. The overall feature importance for the ${j^{th}}$ feature is obtained by averaging over all FBM models the difference in out-of-bag error before and after the permutation. This is performed on all the features of the dataset that are found in the FBM models, on which errors before and after permutations are computed. Finally, the MDA is computed as the average of these values over all the folds and is displayed along with the standard error of the mean.

### Threshold optimization

The threshold used in the model is optimized to maximize the performance in the training set. This approach consists in computing the model's score for each observation in the training set. The observations are ordered on the basis of this score. Next, the cumulative error is computed following the same order—each time an example is misclassified, the cumulative error is increased when sliding through the score. The index example on which the cumulative error minimizes will provide the value of the score, which will be used as a threshold in the final model.

### Regression models

Predomics can learn regression models, which are evaluated by maximizing either Spearman ρ or Spearman *R*^2^ or minimizing the scaled standard error of regression. The model's score at this stage reflects the cumulative/difference/ratio of relative abundance of the species and needs to be scaled in the range of the variable to predict. Two additional parameters α (i.e., multiplication factor) and β (i.e., intercept) are estimated.

### Network reconstruction

We used Scalenet [47] to reconstruct the feature co-presence network in model selection data. Here we used the top 5% strongest edges inferred by bayes_hc and aracne methods in the FBM-presence table. ScaleNet first reduces the reconstruction problem to a number of simpler reconstruction problems, then uses SOTA reconstruction methods to solve them. Finally, a consensual voting strategy between the methods is adopted to identify accurate subgraphs, which are then overlapped together.

## Availability of Source Code and Requirements

Supplementary information and source data files are available online and the predomics package in https://github.com/eprifti/predomics. The software is registered in the scicrunch.org repository under the RRID:SCR_017415.

- Project name: Predomics
- Project home page: e.g., https://github.com/eprifti/predomics
- Operating system(s): Platform independent
- Programming language: R
- Other requirements: R (version ≥2.15.0). Predomics imports the following R packages: reshape2, plyr, BioQC, foreach, snow, doRNG, yaml, ggplot2, gridExtra, grid, gtools, RColorBrewer, glmnet, pROC, viridis, kernlab, randomForest.
- License: GNU General Public License v3.0
- RRID:SCR_017415

## Availability of Supporting Data and Materials

The supplementary material includes additional experiments and results. The datasets used here are public in respective repositories. For the classification tasks we downloaded 5 curated metagenomic datasets from the ExperimentHub [25]. The raw data were generated in independent studies using shotgun metagenomics (Table S1) and were processed bioinformatically and curated by Pasolli et al. [25]. For the regression experiments, we used shotgun metagenomics data from a recently published study, where morbidly obese patients underwent bariatric surgery [26]. The code for processing the data is made available in the project's home repository. Snapshots of our code and other data further supporting this work are openly available in the *GigaScience* repository, GigaDB [48].

## Additional Files

**Table S1:** Summary of the datasets considered in the experiments.

**Figure S1:** Best model performance across all experiments (penalization strategy).

**Figure S2:** Best model performance across all experiments (no-penalization for SOTA).

**Figure S3:** Best model performance across all experiments (fixed *k* = 5 for BTR and SOTA).

**Figure S4:** BTR and SOTA performance across different disease and taxonomic levels in presence/absence data.

**Figure S5:** Validation of BTR models in an external dataset.

**Figure S6:** Best model performance across all experiments (penalization strategy) different measurements.

**Figure S7:** BTR and SOTA performance across different taxonomic levels in Cirrhosis Stage 1 species normalized abundance data.

**Figure S8:** Performance of the family of best models (FBM) across model size and model type.

**Figure S9:** Feature composition and feature importance of the family of best models for the Cirrhosis Stage 1 dataset.

**Figure S10:** Feature abundance and prevalence of the family of best models for the Cirrhosis Stage 1 dataset.

**Figure S11:** Comparison of feature importance between BTR models and RF as well as statistical ranking.

**Figure S12:** Comparison of feature importance between TER models and RF.

**Figure S13:** Quantitative prediction of phenotypic outcome after bypass surgery.

**Figure S14:** Comparison of selbal balances with TerLog predomics models.

## Abbreviations

AI: artificial intelligence; AUC: area under the curve; BTR: Bin, Ter, Ratio models; CDR: cirrhosis dysbiosis ratio; ENET: elastic net; FBM: family of best models; FI: feature importance; MDA: mean decrease accuracy, indicating the importance of features in prediction; ML: machine learning; RF: random forest; SD: standard deviation; SER: standard error of regression; SOTA: state of the art; SVM: Support Vector Machine; SVMLIN: SVM with linear kernel; TerLog: Ter models when applied to log-transformed data.

## Competing Interests

The authors declare that they have no competing interests.

## Funding

This work was supported by the French National Agency through the national program Investissements d'Avenir (reference no. ANR-10-IAHU-05) IHU ICAN; by the Funding Support of European Union's Seventh Framework Program under grant agreement HEALTH-F4-2012-305312, by the Assistance Publique-Hôpitaux de Paris promoter of the clinic program, and by Assistance Publique-Hôpitaux de Paris Contrat d'interface chercheurs 2015–2018.

## Authors’ Contributions

E.P.: overall conception, design and interpretation; designing and coding the software; conducting all experiments; writing the manuscript. Y.C. conception and interpretation of the approach; coding; drafting the manuscript. B.H.: conception, design and interpretation of the approach; coding; drafting the manuscript. E.B.: biological interpretation of the results; drafting the manuscript. A.D.: biological interpretation of the results; drafting the manuscript. K.C.: data production (bariatric model); biological interpretation of results. J.D.Z.: conception, early prototyping, design and interpretation of results; drafting and writing the manuscript. All authors approved the manuscript.

## Supplementary Material

giaa010_GIGA-D-19-00177_Original_Submission

giaa010_GIGA-D-19-00177_Revision_1

giaa010_GIGA-D-19-00177_Revision_2

giaa010_GIGA-D-19-00177_Revision_3

giaa010_Response_to_Reviewer_Comments_Original_Submission

giaa010_Response_to_Reviewer_Comments_Revision_1

giaa010_Response_to_Reviewer_Comments_Revision_2

giaa010_Reviewer_1_Report_Original_Submission Kang Ning -- 6/27/2019 Reviewed

giaa010_Reviewer_1_Report_Revision_1 Kang Ning -- 9/16/2019 Reviewed

giaa010_Reviewer_1_Report_Revision_2 Kang Ning -- 12/15/2019 Reviewed

giaa010_Reviewer_2_Report_Original_Submission Bonnie Hurwitz -- 8/2/2019 Reviewed

giaa010_Reviewer_2_Report_Revision_1 Bonnie Hurwitz -- 10/1/2019 Reviewed

giaa010_Supplemental_Files
