# Supplementary material for: Interpretable and accurate prediction models for metagenomics data
Source: Gigascience. 2020 Mar 9;9(3):giaa010. doi: 10.1093/gigascience/giaa010 (PMC7062144; doi:10.1093/gigascience/giaa010)
Supplement: giaa010_GIGA-D-19-00177_Revision_1 [file giaa010_giga-d-19-00177_revision_1.pdf]

GigaScience

Interpretable and accurate prediction models for metagenomics data

--Manuscript Draft--

**Manuscript Number:**	GIGA-D-19-00177R1
**Full Title:**	Interpretable and accurate prediction models for metagenomics data
**Article Type:**	Research
**Funding Information:**	Assistance Publique-Hôpitaux de Paris (Contrat d'interface chercheurs 2015-2018) — Professor Jean-Daniel ZUCKER<br>French National Agency through the national program Investissements d'Avenir IHU ICAN (ANR-10-IAHU-05) — Dr Edi Prifti<br>Funding Support of European Union's Seventh Framework Program (HEALTH-F4-2012-305312) — Prof. Karine Clément
**Abstract:**	Background: Microbiome biomarker discovery for patient diagnosis, prognosis and risk evaluation is attracting broad interest. Selected groups of microbial features provide signatures that characterize host disease states such as cancer or cardio-metabolic diseases. Yet, the current predictive models stemming from machine learning still behave as black boxes and seldom generalize well. Their interpretation is challenging for MDs and biologists, which makes them difficult to trust and use routinely in the physician–patient decision-making process. Novel methods that provide interpretability and biological insight are needed. Here, we introduce "predomics", an original machine learning approach, which is tailored for metagenomics data. It discovers not only accurate predictive signatures, but also provide unprecedented interpretability. Inspired by microbial ecosystem interactions, the decision provided by the predictive model is based on a simple, yet powerful score computed by adding, subtracting or dividing cumulative abundance of microbiome measurements. Results: Tested on more than 100 datasets, we demonstrate that predomics models are extremely simple and thus highly interpretable. Even with such simplicity, they are as accurate as state-of-the-art methods. Moreover, the family of best models, discovered during the learning process, has the ability to distill biological information and help decipher the predictability signatures of the studied condition. Finally, in a proof-of-concept experiment, we successfully predicted body corpulence and metabolic improvement after surgery using pre-bariatric surgery microbiome data. Conclusions: Collectively, this approach builds up both reliable and trustworthy diagnostic decisions while agreeing with societal and legal pressure that require explainable AI in the medical field.
**Corresponding Author:**	Jean-Daniel ZUCKER<br>Institut de recherche pour le développement France-Nord<br>BONDY CEDEX, Île-de-France FRANCE
**Corresponding Author Secondary Information:**	
**Corresponding Author's Institution:**	Institut de recherche pour le développement France-Nord
**Corresponding Author's Secondary Institution:**	
**First Author:**	Edi Prifti, PhD
**First Author Secondary Information:**	
**Order of Authors:**	Edi Prifti, PhD
	Yann Chevaleyre, PhD
	Blaise Hanczar, PhD
	Eugeni Belda, PhD
	Antoine Danchin, PhD

Powered by Editorial Manager® and ProduXion Manager® from Aries Systems Corporation

	Karine Clément, PhD
	Jean-Daniel ZUCKER
**Order of Authors Secondary Information:**	
**Response to Reviewers:**	Dear Dr. Nogoy,<br><br>Please accept our gratitude in allowing our work to be reviewed in Gigascience and accompanying us during this process.<br><br>We have carefully examined both reviews and have tried our best to answer them point-by-point and in a constructive manner. We have performed additional analyses to answer some of the questions raised by the reviewers and improved upon their advice both the revised version of the manuscript as well as the supplementary material.<br><br>Moreover, we registered the predomics software in the scicrunch.org repository as requested under the accession number RRID: SCR_017415 and mentioned it in the revised version of the manuscript (page 14).<br><br>We hope that both the reviewers and the editorial board will find our answers and the new version of the manuscript satisfying.<br><br>On a more general note, we would like to stress that we particularly appreciate the principles to which the journal Gigascience adheres and that they guided us in the choice to submit a manuscript.<br><br>With our best regards<br>Edi Prifti and Jean-Daniel Zucker<br><br><br>PS: As the detailed responses to the reviewers including additional figures and analyses are provided as a supplementary document uploaded with the revised manuscrit.<br>The file is called "Predomics_Cover_letter_And_Response_To_Reviewers.docx"
**Additional Information:**	
**Question**	**Response**
Are you submitting this manuscript to a special series or article collection?	No
**Experimental design and statistics**<br><br>Full details of the experimental design and statistical methods used should be given in the Methods section, as detailed in our Minimum Standards Reporting Checklist. Information essential to interpreting the data presented should be made available in the figure legends.<br><br>Have you included all the information requested in your manuscript?	Yes
**Resources**	Yes

A description of all resources used, including antibodies, cell lines, animals and software tools, with enough information to allow them to be uniquely identified, should be included in the Methods section. Authors are strongly encouraged to cite Research Resource Identifiers (RRIDs) for antibodies, model organisms and tools, where possible.<br><br>Have you included the information requested as detailed in our Minimum Standards Reporting Checklist?	
**Availability of data and materials**<br><br>All datasets and code on which the conclusions of the paper rely must be either included in your submission or deposited in publicly available repositories (where available and ethically appropriate), referencing such data using a unique identifier in the references and in the "Availability of Data and Materials" section of your manuscript.<br><br>Have you have met the above requirement as detailed in our Minimum Standards Reporting Checklist?	Yes

Main Manuscript

Click here to view linked References

Click here to access/download;Manuscript;Predomics_main_text_figures_on

Interpretable and accurate prediction models for metagenomics data

Authors / affiliations :

Edi Prifti *[,1,2], Yann Chevaleyre [3], Blaise Hanczar [4], Eugeni Belda [1], Antoine Danchin [5], Karine Clément [6,7], Jean-Daniel Zucker *[1,2]

[1] Institute of Cardiometabolism and Nutrition, ICAN, Integromics, Paris, France

[2] Sorbonne University, IRD, UMMISCO, UMI 209, Paris, France

[3] Paris-Dauphine University, PSL Research University, CNRS, UMR 7243, LAMSADE, Paris, France

[4] IBISC, University Paris-Saclay, University Evry, Evry, France

[5] Institute of Cardiometabolism and Nutrition, ICAN, Paris, France

[6] Sorbonne University, INSERM, Nutrition and Obesities; systemic approach research unit (NutriOmics), Paris, France

[7] Assistance Publique-Hôpitaux de Paris, Nutrition department, CRNH Ile de France, Pitié-Salpêtrière Hospital, Paris, France

Contact information*:*

Correspondence: Edi PRIFTI (e.prifti@ican-institute.org) and *Jean-Daniel ZUCKER (jean-daniel.zucker@ird.fr)[1] ; 50/52, bd Vincent Auriol, 75013, Paris, France*

Declaration of interest: none to declare

[1] Lead author

Abstract

Background: Microbiome biomarker discovery for patient diagnosis, prognosis and risk evaluation is attracting broad interest. Selected groups of microbial features provide signatures that characterize host disease states such as cancer or cardio-metabolic diseases. Yet, the current predictive models stemming from machine learning still behave as black boxes and seldom generalize well. Their interpretation is challenging for MDs and biologists, which makes them difficult to trust and use routinely in the physician–patient decision-making process. Novel methods that provide interpretability and biological insight are needed. Here, we introduce "*predomics*", an original machine learning approach, which is tailored for metagenomics data. It discovers not only accurate predictive signatures, but also provide unprecedented interpretability. Inspired by microbial ecosystem interactions, the decision provided by the predictive model is based on a simple, yet powerful score computed by adding, subtracting or dividing cumulative abundance of microbiome measurements.

Results: Tested on more than 100 datasets, we demonstrate that predomics models are extremely simple and thus highly interpretable. Even with such simplicity, they are as accurate as state-of-the-art methods. Moreover, the family of best models, discovered during the learning process, has the ability to distill biological information and help decipher the predictability signatures of the studied condition. Finally, in a proof-of-concept experiment, we successfully predicted body corpulence and metabolic improvement after surgery using pre-bariatric surgery microbiome data.

Conclusions: Collectively, this approach builds up both reliable and trustworthy diagnostic decisions while agreeing with societal and legal pressure that require explainable AI in the medical field.

Keywords:

prediction, interpretable models, metagenomics biomarkers, microbial ecosystems

Background

An increasing wealth of data from high-throughput molecular and imaging technologies is connecting biomedical sciences and machine learning (ML). The latter is impacting numerous areas of medicine, including disease diagnosis and prognosis [1-3]. It is now argued that ML and more globally artificial intelligence (AI), will dramatically improve prognosis within the coming years [4].

Simultaneously, progress made in high throughput technologies has contributed to developing new fields such as metagenomics. The association of the gut microbiota with human health and disease has been widely discussed [5] and links with numerous diseases are described [6-13]. Specifically, ecological relationships among bacterial species such as mutualism, parasitism, and competition [14] may change along with a shift in microbial equilibrium. Although these signatures allow predicting diseases, many of these findings are only correlative and require controlling for confounding factors — a task that remains challenging [15].

Metagenomics data must be interpreted carefully as they are often analysed in a small number of samples (N) compared to a very large number of variables (p). Current microbial catalogues, which are composed of millions of genes [16] and thousands of bacterial species and functional profiles [17], allow characterizing and comparing sampled ecosystems. Consequently, most models tend to overfit the training data and result in predictions arising from random sampling fluctuations. To reduce overfitting and allow for better generalization in unseen data, some authors use learning algorithms that include a dimension reduction or regularization methods, e.g. Elastic Net [11] or SVM-RFE [15]. While these algorithms are more straightforward than others, they generate complex models that are difficult to interpret. ML research has focused on building accurate models for large data collections, often at the expense of interpretability.

Providing an *explanation* of the prediction process is increasingly requested [18] when not mandatory [19], especially, in precision medicine [18, 19]. *Interpretable* models have two desirable properties: conciseness and readability by non-experts. They should contain simple operations and be limited in size [20-22]

Causality, as the holy grail of modern biology, is out of the scope of the interpretability property of a predictive model. Here, we investigated if models inspired by ecosystem relationships and sparse microbial signatures can be both accurate and more interpretable than more complex

well established state-of-the-art (i.e. SOTA) models, including logistic regression with elastic-net regularization (ENET) and support vector machines (SVM).

Data Description

We used public datasets to test our proposed algorithm *predomics* and compare with SOTA methods. For the classification tasks we used curated metagenomic datasets from ExperimentHub [23] (see supplementary material; **Table S1**). The code used to query and process the data is provided in the supplementary material package. In total, 54 datasets were derived (i.e. six different cohorts and for each six taxonomic levels, a marker gene and a pathway table along with a fused taxonomic dataset). They were also transformed as presence/absence for additional experiments (n=54).

Baseline microbiome data were also used to predict the clinical outcome of bariatric surgery on morbidly obese patients [24]. Their microbiome was sequenced at baseline, one, three, and twelve months after surgery (see original paper for methods).

Methods

The *predomics* optimization algorithm

Here, we propose a new family of models, named BTR for Binary/Ternary/Ratio. Learning optimal BTR models is computationally difficult. Because weights are discrete, usual techniques coming from convex optimization do not apply. A naïve way would be to perform an exhaustive search through the whole space of models. Unfortunately, this is not practically feasible since the computation time would increase exponentially with the number of features. The BTR learning problem is known as NP-Hard, which means that *no* algorithm can solve this problem exactly in polynomial time [25].

We can nevertheless apply heuristics that provides good models without guarantee on their optimality. Genetic algorithm (*i.e.* GA) is a stochastic optimization technique that can be of great use in such context. It adopts concepts from evolutionary biology — populations, reproduction, mutation and generations. The outline of the algorithm is described in supplementary materials. After the evolution process, a final population of predictive models is provided. The best model is obtained by applying a so-called *model-size penalization* ($accuracy_{penalized} = accuracy - \lambda k$), where k is the number of features in the model (i.e. parsimony) and λ is an hyperparameter controlling the penalization of the accuracy. Here, we

used ƛ=1% which means than a model that is using one additional feature will only be preferred if it improves the accuracy of more than 1%.

For classification, *predomics* may be set to optimize different parameters such as the accuracy (default), AUC, F1, precision or recall, while for regression it can optimize R^2 (default), rho or the standard error of the regression.

Experimental design

The experimental pipeline proceeds as follows:

1. Feature normalization: frequency tables are used as processed by Pasolli et al [23].
2. Features with low standard deviation are filtered out. The threshold corresponds to the maximum second derivative of the distribution of the feature's standard deviation.
3. The generalised performance of each method is estimated by 10-times 10-fold cross validation for the classification tasks and a 20-times 5-fold cross validation for the regression tasks.
4. The feature selection is embedded for the BTR models and Elastic Net. For SVM and RF, feature selection is based on the Mann-Whitney score as introduced in [26].
5. Algorithm performances are compared with a paired t-test using the 100 CV estimations. Those that are not significantly different (pval<0.05) are considered equivalent.

The BTR models are tested on 109 different datasets (see **Table S1**) and compared with the methods from the SOTA algorithms: support vector machine (SVM) with linear and Gaussian kernel (data not shown), Random Forest and Elastic Net (an improvement of Lasso, alpha=0.5). All algorithms were evaluated by measuring test accuracy in a cross-validation setting and compared among them using paired t-tests. A specific comparison between TerLog models (i.e. *ter* model with log-transformed data) and the geometric mean balance algorithm is provided in supplementary material.

Family of best models

A family of best models (i.e. FBM) is defined as the set of models returned by the algorithm, whose accuracy is within a statistically equivalent window, defined by a threshold assuming a binomial distribution (p<0.05). A FBM can be analysed in detail to distil biological information in the predictive context (see supplementary material).

Feature importance

Similar to RF, feature importance is defined as the usefulness of features to be predictive, given all other features and best models of the FBM. During each cross-validation fold, the out-of-bag error on each model of the FBM is computed. The importance of the jth feature is measured by permutating all features within the out-of-bag data. The out-of-bag error is computed on this perturbed data for each FBM model. The *importance score* for the jth feature is obtained by averaging over all FBM models the difference in out-of-bag error before and after the permutation. Finally, the mean decrease accuracy (MDA) is computed as the average of these values over all the folds and is displayed along with the standard error of the mean.

Regression models

Predomics can learn regression models, which are evaluated by maximizing either Spearman rho or Spearman R^2 or minimizing the scaled standard error of regression (SER). The model's score at this stage reflects the cumulative/difference/ratio of relative abundance of the species and needs to be scaled in the range of the variable to predict. Two additional parameters alpha (i.e. multiplication factor) and beta (i.e. intercept) are estimated.

Network reconstruction

We used Scalenet [27] to reconstruct the feature co-presence network in model selection data. Here we used the top 5% strongest edges inferred by *bayes_hc* and *aracne* methods in the FBM-presence table. ScaleNet first reduces the reconstruction problem into a number of simpler reconstruction problems, then employs state-of-the-art reconstruction methods to solve them. Finally, a consensual voting strategy between the methods is adopted to identify accurate sub-graphs, which are then overlapped together.

Results

A new family of models for metagenomics data

We propose a new family of models, named BTR for Binary/Ternary/Ratio, which are a simplification of linear models aiming at making their output even more interpretable. For each ecosystem $y_1 \dots y_n$, the abundance or presence of either genes, taxonomy levels, functions, or other microbial qualities, are represented by $X_1 \dots X_p$ predictor variables. In a linear model, a patient is predicted in a disease group with a probability of $p > 1/2$ if $\beta_0 + \sum_{j=1}^{p} \beta_j X_j > 0$,

where $\beta_0 \ldots \beta_p \in \mathbb{R}$ are real coefficients. The biological assumption is that the contribution of each bacterial species to the prediction is proportional to its abundance and that only a limited number of species is sufficient to support the prediction. BTR models are much simpler and are inspired by three hypotheses emphasizing relationships between species and associated ecosystem (**Figure 1**).

<u>Hypothesis 1</u>: The unweighted cumulative abundance of a group of species can predict disease state. We define the *binary models* (*i.e.* Bin) as linear models with the additional constraint that each coefficient $\beta_1 \ldots \beta_p$ (omitting the intercept β_0) must be binary — {0, 1} (**Figure 4A**). An example is in *(1)* and is interpreted as "if the cumulated abundance of *s_Veillonella_unclassified* and *s_Lachnospiraceae_bacterium_3_1_57FAA_CT1* is smaller than 9.7% of the total microbial abundance, then the individual is classified as healthy". These species may share the same ecological niche or interact directly with one another [28, 29].

(1) **If** s_Veillonella_unclassified **+** s_Lachnospiraceae_bacterium_3_1_57FAA_CT1 **<** 0.097
then class **=** healthy

<u>Hypothesis 2</u>: The difference of unweighted cumulative abundance of two groups of species can predict disease state. This assumption is implemented by *ternary models* (*i.e.* Ter), also linear models with the constraint that each coefficient $\beta_1 \ldots \beta_p$ (omitting the intercept β_0) be limited to the values {-1, 0, 1} (**Figure 4B**). An example of a ternary model in *(2) can be* interpreted as follows: "if the cumulative abundance of *s_Streptococcus_anginosus* and *s_Veillonella_unclassified* minus the abundance of *s_Alistipes_indistinctus* is greater or equal than 8.3 % of the total microbial abundance, then the patient is classified as healthy".

(2) **If** (s_Streptococcus_anginosus + s_Veillonella_unclassified) **-** s_Alistipes_indistinctus **≤** 0.083
then class **=** healthy

<u>Hypothesis 3</u>: The ratio of unweighted cumulative abundance of two groups of species can predict disease state. This assumption is implemented by *ratio models* (*i.e.* Ratio), also linear models with an additional constraint: each coefficient $\beta_1 \ldots$ □□ is limited to a value of $-\theta$, 0 or 1, where θ is a positive real number, and the intercept β_0 is set to zero (**Figure 4C**). An example in *(3)* can be interpreted as follows: "if the abundance of *s__Subdoligranulum_unclassified* is $\theta = 81$ times greater than the cumulative abundance of

s__Megasphaera_micronuciformis + *s__Streptococcus_anginosus* then the individual is classified as healthy".

(3) **If** $\frac{s\_Subdoligranulum\_unclassified}{s\_Megasphaera\_micronuciformis + s\_Streptococcus\_anginosus} > 81$
then class = healthy

Biologically, both Ter and Ratio models can correspond to interactions of different types of species including cooperation and competition among species. BTR models can be illustrated as balances, where species abundance is symbolized by the cumulative weights (**Figure 1**). The concept of balance is not new in ecology and was first proposed to address the compositionality problem in microbiome data. A balance-based representation can bypass this issue and reveal pertinent biological patterns [30]. Very recently, other authors have applied the balance representation in the classification context [31]. Here, we propose a more general framework of models that encompass such balances. Indeed, they would correspond to our Ter models when applied to log-transformed data — named TerLog (see supplementary material; **Figure S12**). Learning linear models on log-transformed relative abundance data corresponds to identifying balances of multiplicative relationships. However, which characterizes best microbial ecosystems (i.e. multiplicative or additive), remains an open question. We propose here different types of models that could be useful in tackling such questions. The *predomics* algorithm was developed to specifically learn BTR models.

BTR models are sparse, accurate and improve with taxonomic specificity

We tested our approach on six different public metagenomic datasets (**Table S1**) and nine derived types of variables, (six different taxonomic levels, a merged multi-taxonomic level, marker genes and a functional MetaCyc pathway table, *i.e.* a total of 54 datasets, see methods). We trained and tested models with different number of features (*i.e.* model-size, k_#) and noticed an effect on accuracy. As expected, the testing performance on unseen data was lower compared to training performance. However, this difference was more pronounced for the SOTA, indicating a significant overfitting effect, compared with BTR models. The simplicity and sparsity of the BTR models reduces overfitting on studied datasets (**Figure 2**). As BTR models come with an embedded feature-selection strategy, we used a Mann-Whitney test to select the k_# most correlated features for RF and SVM to allow comparison. For ENET we used the embedded regularization path and selected the first k_# form it.

We applied a model-size penalization technique on the empirical (training) accuracy to select the best model. BTR models performed at least as well as the SOTA in 46/54 (85%) of the cases. They outperformed SOTA in 19/54 (35%) and were outperformed in 8/54 (15%) (**Figure 3; Figure S1A-C)**. Similar results were observed even when all the variables in the dataset were used (no-penalization) for the SOTA (**Figure S2A-C**) or when fixing the same model size for all the compared models (**Figure S3A-C**).

When learning from the different types of variables based on taxonomic levels (Cirrhosis stage-1), the performance of the models, vary accordingly. Higher performance is obtained at the gene marker, species and genus levels, and decrease with higher taxonomic levels. Moreover, when applied to a multi-taxonomic level dataset (*strain* to *phylum* as generated by Pasolli et al. [23] with different specificity levels mixed together; *i.e. whole tax*), models displayed surprisingly good performance (**Figure 3B**). Indeed, in this space, models can be powerful as they can summarize more complex rules such as: "**if** (abundance of all Firmicutes **–** abundance of all Clostridiales order) **>** threshold **then** disease*"*.

We tested the generalization of Bin, Ter, Ratio and also TerLog models trained in Cirrhosis stage-1, in a second, independent dataset (i.e. Cirrhosis stage-2). Results illustrated in **Figure S5** indicate very good external validation with an average training accuracy=0.89 (sd=0.02) and testing accuracy=0.85 (sd=0.04). Ter and Ratio models generalized better compared to Bin and TerLog (see supplementary material).

In addition to the abundance datasets described above, we trained and tested similar models on *presence/absence* binary data derived from the previous 54 abundance datasets. Overall results are similar indicating that the detection of species alone can be powerful enough in prediction tasks (see supplementary material; **Figure S1D-F; Figure S2D-F; Figure S3D-F; Figure S4**). Noteworthy, when applied to presence data, BTR models indicate relationships between sub-ecosystem complexity or richness. These can be useful to detect switch-like mechanisms in the microbiome.

BTR models generate straightforward interpretations in contrast to state-of-the-art models

A graphical barcode representation illustrates the simplicity of BTR models. In **Figure 4A-C** *left*, the models are represented by red and blue horizontal lines, corresponding respectively to positive and negative coefficients (either 1 or -1). The same representation is used to visualise

the normalized coefficients of ENET and SVMLIN models (the line length is proportional to the coefficient in the interval [-1,1]) (**Figure 4D-E**). For the RF model only one of the 500 decision-trees used in the model is illustrated (**Figure 4F**). Additionally, for each variable selected by BTR models, we assessed their importance in prediction, using a variant of the well-known mean decrease accuracy (MDA) (**Figure 4A-C** *middle*). The feature importance (FI) score of BTR models correlates strongly with the FI of the well-established but more complex RF model (respectively R=0.68, R=0.81, R=0.7, with bin, ter and ratio models; **Figure S9, S10**). This information allows prioritizing further exploration of the features in the context of the predicted phenomenon.

Predomics generates a family of BTR models with equivalent predictive power in a given model-size range (*i.e.* FBM for family of best models; **Figure S6**; see methods and supplementary material). FBM is analysed to identify the common features that are found in the models. For instance, in the cirrhosis stage-1 (species) dataset, the 268 models in the FBM with model-size<6 rely only on 67 features (i.e. 16% of the whole dataset), which can be used to infer the *feature co-presence network in models* (**Figure 5A**). An emerging property of this network is the clustering of phylogenetically related species, such as Firmicutes species enriched in patients (blue tones) and Proteobacteria and Actinobacteria enriched in controls (green tones). Co-presence of the features indicate complementarity in prediction (red edges), while replacement of the features by one another indicate redundancy (blue edges). This can also be observed with the inversed relation of feature pairs in the data and in the models (**Figure 5C)** — the most correlated pairs in the dataset are those that don't occur together in the models. This network provides precious information to decipher the sub-ecosystem that is the most associated with the disease (**Figure S6-S8**).

BTR models provide biological insights

We focused on the liver cirrhosis dataset [7], where major patient dysbiosis was observed with decreased microbial richness, depletion of gut commensals, and an invasion of oral bacteria. Several markers at taxonomic and functional levels were associated with the disease.

Some authors have modelled liver cirrhosis associated microbiome using curated information from the literature, such as the ratio of autochthonous (butyrate-producer bacteria) to non-autochthonous (oral bacteria, opportunistic pathogens). They used these taxa to build a cirrhosis dysbiosis ratio (CDR) score [32]. Based on their description we built three redundant ratio models using family taxonomic features to reproduce their score and applied them in the

liver cirrhosis stage 1 (family) dataset [7] (**Figure 6B-D**). We searched the same family-level dataset for Ratio models, which provided superior performance (accuracy=0.86; **Figure 6A**) compared with CDR-based models (accuracy=0.56 in average; **Figure 6F**). The reason for the CDR lower performance can be explained by the inclusion of the *Bacteroidaceae* family in the liver cirrhosis group by the authors, while we observe the opposite association in the current dataset. *Bacteroidetes*-related features are enriched in the control group and this is consistent for different taxonomic levels (**Figure 6E**, see supplementary material).

At the phylum level, the Ratio model *(S6)* points at a mutual exclusion between Bacteroidetes and the combination of Proteobacteria and Viruses, which is also picked up by the Bin model *(S4)*. These models are in line with a decrease in Bacteroides and an increase in Proteobacteria and Fusobacteria in the liver cirrhosis group, reported in the original study. The decrease in Bacteroidetes indicates a decrease in highly prevalent gut bacteria, whereas the increase of Proteobacteria has been repeatedly reported in dysbiotic microbiomes of patients and has been associated with chronic inflammation and serum lipopolysaccharides [33, 34]. The Virus prevalence in the liver cirrhosis group, may reflect the oral microbiome signature or increased incidence of viral infections together with opportunistic pathogens.

The potential competition between oral and gut microbes reported in previous studies [35] is best reflected by Ter and Ratio models with genus abundance data, that combine *Veillonella* (oral bacteria; opportunistic pathogen) enriched in liver cirrhosis at one side and *Bacteroides* plus *Eubacterium (S9)* or *Coprococcus (S8)* enriched in controls. The latter represent butyrate producers (*Coprococcus* and *Eubacterium*) and complex polysaccharide degraders (*Bacteroides* genus) [36]. Among the most important genera in the FBM we find *g__Veillonella*, *g__Streptococcus*, *g__Haemophilus*, *g__Coprococcus* and *g__Lactobacillus*, all more abundant/prevalent in patients.

Best Ratio and Ter models *(1-3)*, include oral bacterial species of the genus *Veillonella (Veillonella_unclassified)*, *Streptococcus (S. parasanguinis and S. anginosus)* and opportunistic pathogens like *Megasphaera micronuciformis* that proliferate in liver cirrhosis patients, whereas butyrate producers of the genus *Subdoligranilum* (*Subdoligranilum unclassified*) closely related to *Faecalibacterium prausnitzii [37]* and complex polysaccharides degrading species like *Bacteroides cellulosilyticus [38]* characterize control subjects. *Megasphaera micronuciformis* was previously associated with primary biliary cirrhosis based on 16S rRNA quantification [39]. A more in-depth exploration of the FBM (**Figure S6-S8**)

and the feature-model co-occurrence network (**Figure 5**) delineates detailed relations of the predictive sub-ecosystem.

At the functional level, predictive models *(S10-S12)* from MetaCyc pathway abundance data include pathways that suggest an increased aerobic metabolism (HEMESYN2−PWY: heme biosynthesis II (anaerobic), essential for cytochromes and heme-containing globins, PWY−922: mevalonate pathway I, needed for the biosynthesis of ubiquinone and menaquinone complexes of respiratory chains). Interestingly, increase in aerobic respiration profiles has also been identified as metabolic signatures of inflammation-associated dysbiosis in models of colitis [40]. Moreover, we observe the presence of modules related with bacterial peptidoglycan biosynthesis in the FBM (PWY−6470: peptidoglycan biosynthesis V). It has been described as an elicitor of inflammatory response associated to the progression of liver cirrhosis [41], in agreement with a more inflammatory profile of cirrhotic patients.

Altogether, these results indicate that BTR models discover important features with relevant biological information. BTR models are more accurate then literature-based ones and have the ability to distil and capture the predictive biological information embedded in the data.

Discussion

In principle, BTR models could be applied to any type of data. However, they are best suited to *commensurable* measurements (*i.e.* variables measurable by the same standard or measure). In the growing field of metagenomics, issues related to compositionality and data processing still remain to be solved. Recent work has shown the importance of data acquisition in subsequent analytical inferences. In particular, microbial loads differ significantly between individuals and are associated with specific types of microbial ecosystems [42]. An advantage of the *Ratio* models is that they are scale-invariant given they do not depend on absolute measurements, thus avoiding compositionality issues. Moreover, varying sequencing depth, can be an issue in over or under estimating less abundant taxa that can find themselves in the models. It is thus advisable to pre-filter rare taxa from the dataset, before training the models. However, the sparsity constraint, will force important taxa to be selected, improving the generalization of the models.

The simplicity of a BTR model may come with the risk of over-interpretation. The existence of *k* species in a model, may correspond to different explanations ranging from simple correlation to causal relation. They may or may not interact together, as in the case of a niche

differentiation [31]. For instance, the buccal-originated species found in the gut of liver cirrhosis patients [7] along with the absence of commensals may reflect a global difference in the environment where they live rather than direct interaction [7]. Even if BTR models represent real interactions between species, it is not recommended to give a causal interpretation without experimental validation. Nevertheless, identifying such species provides important knowledge towards understanding potential mechanisms between species or between species and the host.

The quality of reference datasets used in building predictive models is crucial for model interpretability. The propagation of errors and inaccuracies in genomic datasets is a well-known issue, and affects automated methods for functional annotation [43]. Moreover, due to the lack of biochemical characterization of orphan enzymatic activities, the number of sequences with unknown functions is extremely large, making error percolation of common feature [44] (see supplementary material).

One important issue with microbiome studies resides in the potential confounders modulating microbial ecosystems. For instance, it has been shown that metformin can alter the bacterial ecosystem such that some bacterial species (*e.g. E. coli*) are increased in abundance while others are depleted [45]. It is thus important to filter out confounder-related species from the data or to filter out models that are sensitive to confounders.

Finally, besides quantifying taxa abundance through whole shotgun or 16S rRNA sequencing, BTR models can be used to develop specific acquisition technologies such as microarray DNA chips or qPCR-based tests, built with primers that are specific to the species/taxa found in the models [46]. From a clinical perspective, because BTR models rely on a small number of variables, quantifying a relatively small subset of variables (genes, species, pathways, OTUs, etc) can be sufficient to simultaneously predict multiple tasks. Such applications, after being properly validated, will be important to the medical community in their translational quest in improving patient care. Our approach brings us a step closer towards useful clinical predictions while preserving interpretability.

Potential implications

In our article, we propose an original Machine Learning method, called *predomics*, which is tailored for metagenomics data but can be applied to other fields as well. We strongly believe that this original approach will have significant impact on both the development of predictive

models based on metagenomics data as well as their applications to medical conditions. This approach will support clinical decisions in the context of precision medicine. The interpretability of the models will ease acceptability and suggest candidates for microbiome targeted treatments. Moreover, it will serve as a bridge to further develop cross-fertilization between AI, biology and precision medicine.

Availability of source code and requirements

Supplementary information and source data files are available online and the *predomics* package in *https://git.integromics.fr/published/predomics*. The software is registered in the scicrunch.org repository under the RRID: SCR_017415.

Financial support:

This work was also supported by the French National Agency through the national program Investissements d'Avenir (reference no. ANR-10-IAHU-05) IHU ICAN; by the Funding Support of European Union's Seventh Framework Program under grant agreement HEALTH-F4-2012-305312, by the Assistance Publique-Hôpitaux de Paris promoter of the clinic program and by Assistance Publique-Hôpitaux de Paris Contrat d'interface chercheurs 2015-2018. We wish to thank S.D. Ehrlich as well E. Le Chatelier for mindful discussions on the early stages of this work and T. Swartz for help in language proofreading.

Authors contributions:

EP: overall conception, design and interpretation; designing and coding the software; conducting all experiments; writing the manuscript. **YC** conception and interpretation of the approach; coding; drafting the manuscript. **BH**: conception, design and interpretation of the approach; coding; drafting the manuscript. **EB**: biological interpretation of the results; drafting the manuscript. **AD**: biological interpretation of the results; drafting the manuscript. **KC**: data production (bariatric model); biological interpretation of results. **JDZ**: conception, early prototyping, design and interpretation of results; drafting the manuscript. All authors approved the manuscript.

REFERENCES

References

1. Buch, V.H., I. Ahmed, and M. Maruthappu, *Artificial intelligence in medicine: current trends and future possibilities.* The British journal of general practice : the journal of the Royal College of General Practitioners, 2018. **68**(668): p. 143-144.
2. Krittanawong, C., et al., *Artificial Intelligence in Precision Cardiovascular Medicine.* Journal of the American College of Cardiology, 2017. **69**(21): p. 2657-2664.
3. Reynolds, R.J. and S.M. Day, *The growing role of machine learning and artificial intelligence in developmental medicine.* Developmental Medicine Child Neurology, 2018. **59**(Suppl. 3): p. 36-1.
4. Obermeyer, Z. and E.J. Emanuel, *Predicting the Future — Big Data, Machine Learning, and Clinical Medicine.* New England Journal of Medicine, 2016. **375**(13): p. 1216-1219.
5. Walsh, C.J., et al., *Beneficial modulation of the gut microbiota.* FEBS Letters, 2014. **588**(22): p. 4120-4130.
6. Ley, R.E., et al., *Obesity alters gut microbial ecology.* Proceedings of the National Academy of Sciences, 2005. **102**(31): p. 11070.
7. Qin, N., et al., *Alterations of the human gut microbiome in liver cirrhosis.* Nature, 2014. **513**: p. 59.
8. Wen, L., et al., *Innate immunity and intestinal microbiota in the development of Type 1 diabetes.* Nature, 2008. **455**(7216): p. 1109-1113.
9. Qin, J., et al., *A metagenome-wide association study of gut microbiota in type 2 diabetes.* Nature, 2012. **490**: p. 55.
10. Elson, C.O. and Y. Cong, *Host-microbiota interactions in inflammatory bowel disease.* Gut Microbes, 2014. **3**(4): p. 332-344.
11. Zeller, G., et al., *Potential of fecal microbiota for early-stage detection of colorectal cancer.* Molecular Systems Biology, 2014. **10**(11): p. 766-766.
12. Le Chatelier, E., et al., *Richness of human gut microbiome correlates with metabolic markers.* Nature, 2013. **500**(7464): p. 541-546.
13. Cotillard, A., et al., *Dietary intervention impact on gut microbial gene richness.* Nature, 2013. **500**(7464): p. 585-588.
14. Faust, K. and J. Raes, *Microbial interactions: from networks to models.* Nature Reviews Microbiology, 2012. **10**(8): p. 538-550.
15. Forslund, K., et al., *Disentangling disease and drug signatures of the human gut microbiome: the case of type 2 diabetes mellitus.* 2015: p. 1-30.
16. Li, J., et al., *An integrated catalog of reference genes in the human gut microbiome.* Nature biotechnology, 2014.
17. Nielsen, H.B.o.r., et al., *Identification and assembly of genomes and genetic elements in complex metagenomic samples without using reference genomes.* Nature biotechnology, 2014: p. 1-11.
18. Martens, D., et al., *Performance of classification models from a user perspective.* Decision Support Systems, 2011. **51**(4): p. 782-793.
19. Goodman, B. and S.R. Flaxman, *European Union Regulations on Algorithmic Decision-Making and a "Right to Explanation".* AI magazine, 2017.
20. Ustun, B. and C. Rudin, *Optimized Risk Scores*, in *Proceedings of the 23rd ACM SIGKDD International Conference on Knowledge Discovery and Data Mining*. 2017, ACM: Halifax, NS, Canada. p. 1125-1134.
21. Tibshirani, R., *Regression shrinkage and selection via the lasso.* Journal of the Royal Statistical Society. Series B (Methodological), 1996: p. 267-288.
22. Tibshirani, R.J., *In praise of sparsity and convexity.* Past, Present, and Future of Statistical Science. Chapman Hall, London, 2014.
23. Pasolli, E., et al., *Accessible, curated metagenomic data through ExperimentHub.* Nature methods, 2017. **14**(11): p. 1023-1024.

24. Aron-Wisnewsky, J., et al., *Major microbiota dysbiosis in severe obesity: fate after bariatric surgery.* Gut, 2018.
25. Chevaleyre, Y., F. Koriche, and J.-D. Zucker, *Rounding Methods for Discrete Linear Classification.* Journal of Machine Learning Research, 2013. **28**(1): p. 651–659.
26. Pasolli, E., et al., *Machine Learning Meta-analysis of Large Metagenomic Datasets: Tools and Biological Insights.* PLoS Computational Biology, 2016. **12**(7): p. e1004977.
27. Affeldt, S., et al., *Spectral consensus strategy for accurate reconstruction of large biological networks.* BMC bioinformatics, 2016. **17**(Suppl 16): p. 493.
28. Freilich, S., et al., *Competitive and cooperative metabolic interactions in bacterial communities.* Nature Communications, 2011. **2**(1): p. 589.
29. Connor, E.F. and D. Simberloff, *The Assembly of Species Communities - Chance or Competition.* Ecology, 1979. **60**(6): p. 1132-1140.
30. Morton, J.T., et al., *Balance Trees Reveal Microbial Niche Differentiation.* mSystems, 2017. **2**(1): p. e00162-16-11.
31. Rivera-Pinto, J., et al., *Balances: a New Perspective for Microbiome Analysis.* mSystems, 2018. **3**(4): p. 174-12.
32. Bajaj, J.S., et al., *Altered profile of human gut microbiome is associated with cirrhosis and its complications.* Journal of Hepatology, 2014. **60**(5): p. 940-947.
33. Krajmalnik-Brown, R., et al., *Effects of Gut Microbes on Nutrient Absorption and Energy Regulation.* Nutrition in clinical practice : official publication of the American Society for Parenteral and Enteral Nutrition, 2012. **27**(2): p. 201-214.
34. Liu, Q., et al., *Synbiotic modulation of gut flora: Effect on minimal hepatic encephalopathy in patients with cirrhosis.* Hepatology, 2004. **39**(5): p. 1441-1449.
35. Winer, B.Y. and A. Ploss, *Breaking the species barrier for hepatitis delta virus.* Hepatology, 2015. **63**(1): p. 334-336.
36. Shreiner, A.B., J.Y. Kao, and V.B. Young, *The gut microbiome in health and in disease.* Current Opinion in Gastroenterology, 2015. **31**(1): p. 69-75.
37. Holmstrøm, K., et al., *Subdoligranulum variabile gen. nov., sp. nov. from human feces.* Anaerobe, 2004. **10**(3): p. 197-203.
38. Robert, C., et al., *Bacteroides cellulosilyticus sp. nov., a cellulolytic bacterium from the human gut microbial community.* International Journal of Systematic and Evolutionary Microbiology, 2007. **57**(7): p. 1516-1520.
39. Lv, L.-X., et al., *Alterations and correlations of the gut microbiome, metabolism and immunity in patients with primary biliary cirrhosis.* Environmental Microbiology, 2016. **18**(7): p. 2272-2286.
40. Hughes, E.R., et al., *Microbial Respiration and Formate Oxidation as Metabolic Signatures of Inflammation-Associated Dysbiosis.* Cell Host and Microbe, 2017. **21**(2): p. 208-219.
41. Shen, T.C., et al., *Engineering the gut microbiota to treat hyperammonemia.* J Clin Invest, 2015. **125**(7): p. 2841-50.
42. Vandeputte, D., et al., *Quantitative microbiome profiling links gut community variation to microbial load.* Nature, 2017: p. 1-21.
43. Poptsova, M.S. and J.P. Gogarten, *Using comparative genome analysis to identify problems in annotated microbial genomes.* Microbiology, 2010. **156**(Pt 7): p. 1909-17.
44. Hanson, A.D., et al., *'Unknown' proteins and 'orphan' enzymes: the missing half of the engineering parts list--and how to find it.* Biochem J, 2009. **425**(1): p. 1-11.
45. Forslund, K., et al., *Disentangling type 2 diabetes and metformin treatment signatures in the human gut microbiota.* Nature, 2015: p. 1-12.
46. Burke, H.B., *Predicting Clinical Outcomes Using Molecular Biomarkers.* Biomarkers in Cancer, 2017. **8**: p. BIC.S33380-11.

Figure Legends

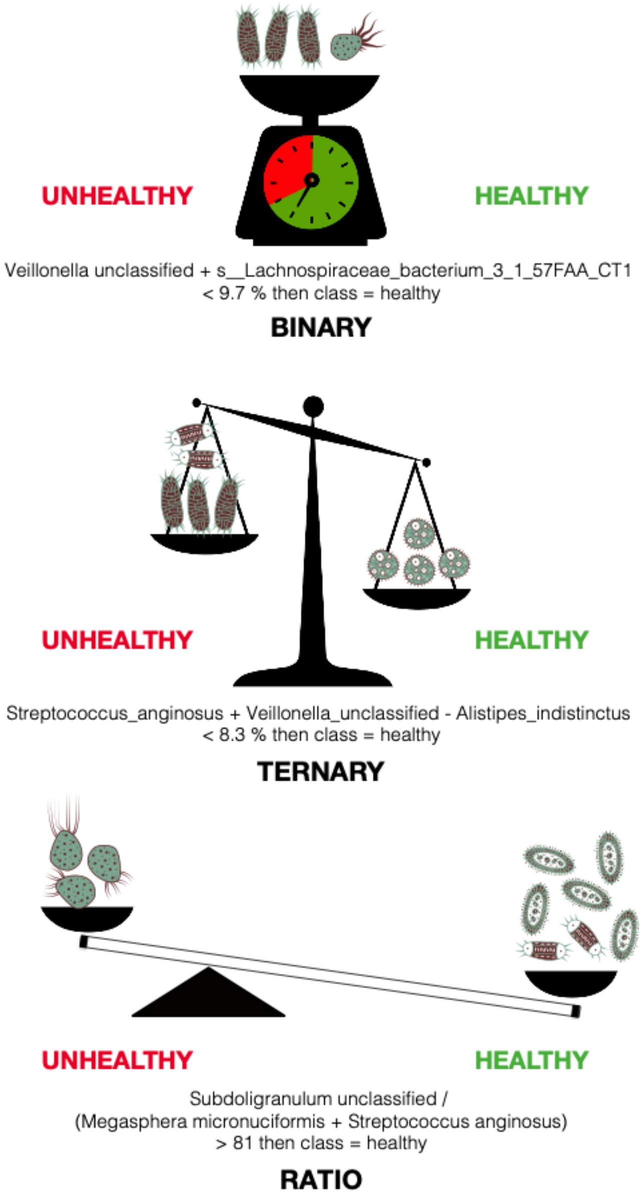

Figure 1: The three balance concepts depicting the BTR models

Top: The Binary model tests whether the cumulated abundance of a set of species is below or above a certain threshold. *Middle*: The Ternary model tests whether the cumulated abundance of a first set of species is below or above the cumulated abundance of a second set of species plus a certain threshold. *Bottom*: The Ratio model tests whether the cumulated abundance of a first set of species over the cumulated abundance of a second set of species is above a given threshold.

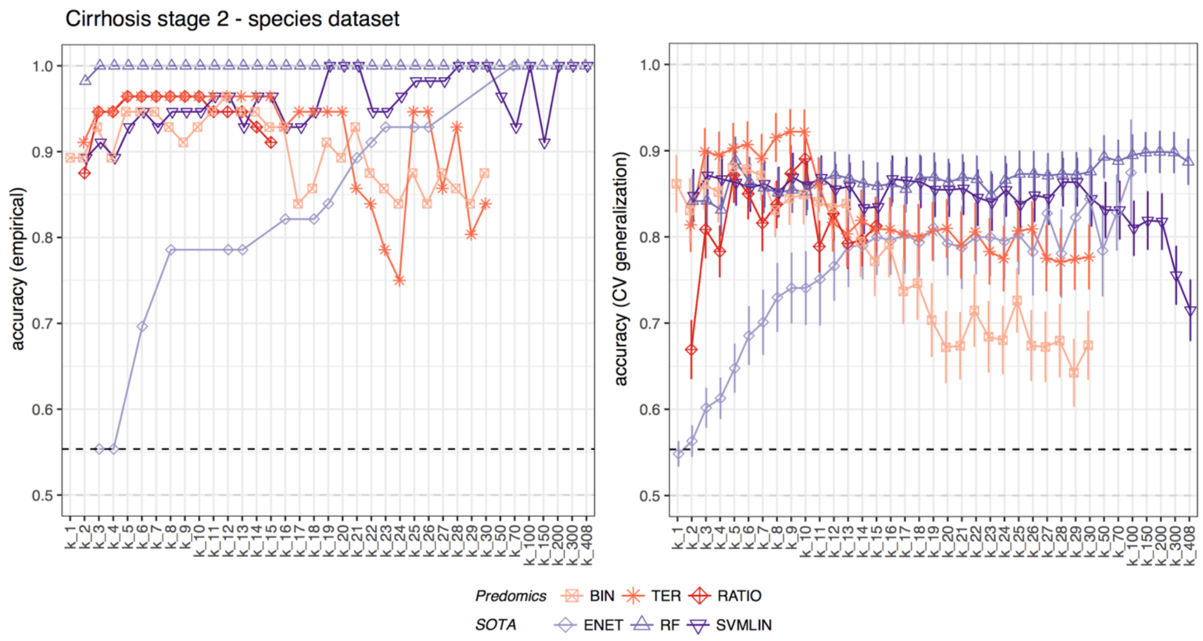

Figure 2: Model performance across different model-size

Left: Training accuracy of the best models (on the y-axis) in the Cirrhosis stage-2 dataset for different model-size *k* (indicated *k_#* on the x-axis). *Right:* Testing accuracy of the best models for each model-size as the average of 10-times, 10-fold cross validation sets +/- standard error of the mean. Dashed line indicates the majority class (i.e. the accuracy obtained when simply predicting the majority class through chance alone).

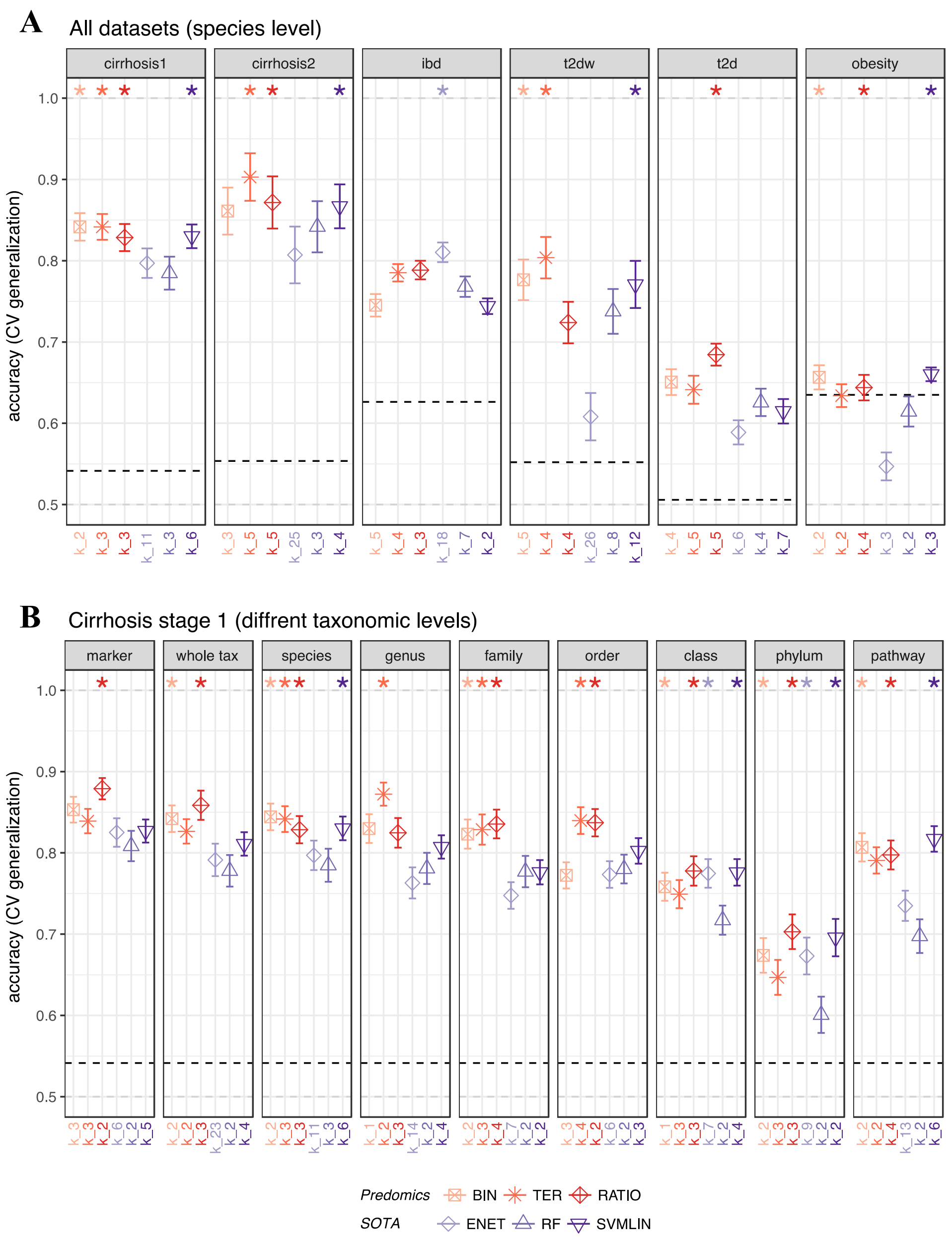

Figure 3: BTR models vs. SOTA performance across different diseases and taxonomic levels

A: Accuracy measured in the test datasets at the species level across six different datasets. The * on top indicate whether the corresponding BTR or SOTA algorithms are significantly better than others (*i.e.* without stars). **B**: Accuracy measured in the test datasets in different taxonomic levels of gut microbiome quantification (*species*, *genus*, *family*, *order*, *class and phylum*, *whole taxonomy*) as well as in marker gene and pathway abundance tables. Dashed bars indicate the majority class and *k_#* indicates the model-size. A 10-times 10-fold validation test values are summarized as mean +/- standard errors.

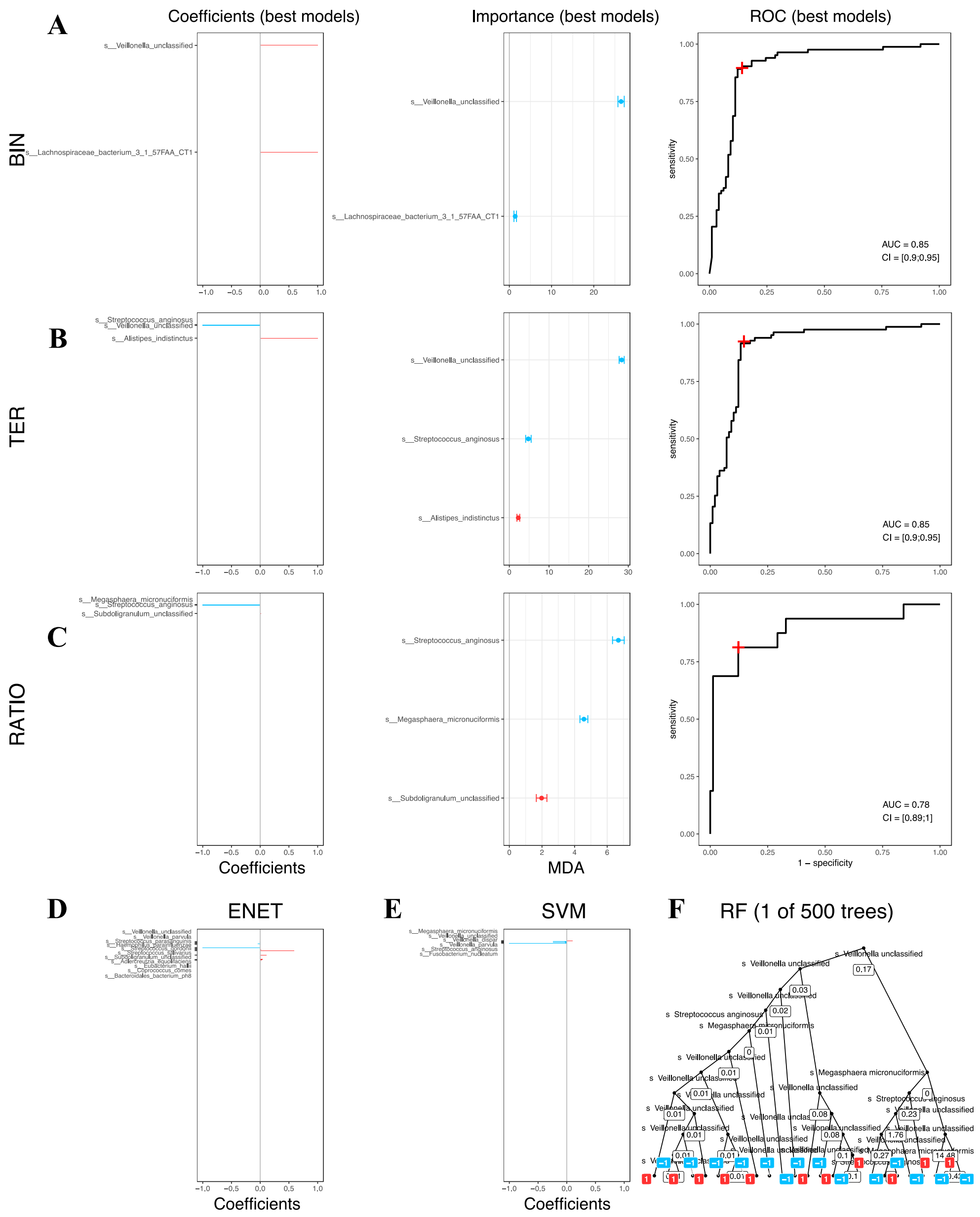

Figure 4: BTR models are interpretable compared to state-of-the-art

A-C *left*: Barcode graphical representations indicating the coefficients (1 or -1) of the BTR model features sorted by decreased correlation strength with the class to predict. **A-C** *middle*: Mean decrease accuracy (MDA) plots indicating feature importance computed during the cross-validation process. Blue and red colours indicate enrichment in patients and controls respectively. **A-C** *right*: Receiver operator characteristic (ROC) plots for the same BTR models. The red cross indicates the specificity and sensitivity of the model. **D-F**: A visualisation attempt of the SOTA models with barcode plots indicating the coefficients (values in [-1,1]) for ENET and SVMLIN, and only one tree out of the 500 used in the RF model.

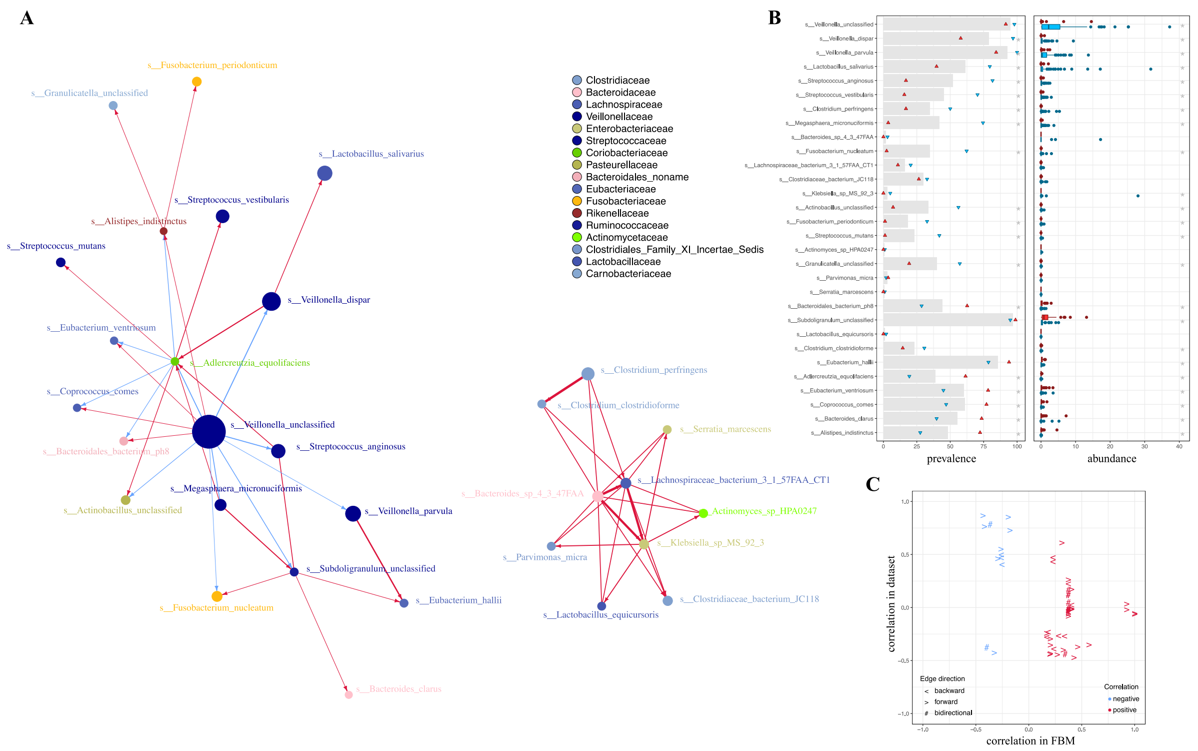

Figure 5: **Feature co-occurrence network in the family of best models**

A: This network displays feature co-occurrence patterns in FBM models. Only the top 5% strongest edges inferred using the ScaleNet network reconstruction approach (parameterized with *bayes_hc* and *aracne* algorithms, see methods) are shown. The size of the nodes is proportional to the average importance (MDA) in the BIN, TER and RATIO experiments. The colours of the nodes indicate the taxonomic family assignation as indicated in the legend. The red and blue edges indicate co-presence and co-absence in the models respectively. **B**: For each feature present in the network we show on the *left*: the prevalence of the features in the whole dataset (grey bar) and in the prediction classes (disease, healthy) depicted as blue and red dots respectively and in the *right*: the feature abundance distribution in the prediction classes (disease, healthy) depicted as blue and red box plots respectively. Grey stars indicate significant differences. **C**: A scatter-plot indicating for each edge of the network the correlation between the two features in the data and FBM respectively in the y- and x-axis. The colour is the same as for the edges in the network while the shape indicates the direction of the edges in the network.

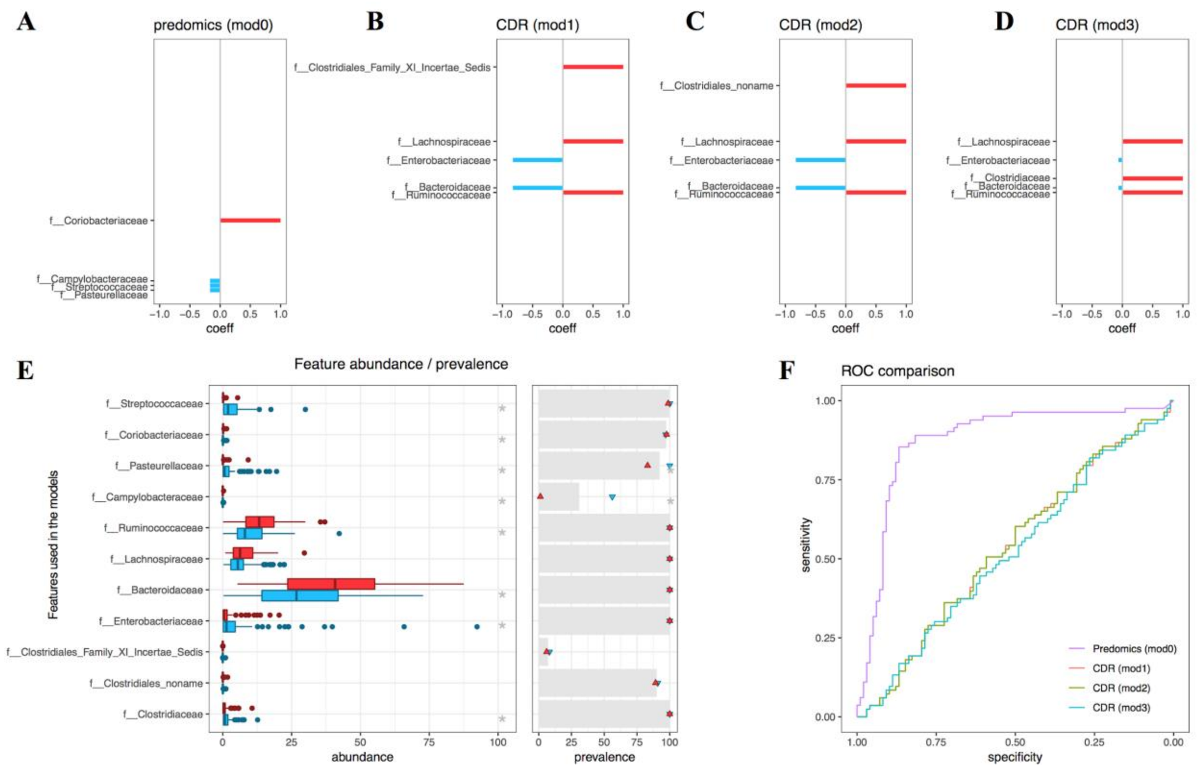

Figure 6: **Cirrhosis Dysbiosis Ratio (CDR) index compared to *predomics* ratio model.**

A-D: Barcode plots indicating the coefficients of the Ratio models *(S13-S15)* build with features from the CDR index and *predomics* discovered model *(S16)*. Red and blue colours indicate respectively the numerator and denominator of the ratio model and are respectively enriched in the controls and liver cirrhosis patients. The length of the lines is proportional to the ratio factor optimized in the model. **E** *left*: Boxplots indicating the abundance distribution by class for all features used in these models (red is enriched in controls and blue in the liver cirrhosis group). *right*: for the same features the prevalence of non-zero values is depicted in grey for the whole cohort and red and blue dots respectively in the control and patient groups. Grey stars indicate significant difference. **F**: Receiver operating characteristic (ROC) curves for the four models *(S13-S16)*.

Click here to access/download
Supplementary Material
Predomics_Supplementary Material_GigaScience_revised.docx

URL for supplementary package

Click here to access/download
Supplementary Material
Supplementary package predomics address.pdf

Click here to access/download;Personal Cover;Predomics_Cover_letter_And_Response_To_Reviewers

Dear Dr. Nogoy,

Please accept our gratitude in allowing our work to be reviewed in Gigascience and accompanying us during this process.

We have carefully examined both reviews and have tried our best to answer them point-by-point and in a constructive manner. We have performed additional analyses to answer some of the questions raised by the reviewers and improved upon their advice both the revised version of the manuscript as well as the supplementary material.

Moreover, we registered the *predomics* software in the scicrunch.org repository as requested under the accession number RRID: SCR_017415 and mentioned it in the revised version of the manuscript (page 14).

We hope that both the reviewers and the editorial board will find our answers and the new version of the manuscript satisfying.

On a more general note, we would like to stress that we particularly appreciate the principles to which the journal Gigascience adheres and that they guided us in the choice to submit a manuscript.

With our best regards
Edi Prifti and Jean-Daniel Zucker

Reviewer reports:

Reviewer #1

In this paper, the author provided a machine learning approach named Predomics, aiming to conduct microbiome biomarker discovery. In comparison to popular machine learning methods including support vector machines and elastic-net regularization, it was based on simple biological principles which meant it was interpretable. With experiment on 109 datasets, Predomics achieved comparable accuracy with state-of-art methods like SVM in some conditions. Besides feature extracting, author further constructed feature co-presence network in models to show the biological interpretation behind selected features.
Major concern about the result:
(1) It could be observed from figure 2 that in the training set, the accuracy of Predomics fluctuated a lot as the model size grew. In the paper, author regarded it represented Predomics was able to avoid overtraining. However, the fluctuation or decrease in accuracy was much too severe in large scale model. For example, the accuracy of TER in Predomics dropped to 75% in k_24. I wonder whether it means that Predomics method are not capable of predicting in large scale model.

We thank the referee for having reviewed our work as well as for the accurate observations. As a short reminder, one of the main objectives of the predomics approach is precisely to avoid "large scale models". Indeed, we search for simple models that are both accurate and interpretable. Their simplicity relies on two aspects, i) <u>the coefficients,</u> which are drawn from the ensemble {-1,0,1}, and ii) <u>the sparsity</u> of the model (i.e. the smallest possible number of features k).

*The reviewer's observation that fluctuation or decrease in accuracy is more important as models get bigger is related to the search algorithm as well as the set of parameters optimized for small models. Indeed, predomics uses an implementation of genetic algorithms (detailed in the Methods section), which evolves populations of n = models (n is usually set between 100 to 1000) during a number of generations (typically 100). The population includes models with different sparsities (i.e. number of variables 1:30 used in the models presented in the paper). The combinatorial space increases exponentially with the sparsity k of the model. In this example of the Cirrhosis-2 (species) dataset, there are 408 features. The combinatorial space of models in size k=1 will be 408, for size k=2 will be 83028 and so on up to size k=30, which will be 2.63e+45. We only explore tiny parts of these spaces to find the best models (at most 1000*100 including duplicated models that are kept from one generation to the next). We have shown graphically in **Figure R1** (and **Figure S9** of the revised supplementary material) in log10 scale the size of the unexplored combinatorial space. This is why for some higher k-sparsities we have found "good models" and for others not so good.*

Moreover, when the sample size is small (as it is typically the case in metagenomics), small linear models (and small BTR models) can precisely classify the data. Thus, we do not have enough information to distinguish between small and larger models. To accurately learn larger models (e.g. with more than 24 non-zero coefficients), we would simply need more data (observations).

Predomics can be used to find good large-scale models, but It would need changing the parameters of the algorithm (bigger population size, higher number of generations in the evolution process, higher mutation rates, etc.) to converge faster towards better local minima in these larger spaces. Nevertheless, in the context of this paper we are interested in a fast method that identifies good small-scale models.

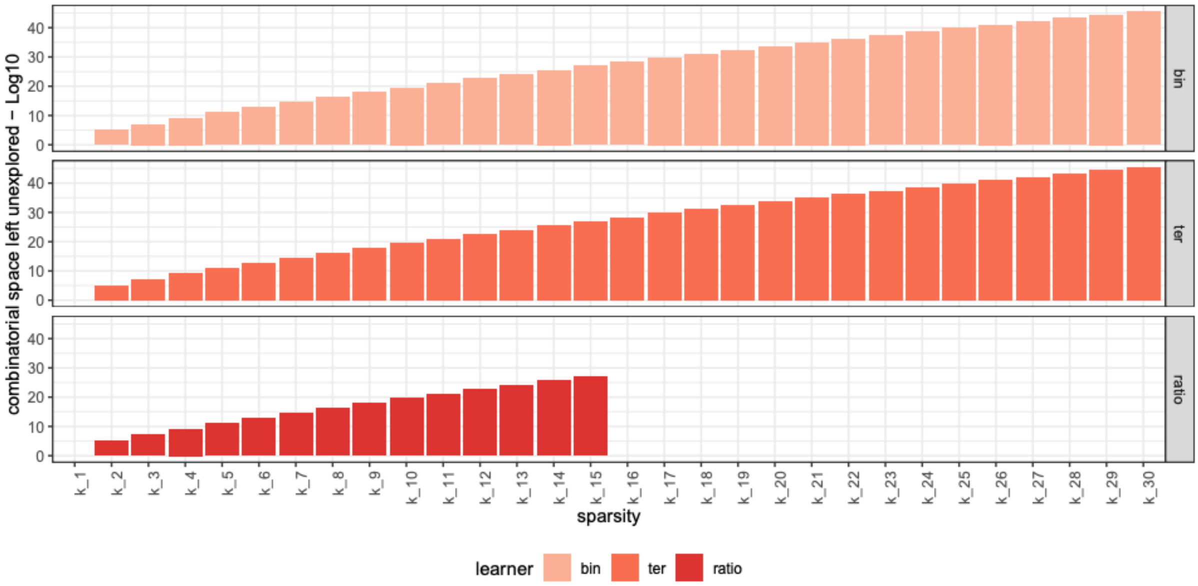

Figure R1: Combinatorial space left to explore for each k-sparsity of the model.
Data from the Cirrhosis-2 (species) dataset are used here, with 408 bacterial features.

(2) When comparing Predomics and SOTA methods, author provided a normalized coefficient to reflect the interpretability of Predomics' results. But when author used mean decrease accuracy plots to indicate feature, the importance score of SVM and ENET was missed. I wonder the importance score comparison between these SOTA methods and Predomics.

We thank the referee for raising this point. As a reminder, for the BTR models we defined feature importance (FI) as the usefulness of features to be predictive, given all other features and the models in the family of best models (FBM). In short, during each cross-validation fold, the out-of-bag error on each model of the FBM is computed. The importance of the jth feature is measured by permuting all features within the out-of-bag data. The out-of-bag error is computed on this perturbed data for each FBM model. The importance score for the jth feature is obtained by averaging over all FBM models the difference in out-of-bag error before and after the permutation. Finally, the mean decrease accuracy (MDA) is computed as the average of these values over all the folds and is displayed along with the standard error of the mean.

ENET and SVM methods do not provide such information on feature importance, but we used an external R package (rminer v. 1.4.2) to compute the FI of these models. We have compared the FI of BTR models with that of Random Forest, SVM and ENET as well as included another generic approach of FI, which is to rank variables following statistical tests (T-test, Mann Whitney, etc).

Below we provide additional analyses of the FI of the BTR models and compare them with the FI identified using RF, SVM, ENET as well as p-values of Mann-Whitney tests. As depicted in Figure R2, the FI of the three BTR models correlates strongly between the models (bin correlates at 0.76 and 0.83 respectively with ter and ratio, using Pearson correlation). It also correlates strongly with the FI values as quantified by RF (0.68, 0.81, 0.7, respectively with bin, ter and ratio). However, the FI of SVM models is quite different and does not correlate well with the above (-0.04, 0.06, -0.22 and 0.07 respectively with bin, ter, ratio and RF). FI of ENET models correlates better than SVM (0.3, 0.4, 0.15 0.4 and 0.29 respectively with bin, ter, ratio, RF and SVM) and also correlates with SVM itself (0.29). The correlation with the p-value of the statistical tests is negative (a smaller p-value is more significant and its importance is higher). However, these correlations are small as the p-value distribution is not normal and the relation not-linear.

When the correlations are computed for each of the modalities of the "status" variable, we notice that correlations are stronger for the modality: status = -1 (blue points depicting features enriched in the patient group). This indicates again that the most important features are enriched in patients as discussed in the main text and supplementary material of the manuscript~~)~~.

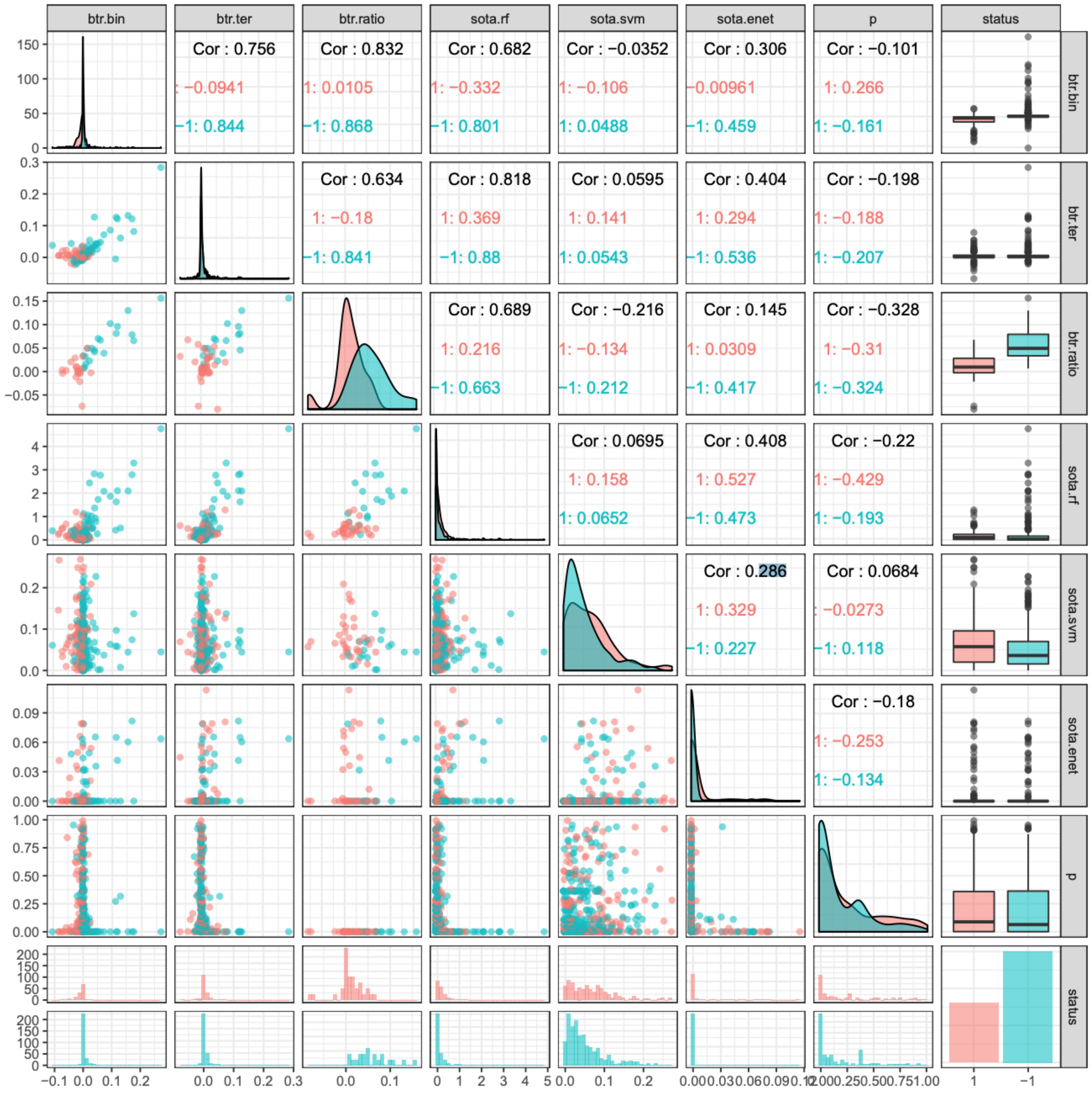

Figure R2: Comparison of feature importance between BTR models and RF as well as statistical ranking. Pairwise comparison of the feature importance identified by different models. Figures are annotated by the enrichment of the variables as -1 (blue) and 1 (red) being respectively more abundant in patients and controls in the Cirrhosis-1 (species) dataset. Besides the histograms, boxplots and density plots are shown.

*Next, we focused on the specific relation of the FI of the ter models vs. RF (see **Figure R3**) to explore the most important features and noticed that they are <u>very similar. The most important features</u> and largely discussed in the article. These analyses show that FI in the BTR models (which are extremely simple) correlates well with that of well-established (but more complex) methods such as RF and at a lesser level with ENET. Nevertheless, SVM seems to be quite different from all the other methods.*

We have included these findings in page 11 of the revised supplementary material.

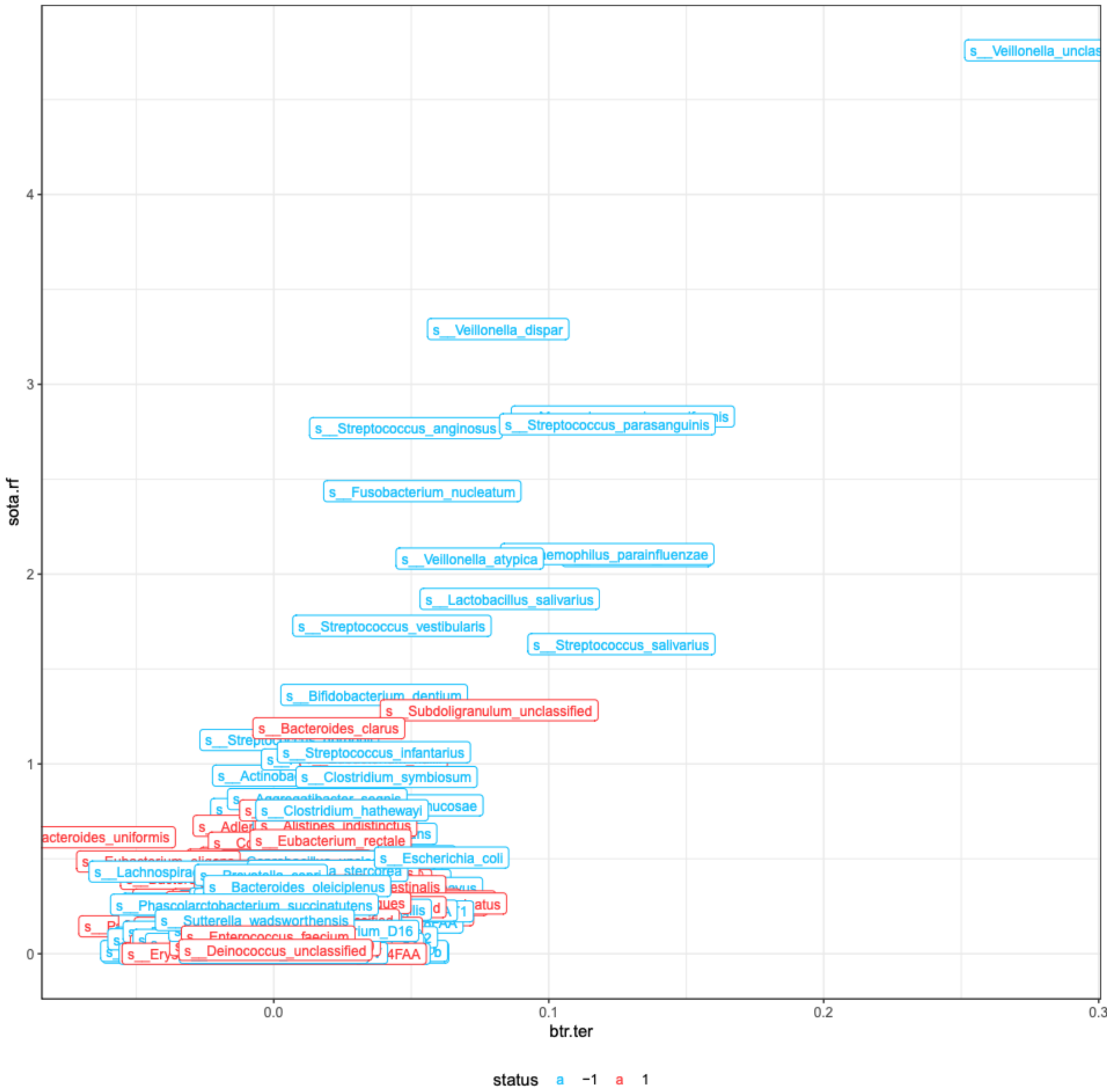

Figure R3: Comparison of feature importance between TER models and RF.
The names of the features are placed according to their feature importance for TER and RF respectively in the x and y axis. Colors (blue and red) indicate enrichment respectively in patients and controls.

We thank the referee for her/his careful reading and constructive remarks, which we hope we have fully addressed.

Reviewer #2

The authors present a well-written manuscript that addresses the potential for explainable machine learning approaches to diagnose disease using metagenomics data. Specifically, the authors present a machine-learning approach that they call "predomics" to search for predictive signatures that are interpretable. This is an important step forward in converting raw metagenomic data into actionable predictive analyses that can inform clinical decision-making. To validate their method, they compare and contrast SOTA algorithms to BTR models using 109 datasets taken from a curated metagenomic dataset (Pasolli et al. 2017) comprised of studies that used shotgun metagenomic sequencing and were analyzed using a consistent pipeline. Datasets included in this analysis are a subset of the curated dataset including gut microbiome studies with varied disease states such as liver cirrhosis, IBD, type 2 diabetes, and obesity-related phenotypes. I commend the authors on their use of a curated dataset (Pasolli et al. 2017), as it helps to align this study with other studies. Further, the consistent annotation makes the data comparable across studies in this analysis. Finally, I think their experiments show that BTR models offer a simplified interpretation compared to the more complex models, which is needed for translating clinical data. The authors are thorough in their analysis and produce compelling results, however, I have a few remaining questions and note a few typos they should correct in the final proof.

We thank the referee for attesting on the importance of the predomics approach in "converting raw metagenomic data into actionable predictive analyses that can inform clinical decision-making" as well as judging the evidence of our experiments compelling.

1. Abstract has two words misspelled, should say "state-of-the-art" and "distill"

We have corrected these typos in the revised version of the manuscript.

2. BTR is introduced in the Methods section on page 4, but the acronym isn't defined until page 6.

We thank the referee for having caught this point. We have also added the definition of BTR models in the methods section.

3. The code is not currently available here: https://git.integromics.fr/published/predomics

This is the git repository of our lab and the project is published and ought to be accessible without restriction. We validated its access from an external computer (without account) as displayed in Figure 4. Moreover, we registered predomics in the Scicrunch.org repository under the accession number SCR_017415.

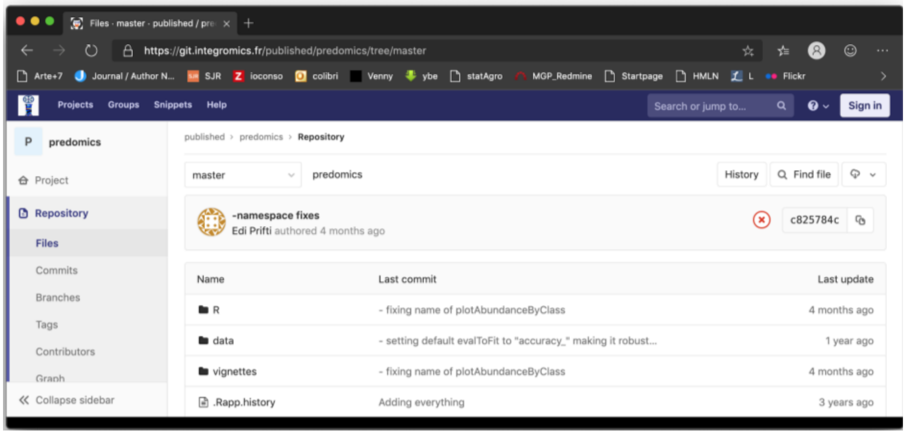

Figure R4: Access of the code in the Integromics git repository.

4. The statement that "*The Virus phylum was not previously identified but is more prevalent and abundant in the liver cirrhosis group, which may reflect the oral microbiome signature and/or reflect an increased incidence of viral infections together with opportunistic pathogens*", needs to be more carefully

considered. First, the original paper that the data are from did not compare against viral reference genomes, only bacteria and archaea (see supplemental Table 2 in Qin et. al). Thus, no viruses were detected simply because they were not used in the analysis. Second, the curated dataset used in this paper from Pasolli et al. uses MetaPhlAn to assign taxonomy. MetaPhlAn uses a genome size correction in the algorithm when assigning organismal abundance, rather than read abundance alone. As a result, small genomes (from viruses and plasmids) can be overamplified in the resulting counts. Meaning that a small number of viral reads can have a big effect on the overall abundance of viruses in the sample, and may have an impact on the ratio of organisms which highly variable genome sizes and thus affect this conclusion. Can the authors provide further clarification of the impact of this algorithmic choice in calculating abundance and their resulting analyses?

We thank the referee for raising this point that we had not considered nor discussed. We agree that the reference data used for quantification plays a pivotal role in the results of the models, and we also agree that in comparison with the original paper, the viral signature could be influenced by differences in reference datasets used. However, this does not invalidate the observation that the Virus phylum prevalence and abundance are higher in the liver cirrhosis group based on the MetaPhlan data. In page 14 of the supplementary material we have revised the statement as follows: The Virus phylum was not previously identified, which could be explained by the reference database used in the original study. However, in the current study, it is observed to be more prevalent and abundant in the liver cirrhosis group.

In relation with the MetaPhlAn quantification, the correction for feature abundance calculation is not strictly based on the real genome size but on the size of the marker genes used for feature quantification, which according to the reference MetaPhlan2 publication (where the quantification of viruses were introduced) shows similar average lengths as bacterial and archaeal marker genes (Table S2 of https://www.nature.com/articles/nmeth.3589). Additional analyses (data not shown) indicate that this difference in marker gene length is about 7 times higher for bacteria. The impact over the results would be lower than if this same correction had been carried out over quantifications based on read mapping over entire genomes (estimated to be on average > 80-times larger in size).

More generally, many issues with the quantification of metagenomic features, which could have a major impact over the sign and significance of the associations found in quantitative metagenomic studies and as a consequence also over predictive models based on these data, are yet to be solved. Recent work, focused on a normalization approach based on external variables that measure microbial density as the number of cells per sample has shown significant impact in the subsequent results and interpretation (Vandeputte et- al Nature 2017 DOI: 10.1038/nature24460).

However, these issues are outside the scope of this paper. The main objective of this work is to propose a novel method, which allows identifying simple models that improve interpretability as well as generalization. We did not compute the abundance of the data but rather used a curated dataset (Pasolli et al. 2017), as mentioned and commended above by the referee. If the abundance table have misquantified values, they will be inevitably propagated down to the analyses end eventually captured by the models. However, the ratio models that we have devised are more robust to such computation errors and biases in quantification, as discussed in the main text of the manuscript.

5. Recently, shallow whole genome shotgun sequencing has been proposed as a more cost-effective approach to provide more accurate taxonomic information (compared to 16S rRNA) for clinical use. Can the authors comment on how shallow WGS sequencing might affect their algorithm and interpretation of results? Further, sequencing experiments have a varied depth of coverage, and therefore organisms/functional traits that are rarer might not be detected with shallow sequencing. Because the authors are focused on clinical interpretations and guidance for clinicians and patients, a discussion of these points is important and relevant in this manuscript.

We thank the referee for having sparked this important discussion. We are aware of the issues that shallow sequencing can have on metagenomics data, especially for low abundant taxa. In the context of this work, this is particularly important as rare features can be found in the models, which would make them generalize badly. For these reasons we have implemented several filtering approaches based on prevalence etc. (discussed in the package tutorials) that would reduce the number of features to be combined in the models. Moreover, the notion of sparsity through a penalization approach will force only several important features to be used - lowering the risk of rare features to be included in the models. We have added the following phrases in the discussion section of the revised manuscript (page 12): "Moreover, varying sequencing depth, can be an issue in over or under estimating less abundant taxa that can find themselves in the models. It is thus advisable to pre-filter rare taxa from the dataset, before training the models. However, the sparsity constraint, will force important taxa to be selected, improving the generalization of the models."

In a clinical setting, once the models are trained and the best features are selected, only these corresponding taxa would need to be quantified and this could be achieved through sequencing or other wet lab approaches. More generally, the sequencing depth should be dependent on the complexity of the ecosystem. A rich microbiome could have several times more taxa than a poor one and would need several times more sequencing depth for a more realistic comparison of the abundances of the taxa throughout the samples. Shallow sequencing could be more suited for low-complexity ecosystems like lung or vaginal microbiome rather than more complex ones such as the gut microbiome. However, with sequencing costs decreasing exponentially, sequencing depth shouldn't be a long-term issue for clinical microbiome studies. We believe that deep sequencing will be more beneficial since it would allow for more in-depth profiling of the ecosystems (e.g. strain profiling), which again increases the space of features over which our algorithm could be applied.

The debate on 16S rRNA vs. whole shotgun (shallow or deep) is still open in the field and depends on the applications. From the results presented here we can notice that in conditions when the signature is strong, shallow sequencing will suffice to produce good models (see ***Figure 3B*** *of the manuscript). The accuracy decreases with the decreased resolution of taxonomic levels. However, the genus level, which is the closest to OTUs identified with 16S rRNA, seems to be sufficient. Our approach is thus applicable also to 16S rRNA datasets and we have already applied it to such datasets (unpublished data).*

6. Have the authors considered the runtime for their method to produce clinically relevant data and results? The method requires extensive annotation including genes, pathways, taxonomic annotation, and then curation and association analysis based on co-occurrence networks and functional analysis (e.g whether or not organisms are butyrate producers). Would the algorithm be better suited for biomarker discovery and validation, where biomarkers are then used in the microbiology lab via simple qPCR-based tests? Logistically, this approach might be best suited for less-time sensitive tasks. If so, the authors should note this.

We thank the referee for the suggestion. Concerning the runtime of our method, which revolves around the identification of predictive models, the genetic algorithm is a powerful approach in exploring in a very short time very large combinatorial spaces. Typically, the algorithm will take less than one minute to find the best features and construct a BTR model. The runtime of the constitution of the training datasets is not discussed as is out of the scope of this work. Once the models are identified, the quantification of the features used to predict can be adapted to a clinical setting as discussed above, including qPCR. However, we believe that new technologies such as ion-based sequencing could be better adapted in a clinical setting as they would allow rapidly quantifying features for a multitude of microbiome-based tests. We have adapted the discussion of the revised manuscript (page 13), by including the qPCR-based tests : "Finally, besides

quantifying taxa abundance through whole shotgun of 16S rRNA sequencing, BTR models can be used to develop specific acquisition technologies such as microarray DNA chips or qPCR-based tests, built with primers that are specific to the species/taxa found in the models [46].”

7. Please convert all instances of "16S" to "16S rRNA", as in "16S surveys" on page 11

We have corrected the inconsistencies in 16S rRNA naming.

8. The authors use rich WGS datasets that have multiple features that can be used in training the algorithm such as taxonomic abundance from all domains of life (bacteria, archaea, fungi) and viruses, metabolic profiles, and curated patient metadata. But, suggest that species-level abundances are a key feature of their approach in defining ecosystem-level interactions for prediction. Can this approach be used with 16S rRNA data alone? Have the authors tested this? Given issues with human contamination in the vast number of clinical samples, the utility of this approach on 16S rRNA datasets would be of interest to the community and readers.

It is true that we have not specifically discussed Predomics results with 16S rRNA datasets because the reference datasets we have chosen for evaluation (available in curatedMetagenomicData repository) include quantification at different taxonomic levels. Our approach can be applied with 16S rRNA data in a similar way. As discussed above, we have applied it to unpublished data and the approach works quite well.

In terms of resolution, it is true that 16S rRNA data does not yield the same results as shotgun sequencing. However, approaches like oligotyping (Meren et-al 2015 ISME J. doi: 10.1038/ismej.2014.195.) or algorithms like DADA2 (Callahan et-al 2016, Nat. Methods doi: 10.1038/nmeth.3869.) where Amplicon Sequence Variants (ASV) are defined, allow more specific typing of microbial communities than through standard OTUs. These approaches could be used to define the feature space for model prediction, potentially improving the resolution of predicted models.

In terms of human contamination, we assume the reviewer refers to the potential amplification of mitochondrial sequences in 16S rRNA amplicon studies. We agree that this is a major problem in microbiome studies, but this should be addressed through dedicated methods for sequence filtering and denoising of 16S rRNA datasets before generating the final quantitative data to use for model prediction.

Overall, really nicely written paper, and careful analyses that is worthy of publication.

We thank the referee for her/his careful reading and constructive remarks, which we hope we have fully addressed.
