# Supplementary material for: Interpretable and accurate prediction models for metagenomics data
Source: Gigascience. 2020 Mar 9;9(3):giaa010. doi: 10.1093/gigascience/giaa010 (PMC7062144; doi:10.1093/gigascience/giaa010)
Supplement: giaa010_GIGA-D-19-00177_Revision_2 [file giaa010_giga-d-19-00177_revision_2.pdf]

GigaScience

Interpretable and accurate prediction models for metagenomics data

--Manuscript Draft--

**Manuscript Number:**	GIGA-D-19-00177R2
**Full Title:**	Interpretable and accurate prediction models for metagenomics data
**Article Type:**	Research
**Funding Information:**	Assistance Publique-Hôpitaux de Paris (Contrat d'interface chercheurs 2015-2018) — Professor Jean-Daniel ZUCKER<br>French National Agency through the national program Investissements d'Avenir IHU ICAN (ANR-10-IAHU-05) — Dr Edi Prifti<br>Funding Support of European Union's Seventh Framework Program (HEALTH-F4-2012-305312) — Prof. Karine Clément
**Abstract:**	Background: Microbiome biomarker discovery for patient diagnosis, prognosis and risk evaluation is attracting broad interest. Selected groups of microbial features provide signatures that characterize host disease states such as cancer or cardio-metabolic diseases. Yet, the current predictive models stemming from machine learning still behave as black boxes and seldom generalize well. Their interpretation is challenging for MDs and biologists, which makes them difficult to trust and use routinely in the physician–patient decision-making process. Novel methods that provide interpretability and biological insight are needed. Here, we introduce "predomics", an original machine learning approach, which is tailored for metagenomics data. It discovers not only accurate predictive signatures, but also provide unprecedented interpretability. Inspired by microbial ecosystem interactions, the decision provided by the predictive model is based on a simple, yet powerful score computed by adding, subtracting or dividing cumulative abundance of microbiome measurements. Results: Tested on more than 100 datasets, we demonstrate that predomics models are extremely simple and thus highly interpretable. Even with such simplicity, they are as accurate as state-of-the-art methods. Moreover, the family of best models, discovered during the learning process, has the ability to distill biological information and help decipher the predictability signatures of the studied condition. Finally, in a proof-of-concept experiment, we successfully predicted body corpulence and metabolic improvement after surgery using pre-bariatric surgery microbiome data. Conclusions: Collectively, this approach builds up both reliable and trustworthy diagnostic decisions while agreeing with societal and legal pressure that require explainable AI in the medical field.
**Corresponding Author:**	Jean-Daniel ZUCKER<br>Institut de recherche pour le développement France-Nord<br>BONDY CEDEX, Île-de-France FRANCE
**Corresponding Author Secondary Information:**	
**Corresponding Author's Institution:**	Institut de recherche pour le développement France-Nord
**Corresponding Author's Secondary Institution:**	
**First Author:**	Edi Prifti, PhD
**First Author Secondary Information:**	
**Order of Authors:**	Edi Prifti, PhD<br>Yann Chevaleyre, PhD<br>Blaise Hanczar, PhD<br>Eugeni Belda, PhD<br>Antoine Danchin, PhD

Powered by Editorial Manager® and ProduXion Manager® from Aries Systems Corporation

	Karine Clément, PhD
	Jean-Daniel ZUCKER
**Order of Authors Secondary Information:**	
**Response to Reviewers:**	Please find all answers to reviewers (which include graphics) as well as letter to editors in the attached document named Predomics_Cover_letter_And_Response_To_Reviewers_2_review.
**Additional Information:**	
**Question**	**Response**
Are you submitting this manuscript to a special series or article collection?	No
**Experimental design and statistics**<br><br>Full details of the experimental design and statistical methods used should be given in the Methods section, as detailed in our Minimum Standards Reporting Checklist. Information essential to interpreting the data presented should be made available in the figure legends.<br><br>Have you included all the information requested in your manuscript?	Yes
**Resources**<br><br>A description of all resources used, including antibodies, cell lines, animals and software tools, with enough information to allow them to be uniquely identified, should be included in the Methods section. Authors are strongly encouraged to cite Research Resource Identifiers (RRIDs) for antibodies, model organisms and tools, where possible.<br><br>Have you included the information requested as detailed in our Minimum Standards Reporting Checklist?	Yes
**Availability of data and materials**<br><br>All datasets and code on which the conclusions of the paper rely must be either included in your submission or	Yes

Powered by Editorial Manager® and ProduXion Manager® from Aries Systems Corporation

deposited in publicly available repositories (where available and ethically appropriate), referencing such data using a unique identifier in the references and in the "Availability of Data and Materials" section of your manuscript.<br><br>Have you have met the above requirement as detailed in our Minimum Standards Reporting Checklist?	

Powered by Editorial Manager® and ProduXion Manager® from Aries Systems Corporation

Manuscript

Click here to view linked References

Click here to access/download;Manuscript;Predomics_main_text_figures_on

Interpretable and accurate prediction models for metagenomics data

Authors / affiliations :

Edi Prifti *[,1,2], Yann Chevaleyre [3], Blaise Hanczar [4], Eugeni Belda [1], Antoine Danchin [5], Karine Clément [6,7], Jean-Daniel Zucker *[1,2,6]

[1] IRD, Sorbonne University, UMMISCO, F-93143, Bondy, France

[2] Institute of Cardiometabolism and Nutrition, ICAN, Integromics, Paris, France

[3] Paris-Dauphine University, PSL Research University, CNRS, UMR 7243, LAMSADE, Paris, France

[4] IBISC, University Paris-Saclay, University Evry, Evry, France

[5] Institut Cochin INSERM U1016 - CNRS UMR8104 - Université Paris Descartes, Paris, France

[6] Sorbonne University, INSERM, Nutrition and Obesities; systemic approach research unit (NutriOmics), Paris, France

[7] Assistance Publique-Hôpitaux de Paris, Nutrition department, CRNH Ile de France, Pitié-Salpêtrière Hospital, Paris, France

Contact information*:*

Correspondence: Edi PRIFTI (edi.prifti@ird.fr) and *Jean-Daniel ZUCKER (jean-daniel.zucker@ird.fr)*[1] *; 50/52, bd Vincent Auriol, 75013, Paris, France*

Declaration of interest: none to declare

[1] Lead author

Abstract

Background: Microbiome biomarker discovery for patient diagnosis, prognosis and risk evaluation is attracting broad interest. Selected groups of microbial features provide signatures that characterize host disease states such as cancer or cardio-metabolic diseases. Yet, the current predictive models stemming from machine learning still behave as black boxes and seldom generalize well. Their interpretation is challenging for MDs and biologists, which makes them difficult to trust and use routinely in the physician–patient decision-making process. Novel methods that provide interpretability and biological insight are needed. Here, we introduce "*predomics*", an original machine learning approach, which is tailored for metagenomics data. It discovers not only accurate predictive signatures, but also provides unprecedented interpretability. Inspired by microbial ecosystem interactions, the decision provided by the predictive model is based on a simple, yet powerful score computed by adding, subtracting or dividing cumulative abundance of microbiome measurements.

Results: Tested on more than 100 datasets, we demonstrate that predomics models are extremely simple and thus highly interpretable. Even with such simplicity, they are as accurate as state-of-the-art methods. Moreover, the family of best models, discovered during the learning process, has the ability to distill biological information and help decipher the predictability signatures of the studied condition. Finally, in a proof-of-concept experiment, we successfully predicted body corpulence and metabolic improvement after surgery using pre-bariatric surgery microbiome data.

Conclusions: Collectively, this approach builds up both reliable and trustworthy diagnostic decisions while agreeing with societal and legal pressure that require explainable AI in the medical field.

Keywords:

prediction, interpretable models, metagenomics biomarkers, microbial ecosystems

Background

An increasing wealth of data from high-throughput molecular and imaging technologies is connecting biomedical sciences and machine learning (ML). The latter is impacting numerous areas of medicine, including disease diagnosis and prognosis [1-3]. It is now argued that ML and more globally artificial intelligence (AI), will dramatically improve prognosis within the coming years [4].

Simultaneously, progress made in high throughput technologies has contributed to developing new fields such as metagenomics. The association of the gut microbiota with human health and disease has been widely discussed [5] and links with numerous diseases are described [6-13]. Specifically, ecological relationships among bacterial species such as mutualism, parasitism, and competition [14] may change along with a shift in microbial equilibrium. Although these signatures allow predicting diseases, many of these findings are only correlative and require controlling for confounding factors — a task that remains challenging [15].

Metagenomics data must be interpreted carefully as they are often analysed in a small number of samples (N) compared to a very large number of variables (p). Current microbial catalogues, which are composed of millions of genes [16] and thousands of bacterial species and functional profiles [17], allow characterizing and comparing sampled ecosystems. Consequently, most models tend to overfit the training data and result in predictions arising from random sampling fluctuations. To reduce overfitting and allow for better generalization in unseen data, some authors use learning algorithms that include a dimension reduction or regularization methods, e.g. Elastic Net [11] or SVM-RFE [15]. While these algorithms are more straightforward than others, they generate complex models that are difficult to interpret. ML research has focused on building accurate models for large data collections, often at the expense of interpretability.

Providing an *explanation* of the prediction process is increasingly requested [18] when not mandatory [19], especially in precision medicine [18, 19]. *Interpretable* models have two desirable properties: conciseness and readability by non-experts. They should contain simple operations and be limited in size [20-22]

Causality, as the holy grail of modern biology, is out of the scope of the interpretability property of a predictive model. Here, we investigated if models inspired by ecosystem relationships and sparse microbial signatures can be both accurate and more interpretable than more complex

well established state-of-the-art (i.e. SOTA) models, including logistic regression with elastic-net regularization (ENET) and support vector machines (SVM).

Data Description

We used public datasets to test our proposed algorithm *predomics* and compare it with SOTA methods. For the classification tasks we used curated metagenomic datasets from ExperimentHub [23] (see supplementary material; **Table S1**). The code used to query and process the data is provided in the supplementary material package. In total, 54 datasets were derived (i.e. six different cohorts and for each six taxonomic levels, a marker gene and a pathway table along with a fused taxonomic dataset). They were also transformed as presence/absence for additional experiments (n=54).

Baseline microbiome data were also used to predict the clinical outcome of bariatric surgery on morbidly obese patients [24]. Their microbiome was sequenced at baseline, one, three, and twelve months after surgery (see original paper for methods).

Methods

The *predomics* optimization algorithm

Here, we propose a new family of models, named BTR for Binary/Ternary/Ratio. Learning optimal BTR models is computationally difficult. Because weights are discrete, usual techniques coming from convex optimization do not apply. A naïve way would be to perform an exhaustive search through the whole space of models. Unfortunately, this is not practically feasible since the computation time would increase exponentially with the number of features. The BTR learning problem is known as NP-Hard, which means that *no* algorithm can solve this problem exactly in polynomial time [25].

We can nevertheless apply heuristics that provides good models without guarantee on their optimality. Genetic algorithm (*i.e.* GA) is a stochastic optimization technique that can be of great use in such context. It adopts concepts from evolutionary biology — populations, reproduction, mutation and generations. The outline of the algorithm is described in the supplementary materials section. After the evolution process, a final population of predictive models is provided. The best model is obtained by applying a so-called *model-size penalization* ($accuracy_{penalized} = accuracy - \lambda k$), where k is the number of features in the model (i.e. parsimony) and λ is an hyperparameter controlling the penalization of the accuracy. Here, we

used ƛ=1%, which means than a model that is using one additional feature will only be preferred if it improves the accuracy of more than 1%.

For classification, *predomics* may be set to optimize different parameters such as the accuracy (default), AUC, F1, precision or recall, while for regression it can optimize R^2 (default), rho or the standard error of the regression.

Experimental design

The experimental pipeline proceeds as follows:

1. Feature normalization: frequency tables are used as processed by Pasolli et al [23].
2. Features with low standard deviation are filtered out. The threshold corresponds to the maximum second derivative of the distribution of the feature's standard deviation.
3. The generalised performance of each method is estimated by 10-times 10-fold cross validation for the classification tasks and a 20-times 5-fold cross validation for the regression tasks.
4. The feature selection is embedded for the BTR models and Elastic Net. For SVM and RF, feature selection is based on the Mann-Whitney score as introduced in [26].
5. Algorithm performances are compared with a paired t-test using the 100 CV estimations. Those that are not significantly different (pval<0.05) are considered equivalent.

The BTR models are tested on 109 different datasets (see **Table S1**) and compared with the methods from the SOTA algorithms: support vector machine (SVM) with linear and Gaussian kernel (data not shown), Random Forest and Elastic Net (an improvement of Lasso, alpha=0.5). All algorithms were evaluated by measuring test accuracy in a cross-validation setting and compared among them using paired t-tests. A specific comparison between TerLog models (i.e. *ter* model with log-transformed data) and the geometric mean balance algorithm is provided in supplementary material.

Family of best models

A family of best models (i.e. FBM) is defined as the set of models returned by the algorithm, whose accuracy is within a statistically equivalent window, defined by a threshold assuming a binomial distribution ($p<0.05$). A FBM can be analysed in detail to distil biological information in the predictive context (see supplementary material).

Feature importance

Similar to RF, feature importance is defined as the usefulness of features to be predictive, given all other features and best models of the FBM. During each cross-validation fold, the out-of-bag error on each model of the FBM is computed. The importance of the jth feature is measured by permuting all features within the out-of-bag data. The out-of-bag error is computed on this perturbed data for each FBM model. The overall *feature importance* for the jth feature is obtained by averaging over all FBM models the difference in out-of-bag error before and after the permutation. This is performed on all the features of the dataset that are found in the FBM models, on which errors before and after permutations are computed. Finally, the mean decrease accuracy (MDA) is computed as the average of these values over all the folds and is displayed along with the standard error of the mean.

Threshold optimization

The threshold used in the model is optimized to maximize the performance in the training set. This approach consists in computing the model's score for each observation in the training set. The observations are ordered based on this score. Next, the cumulative error is computed following the same order - each time an example is misclassified the cumulative error is increased when sliding through the score. The index example on which the cumulative error minimizes will provide the value of the score, which will be used as a threshold in the final model.

Regression models

Predomics can learn regression models, which are evaluated by maximizing either Spearman rho or Spearman R^2 or minimizing the scaled standard error of regression (SER). The model's score at this stage reflects the cumulative/difference/ratio of relative abundance of the species and needs to be scaled in the range of the variable to predict. Two additional parameters alpha (i.e. multiplication factor) and beta (i.e. intercept) are estimated.

Network reconstruction

We used Scalenet [27] to reconstruct the feature co-presence network in model selection data. Here we used the top 5% strongest edges inferred by *bayes_hc* and *aracne* methods in the FBM-presence table. ScaleNet first reduces the reconstruction problem into a number of simpler reconstruction problems, then employs state-of-the-art reconstruction methods to solve

them. Finally, a consensual voting strategy between the methods is adopted to identify accurate sub-graphs, which are then overlapped together.

Results

A new family of models for metagenomics data

We propose a new family of models, named BTR for Binary/Ternary/Ratio, which are a simplification of linear models aiming at making their output even more interpretable. For each ecosystem $y_1 \dots y_n$, the abundance or presence of either genes, taxonomy levels, functions, or other microbial qualities, are represented by $X_1 \dots X_p$ predictor variables. In a linear model, a patient is predicted in a disease group with a probability of $p > \frac{1}{2}$ if $\beta_0 + \sum_{j=1}^{p} \beta_j X_j > 0$, where $\beta_0 \dots \beta_p \in R$ are real coefficients. The biological assumption is that the contribution of each bacterial species to the prediction is proportional to its abundance and that only a limited number of species is sufficient to support the prediction. BTR models are much simpler and are inspired by three hypotheses emphasizing relationships between species and associated ecosystem (**Figure 1**).

Hypothesis 1: The unweighted cumulative abundance of a group of species can predict disease state. We define the *binary models* (*i.e.* Bin) as linear models with the additional constraint that each coefficient $\beta_1 \dots \beta_p$ (omitting the intercept β_0) must be binary — {0, 1} (**Figure 4A**). An example is in *(1)* and is interpreted as "if the cumulated abundance of *s_Veillonella_unclassified* and *s_Lachnospiraceae_bacterium_3_1_57FAA_CT1* is smaller than 9.7% of the total microbial abundance, then the individual is classified as healthy". These species may share the same ecological niche or interact directly with one another [28, 29].

(1) **If** s_Veillonella_unclassified **+** s_Lachnospiraceae_bacterium_3_1_57FAA_CT1 **<** 0.097
then class **=** healthy

Hypothesis 2: The difference of unweighted cumulative abundance of two groups of species can predict disease state. This assumption is implemented by *ternary models* (*i.e.* Ter), also linear models with the constraint that each coefficient $\beta_1 \dots \beta_p$ (omitting the intercept β_0) be limited to the values {-1, 0, 1} (**Figure 4B**). An example of a ternary model in *(2) can be* interpreted as follows: "if the cumulative abundance of *s_Streptococcus_anginosus* and

s_Veillonella_unclassified minus the abundance of *s_Alistipes_indistinctus* is greater or equal than 8.3 % of the total microbial abundance, then the patient is classified as healthy".

(2) **If** (s_Streptococcus_anginosus + s_Veillonella_unclassified) - s_Alistipes_indistinctus ≤ 0.083

then class **=** healthy

<u>Hypothesis 3</u>: The ratio of unweighted cumulative abundance of two groups of species can predict disease state. This assumption is implemented by *ratio models* (*i.e.* Ratio), also linear models with an additional constraint: each coefficient $\beta_1 ... \beta_p$ is limited to a value of -θ, 0 or 1, where θ is a positive real number, and the intercept β_0 is set to zero (**Figure 4C**). An example in *(3)* can be interpreted as follows: "if the abundance of *s__Subdoligranulum_unclassified* is $\theta = 81$ times greater than the cumulative abundance of *s__Megasphaera_micronuciformis* + *s__Streptococcus_anginosus* then the individual is classified as healthy".

(3) **If** $\frac{s\_Subdoligranulum\_unclassified}{s\_Megasphaera\_micronuciformis + s\_Streptococcus\_anginosus} > 81$

then class **=** healthy

Biologically, both Ter and Ratio models can correspond to interactions of different types of species, including cooperation and competition among species. BTR models can be illustrated as balances, where species abundance is symbolized by the cumulative weights (**Figure 1**). The concept of balance is not new in ecology and was first proposed to address the compositionality problem in microbiome data. A balance-based representation can bypass this issue and reveal pertinent biological patterns [30]. Very recently, other authors have applied the balance representation in the classification context [31]. Here, we propose a more general framework of models that encompass such balances. Indeed, they would correspond to our Ter models when applied to log-transformed data — named TerLog (see supplementary material; **Figure S14**). Learning linear models on log-transformed relative abundance data corresponds to identifying balances of multiplicative relationships. However, which characterizes best microbial ecosystems (i.e. multiplicative or additive), remains an open question. We propose here different types of models that could be useful in tackling such questions. The *predomics* algorithm was developed to specifically learn BTR models.

BTR models are sparse, accurate and improve with taxonomic specificity

We tested our approach on six different public metagenomic datasets (**Table S1**) and nine derived types of variables, (six different taxonomic levels, a merged multi-taxonomic level, marker genes and a functional MetaCyc pathway table, *i.e.* a total of 54 datasets, see methods). We trained and tested models with different number of features (*i.e.* model-size, k_#) and noticed an effect on accuracy. As expected, the testing performance on unseen data was lower compared to training performance. However, this difference was more pronounced for the SOTA, indicating a significant overfitting effect, compared with BTR models. The simplicity and sparsity of the BTR models reduces overfitting on studied datasets (**Figure 2**). As BTR models come with an embedded feature-selection strategy, we used a Mann-Whitney test to select the k_# most correlated features for RF and SVM to allow comparison. For ENET we used the embedded regularization path and selected the first k_# form it.

We applied a model-size penalization technique on the empirical (training) accuracy to select the best model. BTR models performed at least as well as the SOTA in 46/54 (85%) of the cases. They outperformed SOTA in 19/54 (35%) and were outperformed in 8/54 (15%) (**Figure 3; Figure S1A-C)**. Similar results were observed even when all the variables in the dataset were used (no-penalization) for the SOTA (**Figure S2A-C**) or when fixing the same model size for all the compared models (**Figure S3A-C**).

When learning from the different types of variables based on taxonomic levels (Cirrhosis stage-1), the performance of the models vary accordingly. Higher performance is obtained at the gene marker, species and genus levels, and decreases with higher taxonomic levels. Moreover, when applied to a multi-taxonomic level dataset (*strain* to *phylum* as generated by Pasolli et al. [23] with different specificity levels mixed together; *i.e. whole tax*), models displayed surprisingly good performance (**Figure 3B**). Indeed, in this space, models can be powerful as they can summarize more complex rules such as: "**if** (abundance of all Firmicutes **–** abundance of all Clostridiales order) **>** threshold **then** disease".

We tested the generalization of Bin, Ter, Ratio and also TerLog models trained in Cirrhosis stage-1, in a second, independent dataset (i.e. Cirrhosis stage-2). Results illustrated in **Figure S5** indicate very good external validation with an average training accuracy=0.89 (sd=0.02) and testing accuracy=0.85 (sd=0.04). Ter and Ratio models generalized better compared to Bin and TerLog (see supplementary material).

In addition to the abundance datasets described above, we trained and tested similar models on *presence/absence* binary data derived from the previous 54 abundance datasets. Overall results are similar indicating that the detection of species alone can be powerful enough in prediction tasks (see supplementary material; **Figure S1D-F; Figure S2D-F; Figure S3D-F; Figure S4**). Noteworthy, when applied to presence data, BTR models indicate relationships between sub-ecosystem complexity or richness. These can be useful to detect switch-like mechanisms in the microbiome.

BTR models generate straightforward interpretations in contrast to state-of-the-art models

A graphical barcode representation illustrates the simplicity of BTR models. In **Figure 4A-C** *left*, the models are represented by red and blue horizontal lines, corresponding respectively to positive and negative coefficients (either 1 or -1). The same representation is used to visualise the normalized coefficients of ENET and SVMLIN models (the line length is proportional to the coefficient in the interval [-1,1]) (**Figure 4D-E**). For the RF model only one of the 500 decision-trees used in the model is illustrated (**Figure 4F**). Additionally, for each variable selected by BTR models, we assessed their importance in prediction, using a variant of the well-known mean decrease accuracy (MDA) (**Figure 4A-C** *middle*). The feature importance (FI) score of BTR models correlates strongly with the FI of the well-established but more complex RF model (respectively R=0.68, R=0.81, R=0.7, with bin, ter and ratio models; **Figure S11, S12**). This information allows prioritizing further exploration of the features in the context of the predicted phenomenon.

Predomics generates a family of BTR models with equivalent predictive power in a given model-size range (*i.e.* FBM for family of best models; **Figure S8**; see methods and supplementary material). FBM is analysed to identify the common features that are found in the models. For instance, in the cirrhosis stage-1 (species) dataset, the 268 models in the FBM with model-size<6 rely only on 67 features (i.e. 16% of the whole dataset), which can be used to infer the *feature co-presence network in models* (**Figure 5A**). An emerging property of this network is the clustering of phylogenetically related species, such as Firmicutes species enriched in patients (blue tones) and Proteobacteria and Actinobacteria enriched in controls (green tones). Co-presence of the features indicate complementarity in prediction (red edges), while replacement of the features by one another indicate redundancy (blue edges). This can also be observed with the inverse relation of feature pairs in the data and in the models (**Figure**

5C) — the most correlated pairs in the dataset are those that do not occur together in the models. This network provides precious information to decipher the sub-ecosystem that is the most associated with the disease (**Figure S8-S10**).

BTR models provide biological insights

We focused on the liver cirrhosis dataset [7], where major patient dysbiosis was observed with decreased microbial richness, depletion of gut commensals, and an invasion of oral bacteria. Several markers at taxonomic and functional levels were associated with the disease.

Some authors have modelled liver cirrhosis associated microbiome using curated information from the literature, such as the ratio of autochthonous (butyrate-producer bacteria) to non-autochthonous (oral bacteria, opportunistic pathogens). They used these taxa to build a cirrhosis dysbiosis ratio (CDR) score [32]. Based on their description we built three redundant ratio models using family taxonomic features to reproduce their score and applied them in the liver cirrhosis stage 1 (family) dataset [7] (**Figure 6B-D**). We searched the same family-level dataset for Ratio models, which provided superior performance (accuracy=0.86; **Figure 6A**) compared with CDR-based models (accuracy=0.56 in average; **Figure 6F**). The reason for the CDR lower performance can be explained by the inclusion of the *Bacteroidaceae* family in the liver cirrhosis group by the authors, while we observe the opposite association in the current dataset. *Bacteroidetes*-related features are enriched in the control group and this is consistent for different taxonomic levels (**Figure 6E**, see supplementary material).

At the phylum level, the Ratio model *(S6)* points at a mutual exclusion between Bacteroidetes and the combination of Proteobacteria and Viruses, which is also picked up by the Bin model *(S4)*. These models are in line with a decrease in Bacteroides and an increase in Proteobacteria and Fusobacteria in the liver cirrhosis group, reported in the original study. The decrease in Bacteroidetes indicates a decrease in highly prevalent gut bacteria, whereas the increase of Proteobacteria has been repeatedly reported in dysbiotic microbiomes of patients and has been associated with chronic inflammation and serum lipopolysaccharides [33, 34]. The Virus prevalence in the liver cirrhosis group, may reflect the oral microbiome signature or increased incidence of viral infections together with opportunistic pathogens.

The potential competition between oral and gut microbes reported in previous studies [35] is best reflected by Ter and Ratio models with genus abundance data, that combine *Veillonella* (oral bacteria; opportunistic pathogen) enriched in liver cirrhosis at one side and *Bacteroides*

plus *Eubacterium (S9)* or *Coprococcus (S8)* enriched in controls. The latter represent butyrate producers (*Coprococcus* and *Eubacterium*) and complex polysaccharide degraders (*Bacteroides* genus) [36]. Among the most important genera in the FBM we find *g__Veillonella*, *g__Streptococcus*, *g__Haemophilus*, *g__Coprococcus* and *g__Lactobacillus*, all more abundant/prevalent in patients.

Best Ratio and Ter models *(1-3)*, include oral bacterial species of the genus *Veillonella (Veillonella_unclassified), Streptococcus (S. parasanguinis and S. anginosus)* and opportunistic pathogens like *Megasphaera micronuciformis* that proliferate in liver cirrhosis patients, whereas butyrate producers of the genus *Subdoligranilum* (*Subdoligranilum unclassified*) closely related to *Faecalibacterium prausnitzii [37]* and complex polysaccharides degrading species like *Bacteroides cellulosilyticus [38]* characterize control subjects. *Megasphaera micronuciformis* was previously associated with primary biliary cirrhosis based on 16S rRNA quantification [39]. A more in-depth exploration of the FBM (**Figure S8-S10**) and the feature-model co-occurrence network (**Figure 5**) delineates detailed relations of the predictive sub-ecosystem.

At the functional level, predictive models *(S10-S12)* from MetaCyc pathway abundance data include pathways that suggest an increased aerobic metabolism (HEMESYN2−PWY: heme biosynthesis II (anaerobic), essential for cytochromes and heme-containing globins, PWY−922: mevalonate pathway I, needed for the biosynthesis of ubiquinone and menaquinone complexes of respiratory chains). Interestingly, increase in aerobic respiration profiles has also been identified as metabolic signatures of inflammation-associated dysbiosis in models of colitis [40]. Moreover, we observe the presence of modules related with bacterial peptidoglycan biosynthesis in the FBM (PWY−6470: peptidoglycan biosynthesis V). It has been described as an elicitor of inflammatory response associated to the progression of liver cirrhosis [41], in agreement with a more inflammatory profile of cirrhotic patients.

Altogether, these results indicate that BTR models discover important features with relevant biological information. BTR models are more accurate then literature-based ones and have the ability to distil and capture the predictive biological information embedded in the data.

Discussion

In principle, BTR models could be applied to any type of data. However, they are best suited to *commensurable* measurements (*i.e.* variables measurable by the same standard or measure).

In the growing field of metagenomics, issues related to compositionality and data processing still remain to be solved. Recent work has shown the importance of data acquisition in subsequent analytical inferences. In particular, microbial loads differ significantly between individuals and are associated with specific types of microbial ecosystems [42]. An advantage of the *Ratio* models is that they are scale-invariant given they do not depend on absolute measurements, thus avoiding compositionality issues. Moreover, varying sequencing depth, can be an issue in over or under estimating less abundant taxa that can find themselves in the models. It is thus advisable to pre-filter rare taxa from the dataset, before training the models. However, the sparsity constraint, will force important taxa to be selected, improving the generalization of the models.

The simplicity of a BTR model may come with the risk of over-interpretation. The existence of *k* species in a model, may correspond to different explanations ranging from simple correlation to causal relation. They may or may not interact together, as in the case of a niche differentiation [31]. For instance, the buccal-originated species found in the gut of liver cirrhosis patients [7] along with the absence of commensals may reflect a global difference in the environment where they live rather than direct interaction [7]. Even if BTR models represent real interactions between species, it is not recommended to give a causal interpretation without experimental validation. Nevertheless, identifying such species provides important knowledge towards understanding potential mechanisms between species or between species and the host.

The quality of reference datasets used in building predictive models is crucial for model interpretability. The propagation of errors and inaccuracies in genomic datasets is a well-known issue, and affects automated methods for functional annotation [43]. Moreover, due to the lack of biochemical characterization of orphan enzymatic activities, the number of sequences with unknown functions is extremely large, making error percolation of common feature [44] (see supplementary material).

One important issue with microbiome studies resides in the potential confounders modulating microbial ecosystems. For instance, it has been shown that metformin can alter the bacterial ecosystem such that some bacterial species (*e.g. E. coli*) are increased in abundance while others are depleted [45]. It is thus important to filter out confounder-related species from the data or to filter out models that are sensitive to confounders.

Finally, besides quantifying taxa abundance through whole shotgun or 16S rRNA sequencing, BTR models can be used to develop specific acquisition technologies such as microarray DNA chips or qPCR-based tests, built with primers that are specific to the species/taxa found in the models [46]. From a clinical perspective, because BTR models rely on a small number of variables, quantifying a relatively small subset of variables (genes, species, pathways, OTUs, etc) can be sufficient to simultaneously predict multiple tasks. Such applications, after being properly validated, will be important to the medical community in their translational quest in improving patient care. Our approach brings us a step closer towards useful clinical predictions while preserving interpretability.

Potential implications

In our article, we propose an original Machine Learning method, called *predomics*, which is tailored for metagenomics data but can be applied to other fields as well. We strongly believe that this original approach will have significant impact on both the development of predictive models based on metagenomics data as well as their applications to medical conditions. This approach will support clinical decisions in the context of precision medicine. The interpretability of the models will ease acceptability and suggest candidates for microbiome targeted treatments. Moreover, it will serve as a bridge to further develop cross-fertilization between AI, biology and precision medicine.

Availability of source code and requirements

Supplementary information and source data files are available online and the *predomics* package in *https://git.integromics.fr/published/predomics*. The software is registered in the scicrunch.org repository under the RRID: SCR_017415.

Financial support:

This work was also supported by the French National Agency through the national program Investissements d'Avenir (reference no. ANR-10-IAHU-05) IHU ICAN; by the Funding Support of European Union's Seventh Framework Program under grant agreement HEALTH-F4-2012-305312, by the Assistance Publique-Hôpitaux de Paris promoter of the clinic program and by Assistance Publique-Hôpitaux de Paris Contrat d'interface chercheurs 2015-2018. We wish to thank S.D. Ehrlich as well E. Le Chatelier for mindful discussions on the early stages of this work and T. Swartz for help in language proofreading.

Authors contributions:

EP: overall conception, design and interpretation; designing and coding the software; conducting all experiments; writing the manuscript. **YC** conception and interpretation of the approach; coding; drafting the manuscript. **BH**: conception, design and interpretation of the approach; coding; drafting the manuscript. **EB**: biological interpretation of the results; drafting the manuscript. **AD**: biological interpretation of the results; drafting the manuscript. **KC**: data production (bariatric model); biological interpretation of results. **JDZ**: conception, early prototyping, design and interpretation of results; drafting the manuscript. All authors approved the manuscript.

REFERENCES

References

1. Buch, V.H., I. Ahmed, and M. Maruthappu, *Artificial intelligence in medicine: current trends and future possibilities.* The British journal of general practice : the journal of the Royal College of General Practitioners, 2018. **68**(668): p. 143-144.
2. Krittanawong, C., et al., *Artificial Intelligence in Precision Cardiovascular Medicine.* Journal of the American College of Cardiology, 2017. **69**(21): p. 2657-2664.
3. Reynolds, R.J. and S.M. Day, *The growing role of machine learning and artificial intelligence in developmental medicine.* Developmental Medicine Child Neurology, 2018. **59**(Suppl. 3): p. 36-1.
4. Obermeyer, Z. and E.J. Emanuel, *Predicting the Future — Big Data, Machine Learning, and Clinical Medicine.* New England Journal of Medicine, 2016. **375**(13): p. 1216-1219.
5. Walsh, C.J., et al., *Beneficial modulation of the gut microbiota.* FEBS Letters, 2014. **588**(22): p. 4120-4130.
6. Ley, R.E., et al., *Obesity alters gut microbial ecology.* Proceedings of the National Academy of Sciences, 2005. **102**(31): p. 11070.
7. Qin, N., et al., *Alterations of the human gut microbiome in liver cirrhosis.* Nature, 2014. **513**: p. 59.
8. Wen, L., et al., *Innate immunity and intestinal microbiota in the development of Type 1 diabetes.* Nature, 2008. **455**(7216): p. 1109-1113.
9. Qin, J., et al., *A metagenome-wide association study of gut microbiota in type 2 diabetes.* Nature, 2012. **490**: p. 55.
10. Elson, C.O. and Y. Cong, *Host-microbiota interactions in inflammatory bowel disease.* Gut Microbes, 2014. **3**(4): p. 332-344.
11. Zeller, G., et al., *Potential of fecal microbiota for early-stage detection of colorectal cancer.* Molecular Systems Biology, 2014. **10**(11): p. 766-766.
12. Le Chatelier, E., et al., *Richness of human gut microbiome correlates with metabolic markers.* Nature, 2013. **500**(7464): p. 541-546.
13. Cotillard, A., et al., *Dietary intervention impact on gut microbial gene richness.* Nature, 2013. **500**(7464): p. 585-588.
14. Faust, K. and J. Raes, *Microbial interactions: from networks to models.* Nature Reviews Microbiology, 2012. **10**(8): p. 538-550.
15. Forslund, K., et al., *Disentangling disease and drug signatures of the human gut microbiome: the case of type 2 diabetes mellitus.* 2015: p. 1-30.
16. Li, J., et al., *An integrated catalog of reference genes in the human gut microbiome.* Nature biotechnology, 2014.
17. Nielsen, H.B.o.r., et al., *Identification and assembly of genomes and genetic elements in complex metagenomic samples without using reference genomes.* Nature biotechnology, 2014: p. 1-11.
18. Martens, D., et al., *Performance of classification models from a user perspective.* Decision Support Systems, 2011. **51**(4): p. 782-793.
19. Goodman, B. and S.R. Flaxman, *European Union Regulations on Algorithmic Decision-Making and a "Right to Explanation".* AI magazine, 2017.
20. Ustun, B. and C. Rudin, *Optimized Risk Scores*, in *Proceedings of the 23rd ACM SIGKDD International Conference on Knowledge Discovery and Data Mining*. 2017, ACM: Halifax, NS, Canada. p. 1125-1134.
21. Tibshirani, R., *Regression shrinkage and selection via the lasso.* Journal of the Royal Statistical Society. Series B (Methodological), 1996: p. 267-288.
22. Tibshirani, R.J., *In praise of sparsity and convexity.* Past, Present, and Future of Statistical Science. Chapman Hall, London, 2014.
23. Pasolli, E., et al., *Accessible, curated metagenomic data through ExperimentHub.* Nature methods, 2017. **14**(11): p. 1023-1024.

24. Aron-Wisnewsky, J., et al., *Major microbiota dysbiosis in severe obesity: fate after bariatric surgery.* Gut, 2018.
25. Chevaleyre, Y., F. Koriche, and J.-D. Zucker, *Rounding Methods for Discrete Linear Classification.* Journal of Machine Learning Research, 2013. **28**(1): p. 651–659.
26. Pasolli, E., et al., *Machine Learning Meta-analysis of Large Metagenomic Datasets: Tools and Biological Insights.* PLoS Computational Biology, 2016. **12**(7): p. e1004977.
27. Affeldt, S., et al., *Spectral consensus strategy for accurate reconstruction of large biological networks.* BMC bioinformatics, 2016. **17**(Suppl 16): p. 493.
28. Freilich, S., et al., *Competitive and cooperative metabolic interactions in bacterial communities.* Nature Communications, 2011. **2**(1): p. 589.
29. Connor, E.F. and D. Simberloff, *The Assembly of Species Communities - Chance or Competition.* Ecology, 1979. **60**(6): p. 1132-1140.
30. Morton, J.T., et al., *Balance Trees Reveal Microbial Niche Differentiation.* mSystems, 2017. **2**(1): p. e00162-16-11.
31. Rivera-Pinto, J., et al., *Balances: a New Perspective for Microbiome Analysis.* mSystems, 2018. **3**(4): p. 174-12.
32. Bajaj, J.S., et al., *Altered profile of human gut microbiome is associated with cirrhosis and its complications.* Journal of Hepatology, 2014. **60**(5): p. 940-947.
33. Krajmalnik-Brown, R., et al., *Effects of Gut Microbes on Nutrient Absorption and Energy Regulation.* Nutrition in clinical practice : official publication of the American Society for Parenteral and Enteral Nutrition, 2012. **27**(2): p. 201-214.
34. Liu, Q., et al., *Synbiotic modulation of gut flora: Effect on minimal hepatic encephalopathy in patients with cirrhosis.* Hepatology, 2004. **39**(5): p. 1441-1449.
35. Winer, B.Y. and A. Ploss, *Breaking the species barrier for hepatitis delta virus.* Hepatology, 2015. **63**(1): p. 334-336.
36. Shreiner, A.B., J.Y. Kao, and V.B. Young, *The gut microbiome in health and in disease.* Current Opinion in Gastroenterology, 2015. **31**(1): p. 69-75.
37. Holmstrøm, K., et al., *Subdoligranulum variabile gen. nov., sp. nov. from human feces.* Anaerobe, 2004. **10**(3): p. 197-203.
38. Robert, C., et al., *Bacteroides cellulosilyticus sp. nov., a cellulolytic bacterium from the human gut microbial community.* International Journal of Systematic and Evolutionary Microbiology, 2007. **57**(7): p. 1516-1520.
39. Lv, L.-X., et al., *Alterations and correlations of the gut microbiome, metabolism and immunity in patients with primary biliary cirrhosis.* Environmental Microbiology, 2016. **18**(7): p. 2272-2286.
40. Hughes, E.R., et al., *Microbial Respiration and Formate Oxidation as Metabolic Signatures of Inflammation-Associated Dysbiosis.* Cell Host and Microbe, 2017. **21**(2): p. 208-219.
41. Shen, T.C., et al., *Engineering the gut microbiota to treat hyperammonemia.* J Clin Invest, 2015. **125**(7): p. 2841-50.
42. Vandeputte, D., et al., *Quantitative microbiome profiling links gut community variation to microbial load.* Nature, 2017: p. 1-21.
43. Poptsova, M.S. and J.P. Gogarten, *Using comparative genome analysis to identify problems in annotated microbial genomes.* Microbiology, 2010. **156**(Pt 7): p. 1909-17.
44. Hanson, A.D., et al., *'Unknown' proteins and 'orphan' enzymes: the missing half of the engineering parts list--and how to find it.* Biochem J, 2009. **425**(1): p. 1-11.
45. Forslund, K., et al., *Disentangling type 2 diabetes and metformin treatment signatures in the human gut microbiota.* Nature, 2015: p. 1-12.
46. Burke, H.B., *Predicting Clinical Outcomes Using Molecular Biomarkers.* Biomarkers in Cancer, 2017. **8**: p. BIC.S33380-11.

Figure Legends

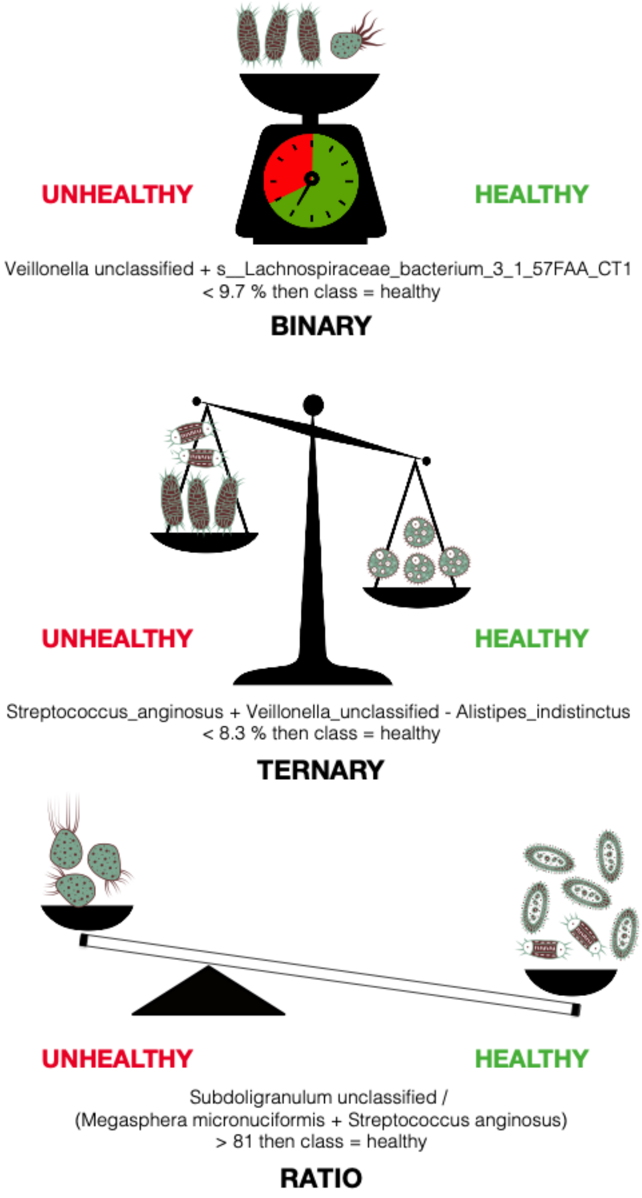

Figure 1: The three balance concepts depicting the BTR models

Top: The Binary model tests whether the cumulated abundance of a set of species is below or above a certain threshold. *Middle*: The Ternary model tests whether the cumulated abundance of a first set of species is below or above the cumulated abundance of a second set of species plus a certain threshold. *Bottom*: The Ratio model tests whether the cumulated abundance of a first set of species over the cumulated abundance of a second set of species is above a given threshold.

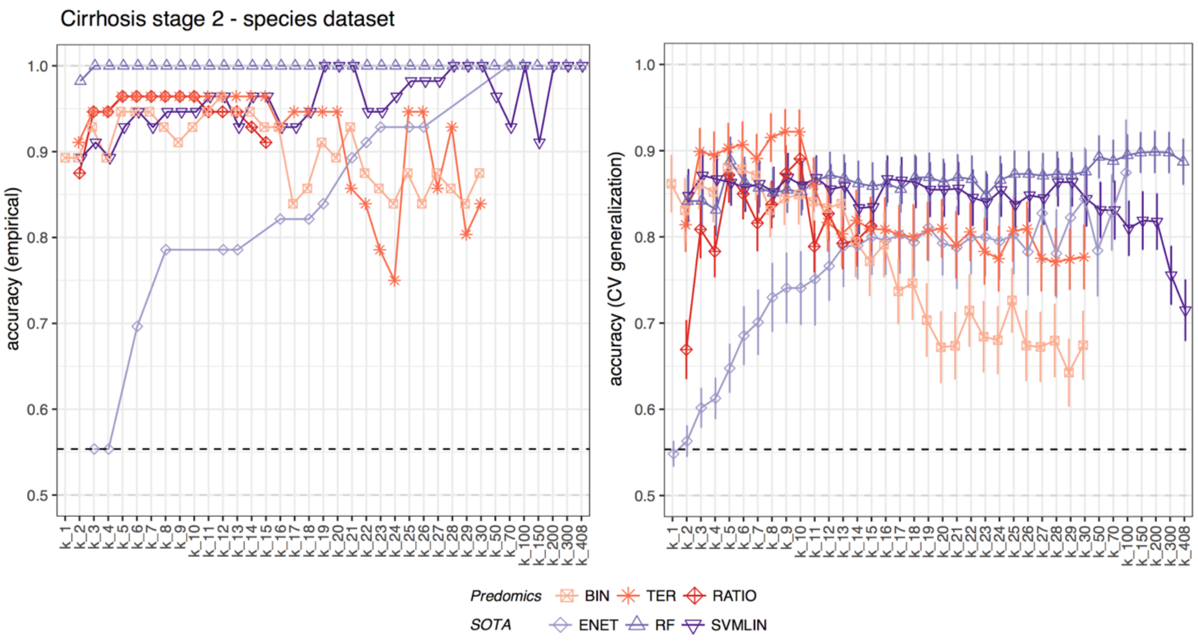

Figure 2: Model performance across different model-size

Left: Training accuracy of the best models (on the y-axis) in the Cirrhosis stage-2 dataset for different model-size *k* (indicated *k_#* on the x-axis). *Right:* Testing accuracy of the best models for each model-size as the average of 10-times, 10-fold cross validation sets +/- standard error of the mean. Dashed line indicates the majority class (i.e. the accuracy obtained when simply predicting the majority class through chance alone).

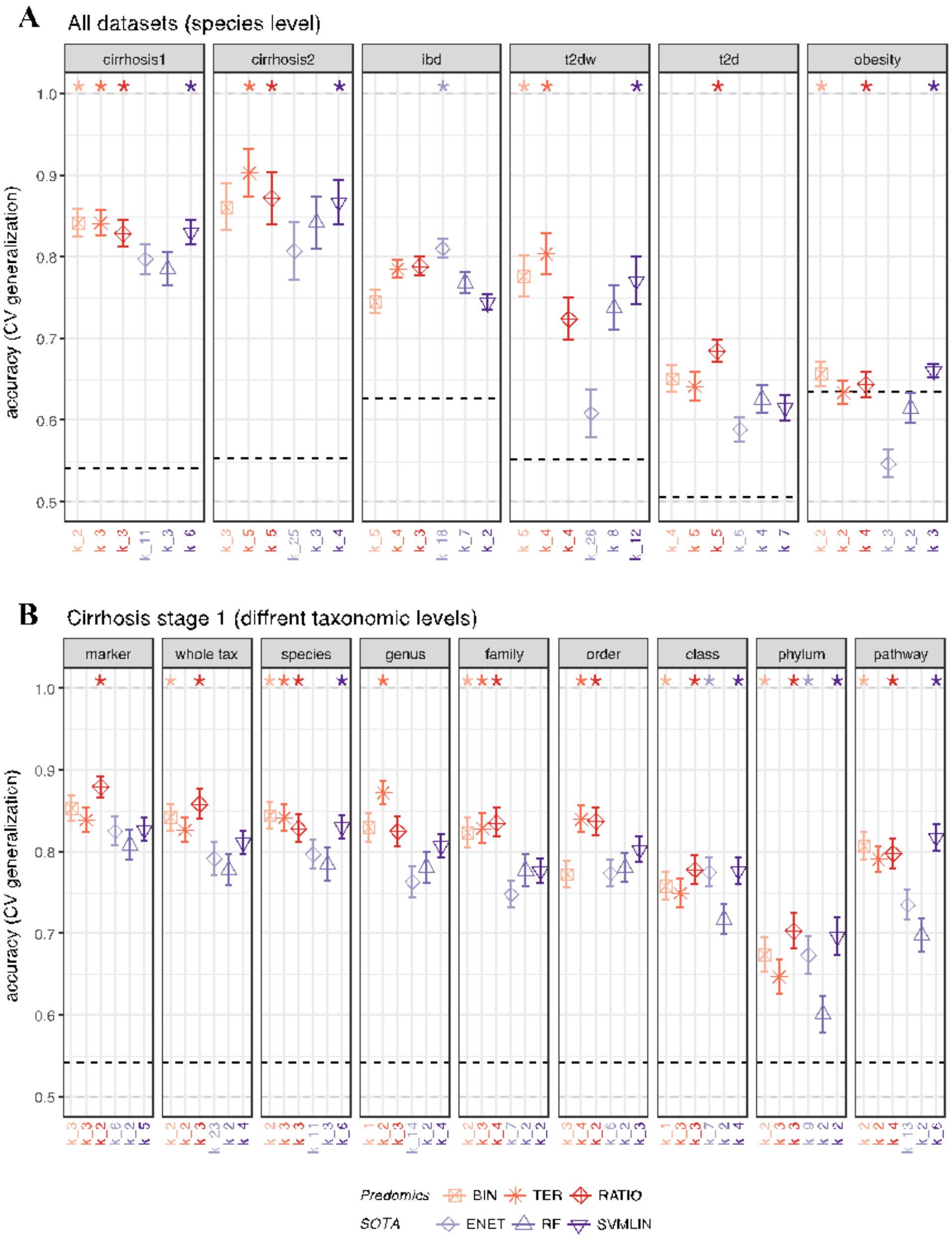

Figure 3: BTR models vs. SOTA performance across different diseases and taxonomic levels

A: Accuracy measured in the test datasets at the species level across six different datasets. The * on top indicate whether the corresponding BTR or SOTA algorithms are significantly better than others (*i.e.* without stars). **B**: Accuracy measured in the test datasets in different taxonomic levels of gut microbiome quantification (*species, genus, family, order, class and phylum, whole taxonomy*) as well as in marker gene and pathway abundance tables. Dashed bars indicate the majority class and *k_#* indicates the model-size. A 10-times 10-fold validation test values are summarized as mean +/- standard errors.

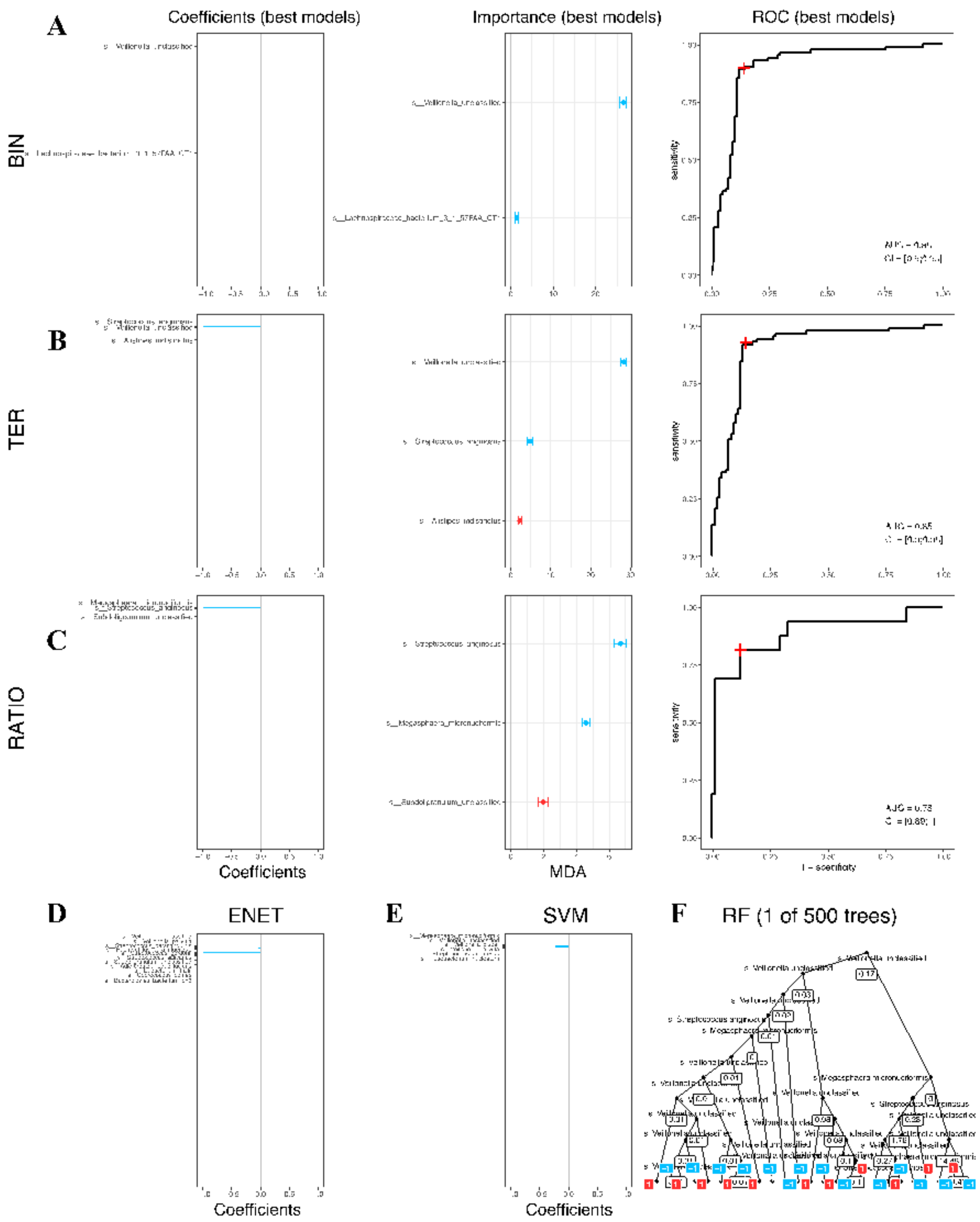

Figure 4: BTR models are interpretable compared to state-of-the-art

A-C *left*: Barcode graphical representations indicating the coefficients (1 or -1) of the BTR model features sorted by decreased correlation strength with the class to predict. **A-C** *middle*: Mean decrease accuracy (MDA) plots indicating feature importance computed during the cross-validation process. Blue and red colours indicate enrichment in patients and controls respectively. **A-C** *right*: Receiver operator characteristic (ROC) plots for the same BTR models. The red cross indicates the specificity and sensitivity of the model. **D-F**: A visualisation attempt of the SOTA models with barcode plots indicating the coefficients (values in [-1,1]) for ENET and SVMLIN, and only one tree out of the 500 used in the RF model.

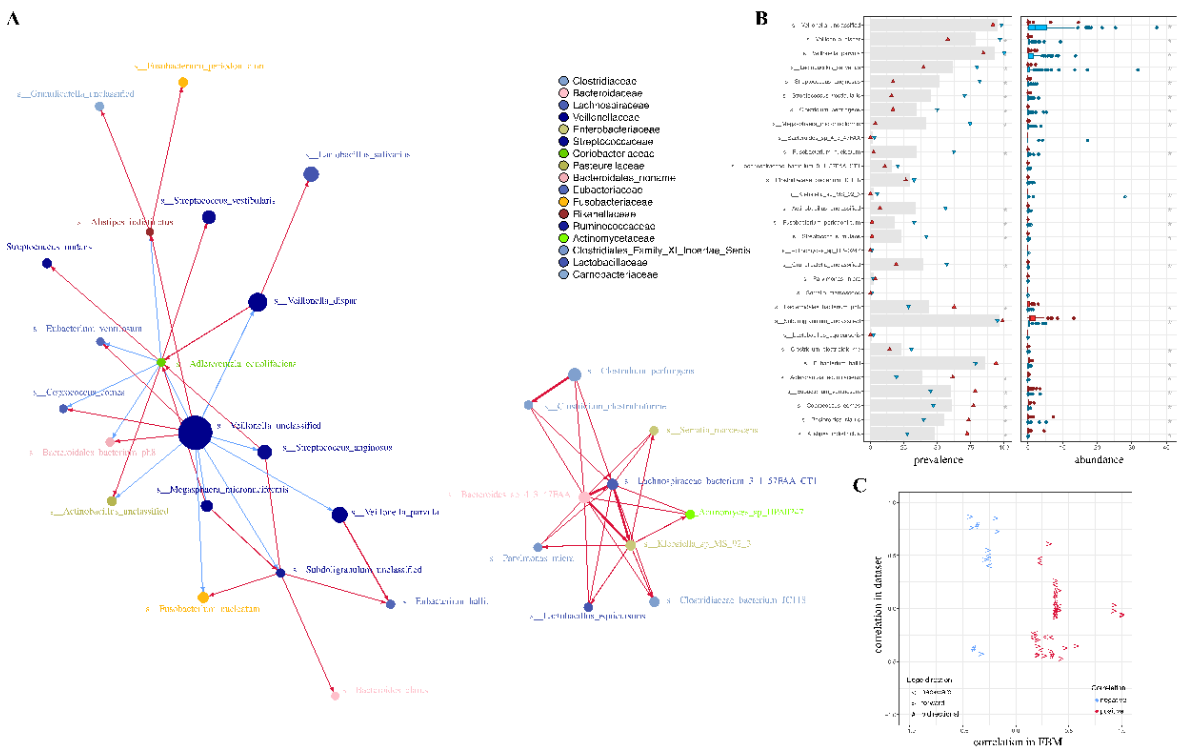

Figure 5: **Feature co-occurrence network in the family of best models**

A: This network displays feature co-occurrence patterns in FBM models. Only the top 5% strongest edges inferred using the ScaleNet network reconstruction approach (parameterized with *bayes_hc* and *aracne* algorithms, see methods) are shown. The size of the nodes is proportional to the average importance (MDA) in the BIN, TER and RATIO experiments. The colours of the nodes indicate the taxonomic family assignation as indicated in the legend. The red and blue edges indicate co-presence and co-absence in the models respectively. **B**: For each feature present in the network we show on the *left*: the prevalence of the features in the whole dataset (grey bar) and in the prediction classes (disease, healthy) depicted as blue and red dots respectively and in the *right*: the feature abundance distribution in the prediction classes (disease, healthy) depicted as blue and red box plots respectively. Grey stars indicate significant differences. **C**: A scatter-plot indicating for each edge of the network the correlation between the two features in the data and FBM respectively in the y- and x-axis. The colour is the same as for the edges in the network while the shape indicates the direction of the edges in the network.

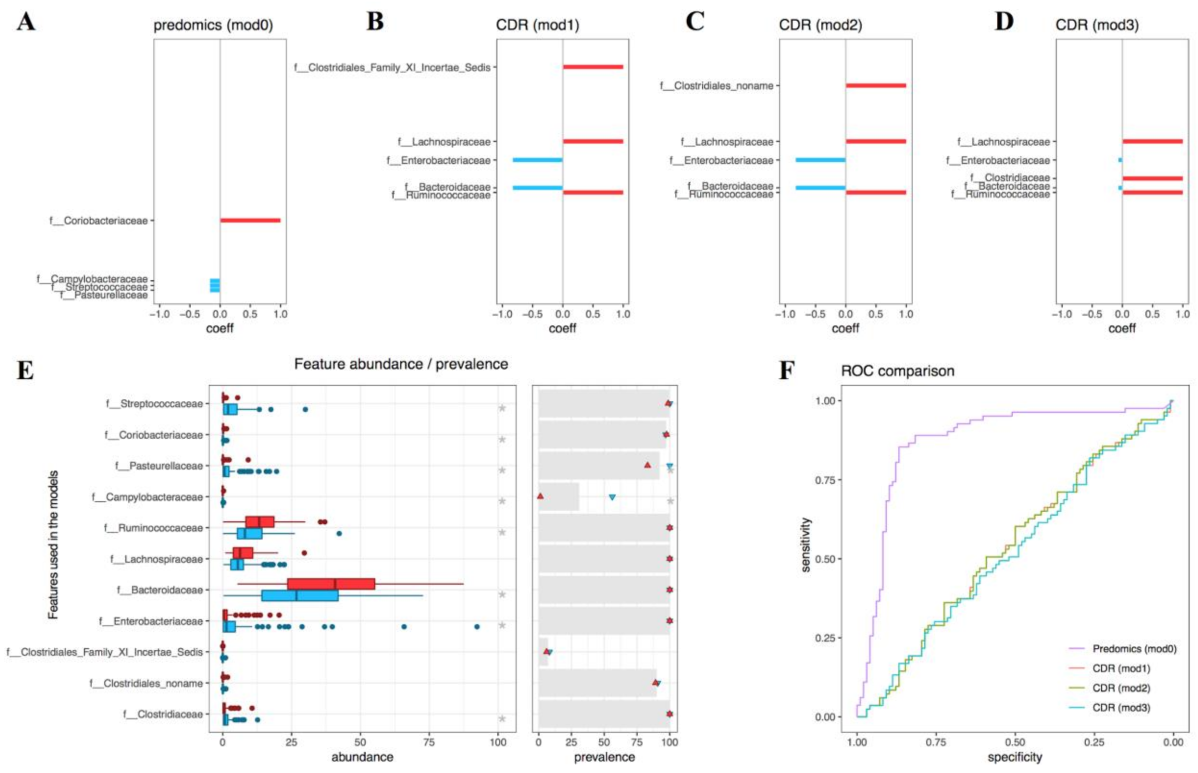

Figure 6: **Cirrhosis Dysbiosis Ratio (CDR) index compared to *predomics* ratio model.**

A-D: Barcode plots indicating the coefficients of the Ratio models *(S13-S15)* build with features from the CDR index and *predomics* discovered model *(S16)*. Red and blue colours indicate respectively the numerator and denominator of the ratio model and are respectively enriched in the controls and liver cirrhosis patients. The length of the lines is proportional to the ratio factor optimized in the model. **E** *left*: Boxplots indicating the abundance distribution by class for all features used in these models (red is enriched in controls and blue in the liver cirrhosis group). *right*: for the same features the prevalence of non-zero values is depicted in grey for the whole cohort and red and blue dots respectively in the control and patient groups. Grey stars indicate significant difference. **F**: Receiver operating characteristic (ROC) curves for the four models *(S13-S16)*.

URL for supplementary package

Click here to access/download
Supplementary Material
Supplementary package predomics address.pdf

Click here to access/download
Supplementary Material
Predomics_Supplementary Material_GigaScience_revised.docx

 Click here to access/download;Personal Cover;Predomics_Cover_letter_And_Response_To_Reviewers

Dear Dr. Nogoy,

Please accept our gratitude in allowing our work to be reviewed in Gigascience and accompanying us during this process.

We have carefully examined the review of Reviewer 1 and have answered them point-by-point in a constructive manner. We have performed additional time-consuming analyses to answer the questions and concerns raised by Reviewer 1 to the best of our ability (some questions were less explicit than others) and improved the main and supplementary text upon his advice.

Moreover, as already mentioned during our previous correspondence, we already registered the *predomics* software in the scicrunch.org repository as requested under the accession number RRID: SCR_017415 and mentioned it clearly in the revised version of the manuscript (page 15).

We hope that both the reviewers and the editorial board will find our answers and the new version of the manuscript satisfying.

With our best regards
Edi Prifti and Jean-Daniel Zucker

Reviewer reports:

Reviewer #1

This paper proposed a simple and interpretable disease classification prediction model, called predomics. It achieves the advantages that complex models do not have: the simplicity and interpretability of the model, while the overall performance on 54 datasets is better than the complex model (SOTA). The innovation is to infer the treatment effect and prognosis of patients through species abundance characteristics.

We thank the referee for his/her support and for reviewing our work. We would like to remind that we tested our approach on 109 different datasets (54 microbiome taxa/functional abundance datasets, 54 microbiome taxa/functional presence/absence datasets from different diseases as well as another dataset used to predict outcome of bariatric surgery in severely obese patients).

The main innovative aspects of this work are the models themselves, which are inspired by an ecosystemic consideration of the interactions between bacterial taxa, as illustrated in Figure 1 of the main text. This, along with their simplicity and performance allow for an increased interpretability when compared with other state-of-the-art models. Finally, the consideration of a family of best models (FBM) along with the feature importance indicators and their model co-presence analyses, improves even more the understanding of the microbiome predictive signatures from an ecosystemic perspective.

However, the problem with the article is: In the method section, the importance of features can be calculated.

Concerning the way feature importance is calculated, the procedure is straightforward as it consists in computing an out-of-bag (OOB) error for each feature of <u>each model of the family of best models (FBM)</u>. More precisely, given each model of the FBM, the importance of each of its features is measured by first permuting its values within the out-of-bag data and next by computing the consequent mean decrease of accuracy (MDA). Finally, the mean decrease accuracy (MDA) is computed as the average of these values over all the folds and is displayed along with the standard error of the mean. As mentioned in the method section of the main text (page 6), this procedure is similar to that of Random Forest feature importance algorithm.

We rephrased (see below next question) the explanation of the computation of the feature importance in the method section.

However, how to select the features that will be calculated is not directly explained.

We took much care to establish that Feature Importance is computed during each cross-validation fold and is defined as the usefulness of each feature for the model learned in the fold. The genetic algorithm (GA) approach (see methods and supplementary material) after the evolution process will generate a "population of models" out of which we identify a family of best models, defined as the set of models whose accuracy is within a statistically equivalent window, determined by a threshold when assuming a binomial distribution ($p<0.05$; see methods). The features that are found in these models are a result of the evolution process of the GA. <u>They are specifically the ones that are "selected" and the ones for which feature importance is computed</u>.

In the methods section of the revised version of the manuscript (page 6) we added the following phrase "...This is performed on all the features of the dataset that are found in the FBM models, on which errors before and after permutations are computed".

Maybe the species abundance of biomarkers?

Biomarkers can be abundance or presence/absence of any taxonomical or functional variables used in Microbiome studies.

In the results section, there still remain unclear of calculating Bin and Ter ratio which will be compared with a prediction threshold. For example, how to optimize the prediction threshold.

We thank the referee for raising this question. This technical aspect of implementation although fully described in the predomics package was not addressed in the main document and we have added a paragraph to clarify (see below). .

Regarding the computation of a BTR model (be it Bin, Ter or Ratio), it is an object containing different attributes, such as the list of features, the corresponding coefficients, the type of model as well as the threshold. The features of the BTR models are set when creating the model during the generation, mutation, crossover, etc, in the evolution process. The coefficients of the features in the dataset X are set to {-1 or 1} for Ter/Ratio models based on the average abundance for each feature in the different classes of y. For Bin models the coefficients are set to 1.

The threshold is optimized so that the performance of the classification is maximized in the training set, except in the case where the treschold is zero. Once the model is trained, the threshold will not change when testing. In short, the threshold optimization technique consists in computing the score of the model in the training set for each observation. The observations are ordered based on this score as illustrated in ***Figure R1 top****. The following step consists in computing the cumulative error in the same order as the score (****Figure R1 bottom****). Each time an example is misclassified, the cumulative error increases. The index on which this function is minimized (red vertical line* ***Figure R1 bottom****) will provide the score index, which will serve as a threshold to be set in the model (red horizontal line* ***Figure R1 top****).*

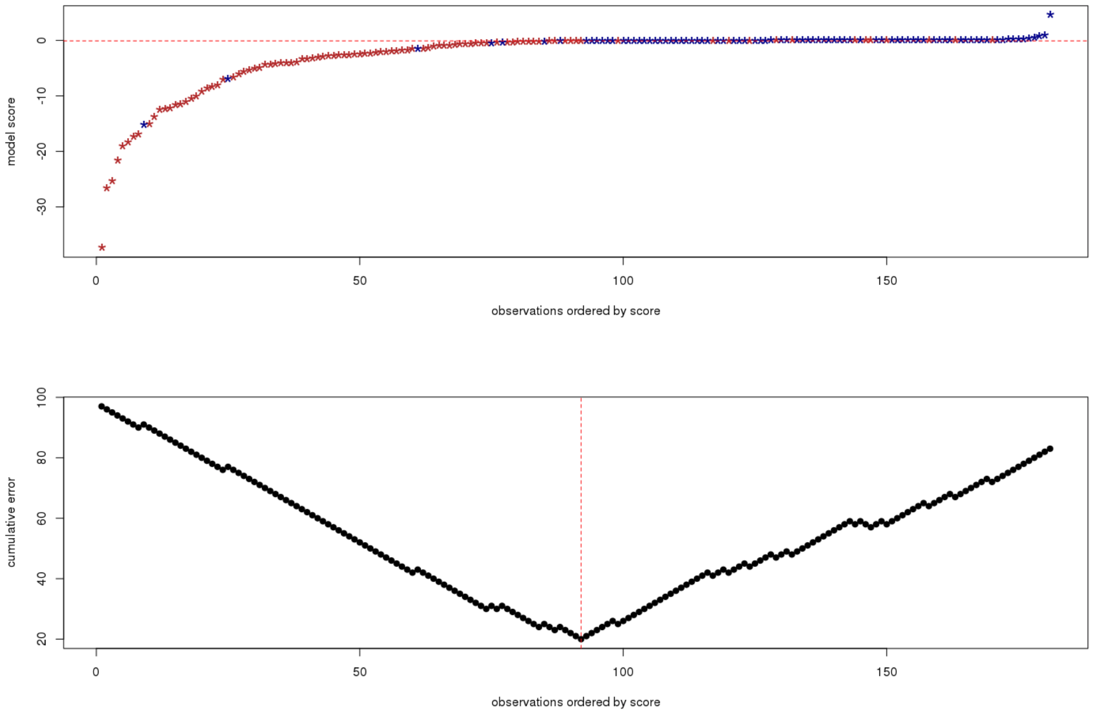

Figure R1: Threshold optimization process.
Top: the score of the model including the features and coefficients for different observations. The observations are ordered using this score. *Bottom:* the cumulative error of class calling following this score. Minimizing this error will provide the index of the observation on which the score will be used as a threshold.

We have added the following paragraph in the methods section (page 6) to describe the threshold optimization technique:

"The threshold used in the model is optimized to maximize the performance in the training set. This approach consists in computing the model's score for each observation in the training set. The observations are ordered based on this score. Next, the cumulative error is computed following the same ordering - each time an example is misclassified the cumulative error is increased when sliding through the score. The index example on which the cumulative error minimizes will provide the value of the score, which will be used as a threshold in the final model."

In the evaluation and comparison of the predomics with other models, such as classification tasks, in addition to the accuracy rate, more evaluation indicators should also be introduced, such as precision, recall, F1-score, etc.

The implementation of our approach in the predomics package automatically provides these performance scores (accuracy, precision, recall, f1, contingency table, etc.), as well as many functions allowing simple analyses and visualization of the results (see package vignettes). We have compared BTR models with SOTA using these alternative performance indicators and the main conclusions described using accuracy remain. As it was asked by the referee, we have produced additional figures to illustrate these additional evaluations. Figure R2 displays comparative results between BTR and SOTA models on the 54 normalized abundance datasets for the different scores recall, precision and f-score. Panels A, D and G indicate the datasets on which the BTR models are as good or better than SOTA. Panels B, E and H, indicate datasets when BTR are statistically better than SOTA. Finally, panels C, F and I, indicate datasets when SOTA are statistically better than BTR.

Overall these results are similar with results on accuracy for recall and f1-score – even though BTR outperforms SOTA for recall in most datasets. However, RF seems to display higher precision compared to BTR, while SVM and ENET display lower precision scores. This is better shown in Figure R3 below. Results for the F1-score are quite comparable.

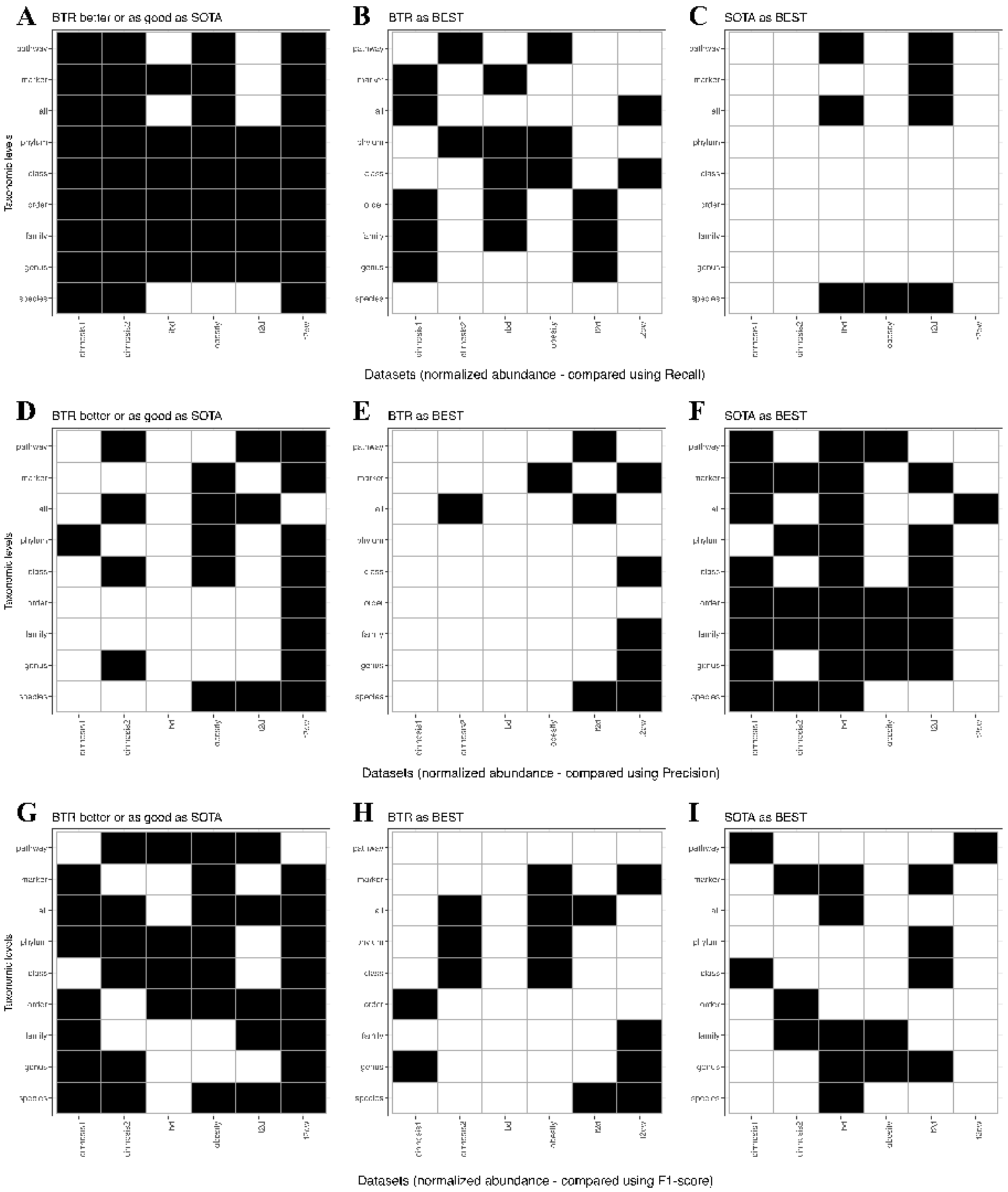

Figure R2: Best model performance across all experiments (penalization strategy) different measurements

A: The black colour indicates for each abundance dataset whether any of the three BTR models is among the best learners (as tested with a paired T-test for the 100 CV generalization recall) and white otherwise. **B**: The black colour indicates for each abundance dataset whether any of the three BTR models is significantly better than any of the SOTA in recall and white otherwise. **C**: The black colour indicates for each abundance dataset whether any of the three SOTA models is significantly better than any of the BTR in recall and white otherwise. **D-F**: similar as **A-C** but for precision. **G-I**: similar as **A-C** but for F1-score.

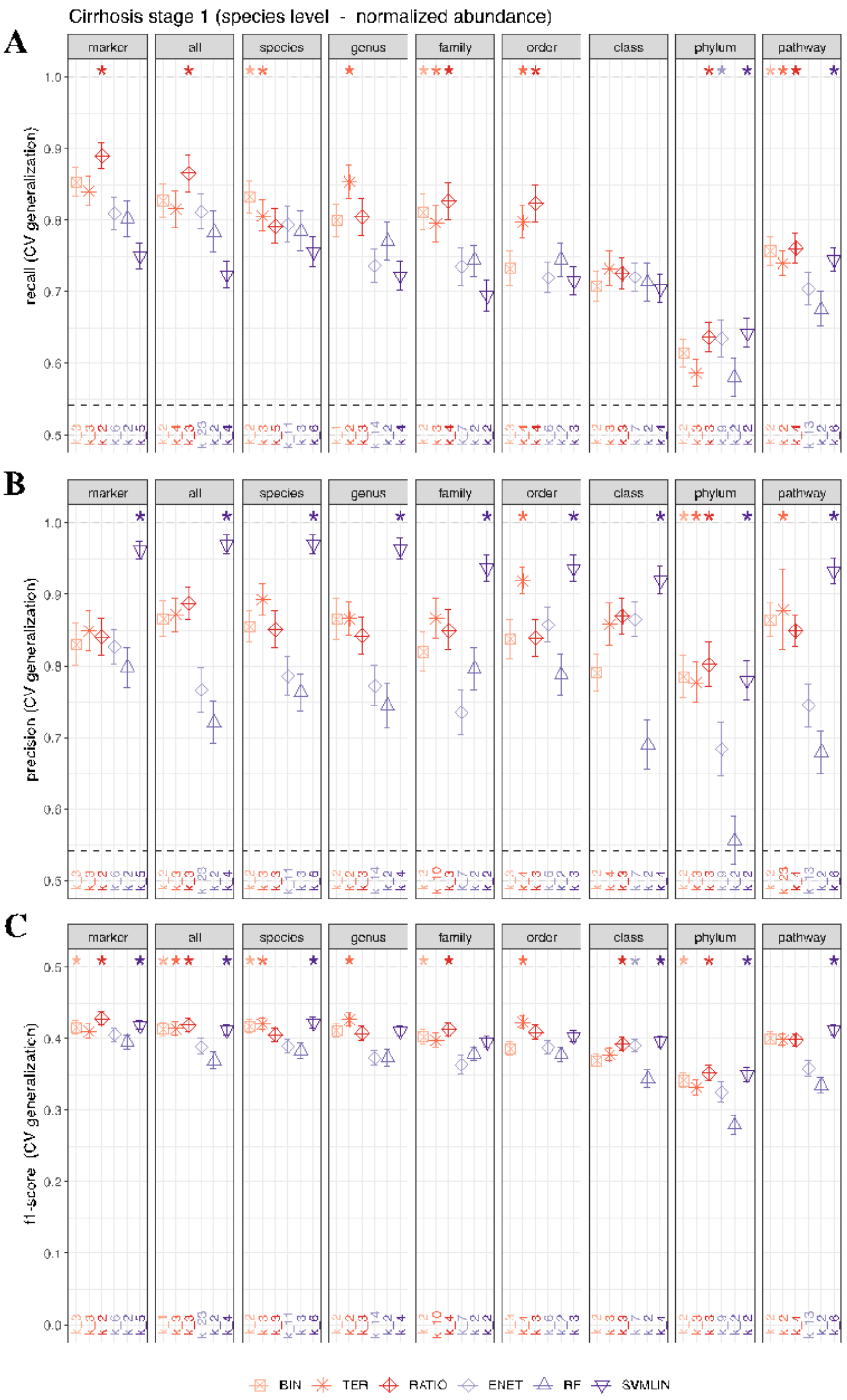

Figure R3: BTR and SOTA performance across different taxonomic levels in Cirrhosis stage 1 species normalized abundance data

A: Recall measured in the test datasets in different taxonomic levels of gut microbiome quantification with normalized abundance (*species, genus, family, order, class and phylum, whole taxonomy*) as well as in marker gene and pathway abundance tables. **B**: Precision measured in the test datasets in different taxonomic levels of gut microbiome quantification with normalized abundance (*species, genus, family, order, class and phylum, whole taxonomy*) as well as in marker gene and pathway abundance tables. **C**: F1-score measured in the test datasets in different taxonomic levels of gut microbiome quantification with normalized abundance (*species, genus, family, order, class and phylum, whole taxonomy*) as well as in marker gene and pathway abundance tables. *k_#* indicates the model-size of the best model as identified in training. 10 times 10-fold cross-validation values are summarized as mean +/- standard errors. The * on top indicate whether the corresponding BTR or SOTA algorithms are significantly better than others (*i.e.* without stars).

Both figures R2 and R3 have been added to the supplementary materials as figures S6 and S7.

In the discussion section, a more thorough discussion is needed on the interpretable characteristics of the model. The recommended way is to combine the important features given by the predomics model with the patient's gut microbiome data to find potential biomarkers and then make a further explanation.

We thank the referee for his/her suggestion. Indeed, besides the two-page discussion of the main text entitled "BTR models provide biological insights" (page 11-12), we have discussed extensively the interpretability aspects of the BTR models in two paragraphs of the supplementary material entitled "BTR models provide relevant biological interpretation in liver cirrhosis" (page 13-16) and "Models of bariatric surgery-induced metabolic improvements based on BTR regression" (page 16-17). We have provided in this article a very dense experimental design as well as compelling results showing that our original approach allows discovering interpretable and accurate models.

We thank the referee for having reviewed our work as well as for his/her constructive comments that we believe have improved the paper.

Reviewer #2

The authors have sufficiently addressed my questions and concerns.

We thank the referee for having reviewed our work as well as for his/her constructive comments that we believe have improved the paper.
