# Supplementary material for: Interpretable and accurate prediction models for metagenomics data
Source: Gigascience. 2020 Mar 9;9(3):giaa010. doi: 10.1093/gigascience/giaa010 (PMC7062144; doi:10.1093/gigascience/giaa010)
Supplement: giaa010_Supplemental_Files [file giaa010_supplemental_files.zip › Predomics_Supplementary_Material_GigaScience_revised3.docx]

### SUPPLEMENTARY MATERIAL

### Dataset information

We tested *predomics* on several public datasets. For the classification tasks we downloaded five curated metagenomic datasets from the ExperimentHub [25]. The raw data were generated in independent studies using shotgun metagenomics (**Table S1**) and were processed bioinformatically and curated by Pasolli et al [25]. For the regression experiments, we used shotgun metagenomics data from a recently published study, where morbidly obese patients underwent bariatric surgery [26] (see online methods).

| **Dataset Name** | **Disease** | **# features**  *(species, genus, family, order, class, phylum, whole_tax, marker, pathway)* | **# cases** | **# controls** | **Average Reads**  **(std) (M)** | **Type of Task** | **Reference** |
| --- | --- | --- | --- | --- | --- | --- | --- |
| cirrhosis1 | Liver cirrhosis stage 1 | 462, 151, 52, 22, 15, 9, 1252, 128224, 310 | 98 | 83 | 51.6  (30.9) | classification | [11] |
| cirrhosis2 | Liver cirrhosis stage 2 | 408, 118, 53, 22, 15, 9, 990, 86308, 306 | 25 | 31 | 51.6  (30.9) | classification | [11] |
| ibd | Inflammatory  bowel disease | 719, 299, 141, 64, 33, 21, 1934, 222837, 427 | 148 | 248 | 53.9  (20.2) | classification | [17] |
| t2dw | Type 2  diabetes | 381, 142, 39, 29, 24, 14, 943, 91102, 430 | 53 | 43 | 31.0  (17.6) | classification | [48] |
| t2d | Type 2 diabetes | 505, 222, 98, 45, 14, 8, 1463, 131309, 431 | 170 | 174 | 40.2  (11.8) | classification | [10] |
| obesity | Obesity | 429, 243, 121, 60, 31, 20, 1365, 128510, 418 | 167 | 96 | 69.0  (23.2) | classification | [8] |
| microbaria | Bariatric surgery | 558 | 24 | - | 41.83 (19) | regression | [26] |

**Table S1: Summary of the datasets considered in the experiments.**

For each public dataset tested, number of features, cases and controls are provided as well the learning task (classification or regression). The original reference of the studies is also provided.

### Sketch of the Predomics Algorithm

Based on a genetic algorithm, (i.e. heuristic commonly used to approximate solutions of complex problems), *predomics* supports learning high-quality models (see methods). From a ML perspective, learning BTR models corresponds to minimizing the sum of a cost function (e.g. residual sum of squares (RSS)) and a L1 norm regularization for the sparsity, under a constraint on the unary value of the linear model that predicts classes.

1. The first step is the generation of an initial set of candidate models called the “initial population” $P_{t}$, typically composed of 100 random models. Although usually this consists of randomly drawing candidate models, in our case, we combine models generated by a beam-search algorithm, models obtained by a logistic regression followed by a weight discretization phase ([46]) and purely random models. These models are chosen of different sizes (i.e. parsimony), typically $\in\{1 :30\}$.
2. Then, the algorithm performs 100 iterations. At each iteration $t$, the algorithm generates a new set models $P_{t+1}$ (a new population) based on the previous population $P_{t}$. To build $P_{t+1}$, the algorithm performs four consecutive stages, which are the *evaluation, selection, crossover* and *mutation*.
   1. All models in $P_{t}$ are evaluated according to their predictive accuracy/regression. Each of the three remaining stages outputs a modified population based on the population of the previous stage.
   2. Typically, 50% of the models are selected half randomly and half based on the performance. This selection will be at the origin of the new generation of models $P_{t+1}$.
   3. During the cross-over, pairs of models are randomly drawn among those who survived the selection stage, and their features are combined randomly to generate new models, which are added to the population.
   4. In the mutation stage, randomly selected models are mutated. The mutation consists on either removing, adding a random feature, or even altering the weights of one or more features. At this stage the $P_{t+1}$ is created and will serve as initial population of $P_{t+2}$ and so on.
3. At the end of the evolution process, a final population of models $P_{final}$ is provided. The best model is obtained by applying a model-size penalization, which is $a{ccuracy}_{penalized}= accuracy -ƛ k$. *k* is the number of features in the model (i.e. parsimony) and $ƛ$ is an hyperparameter controlling the penalization of the accuracy. In the experiments described in this paper we used $ƛ=1\%$ (*i.e.* a model that is using one additional feature will only be preferred if it improves the performance of more than 1%).

### Model performance is dependent on model-size

The *predomics* approach has an embedded feature selection step (see online methods), which supports finding BTR models in a given model-size (i.e. parsimony) range. We explored model performance with different number of features. In our experiments, the number of selected features for the BTR models, ranges from {1..30}. On the other hand, for the current experiment, we applied an external feature selection based on the Mann-Whitney statistical test for the SOTA algorithms. The *k* top most significant features were selected and the subsequent restricted datasets were used to learn the different SOTA algorithms. For ENET, we selected the first *k_#* features from those identified by the algorithm in the regularisation path. The number of selected features for the SOTA ranges in {1..30, 50, 70, 100, 150, 200, 300, *min(1000, total number of features)*}. Results from the cirrhosis stage-2 species dataset, illustrated in **Figure 2** show the *empirical accuracy* of the best models for each model-size k (*left*), and the average generalization accuracy for 10-times 10-fold cross-validation +/- standard error of the mean (*right*).

Of note, the SOTA algorithms display a more pronounced difference between empirical and generalization accuracy. Indeed, algorithms such as random forest (RF) learn well and fit the training data even with low number of features (k=2) but with a large number of trees (n=500 here), while it takes more features for ENET (k=70) and SVMLIN (k=19) to reach their maximal accuracy on the training set. The important difference between the training and generalization accuracy, illustrates overfitting, probably due to the very large number of parameters to learn and the small number of examples. The accuracy drops from 1 in training to a maximum of 0.86 in testing.

On the contrary, BTR models display better generalization and this with just a few features (k=4). BTR models outperform all SOTA methods, reaching an accuracy=0.92. Both empirical and generalization accuracies are similar indicating good generalization of the model. Similar trends are observed in the other tested datasets (data not shown). Interestingly, we notice that the best performance of BTR models is obtained for model-size *k* within the range {4..11}. This suggests that few features only are sufficient to learn efficient models. Adding additional ones tend to worsen the performance because of probable overfitting. Moreover, the number of permutations increase with *k,* making it more difficult to converge towards optimal solutions.

### Both metagenomic presence or abundance data display high classification power

We originally evaluated the performance of BTR models and compared their performance with SOTA in the whole experimental space on abundance data (*i.e.* a total of 54 experiments, consisting of 6 datasets for each of the 9 variable types as described above). We also tested our models on presence/absence binary data derived from these same 54 datasets. The results are shown in **Figure S1*.*** For both abundance and presence datasets using the model-size penalization strategy — the BTR models are significantly among the best for most datasets (**Figure S1** *left*). For presence/absence data (**Figure S1D-F)**, BTR performed at least as well as SOTA in 40/54 (74%) of the experiments and outperformed SOTA in 9/54 (17%), while the SOTA outperformed BTR in 14/54 (26%) of the cases. Indeed, the sparsity of microbiome data allows for presence/absence derived datasets to predict with relatively high accuracy, although lower compared to the abundance tables, especially in higher taxonomic levels since they become less sparse. We obtain similar results for abundance but also for presence/absence data even when not applying the penalization strategy to SOTA models (including all variables of the dataset; **Figure S2**) or even when fixing the same number of features for BTR and SOTA models (k = 5; **Figure S3**).

**Figure S1: Best model performance across all experiments (penalization strategy)**

**A**: The black colour indicates for each abundance dataset whether any of the three BTR models is among the best learners (as tested with a paired T-test for the 100 CV generalization accuracies) and white otherwise. **B**: The black colour indicates for each abundance dataset whether any of the three BTR models is significantly better than any of the SOTA and white otherwise. **C**: The black colour indicates for each abundance dataset whether any of the three SOTA models is significantly better than any of the BTR and white otherwise. **D-F**: similar as **A-C** but for the presence/absence derived data.

**Figure S2: Best model performance across all experiments (no-penalization for SOTA)**

**A**: The black colour indicates for each abundance dataset whether any of the three BTR models is among the best learners (as tested with a paired T-test for the 100 CV generalization accuracies) and white otherwise. **B**: The black colour indicates for each abundance dataset whether any of the three BTR models is significantly better than any of the SOTA and white otherwise. **C**: The black colour indicates for each abundance dataset whether any of the three SOTA models is significantly better than any of the BTR and white otherwise. **D-F**: similar as **A-C** but for the presence/absence derived data.

**Figure S3: Best model performance across all experiments (fixed k = 5 for BTR and SOTA)**

**A**: The black colour indicates for each abundance dataset whether any of the three BTR models is among the best learners (as tested with a paired T-test for the 100 CV generalization accuracies) and white otherwise. **B**: The black colour indicates for each abundance dataset whether any of the three BTR models is significantly better than any of the SOTA and white otherwise. **C**: The black colour indicates for each abundance dataset whether any of the three SOTA models is significantly better than any of the BTR and white otherwise. **D-F**: similar as **A-C** but for the presence/absence derived data.

**Figure S4** indicates in more detail the best models for the different datasets at the species level (top) and for the different taxonomic levels for the Cirrhosis stage-1 dataset but this time using only presence/absence data. Similarly, to **Figure 2** (in the main text), where abundance data were used, very simple BTR models (see *S1, S2, S3* below for examples) trained with presence/absence derived datasets, allow predicting with relatively high performance. For instance, the model *(S1)* can be interpreted as follows. If at least one of the three species *s__Streptococcus_anginosus, s__Veillonella_atypica and s__Streptococcus_infantarius* is present in the microbiome of a patient, then he/she is unhealthy. These models are comparable in performance to more complex SOTA models. Such good results can be explained by the overall sparsity of microbiome data that makes the discovery of such patterns possible. Noteworthy, not all datasets are easy to predict, some diseases such as obesity and t2d, display weaker associations with gut microbiome when compared to cirrhosis and IBD for instance.

*(S1) ([s__Streptococcus_anginosus]>0) + ([s__Veillonella_atypica]>0) + ([s__Streptococcus_infantarius]>0) < 2* ***then*** *class = healthy*

*(S2) ([s__Megasphaera_micronuciformis]>0) + ([s__Streptococcus_anginosus]>0) + ([s__Actinobacillus_unclassified]>0) + ([s__Veillonella_atypica]>0) + ([s__Streptococcus_infantarius]>0) - ([s__Alistipes_indistinctus]>0) < 2* ***then*** *class = healthy*

*(S3) ([s__Roseburia_hominis]>0) + ([s__Ruminococcus_bromii]>0) + ([s__Orthohepadnavirus_unclassified]>0) / ([s__Megasphaera_micronuciformis]>0) + ([s__Streptococcus_infantarius]>0) > 2* ***then*** *class = healthy*

**Figure S4**: **BTR and SOTA performance across different disease and taxonomic levels in presence/absence data**

**A**: Accuracy measured in the test datasets at the species level across six different datasets in presence/absence data. The *** on top indicate whether the corresponding BTR or SOTA algorithms are significantly better than others (*i.e.* without stars). **B**: Accuracy measured in the test datasets in different taxonomic levels of gut microbiome quantification with presence/absence information (*species, genus, family, order, class and phylum, whole taxonomy*) as well as in marker gene and pathway abundance tables. Dashed bars indicate the majority class and *k_#* indicates the model-size of the best model as identified in training. 10 times 10-fold cross-validation values are summarized as mean +/- standard errors.

Finally, we evaluated the performance of our models in a second, independent dataset. For this, we learned Bin, Ter, Ratio and also TerLog models in the Cirrhosis stage-1 (species) dataset with tested them in the Cirrhosis stage-2 (species) dataset. Results illustrated in **Figure S5** indicate very good external validation with a mean training accuracy of 0.89 (sd=0.02) and a mean testing accuracy of 0.85 (sd=0.04). Importantly, TER and RATIO models generalize better than Bin and TerLog. We hypothesize that the multiplicative relationship in the TerLog models may be difficult to learn due to the power of the scaling factor.

###
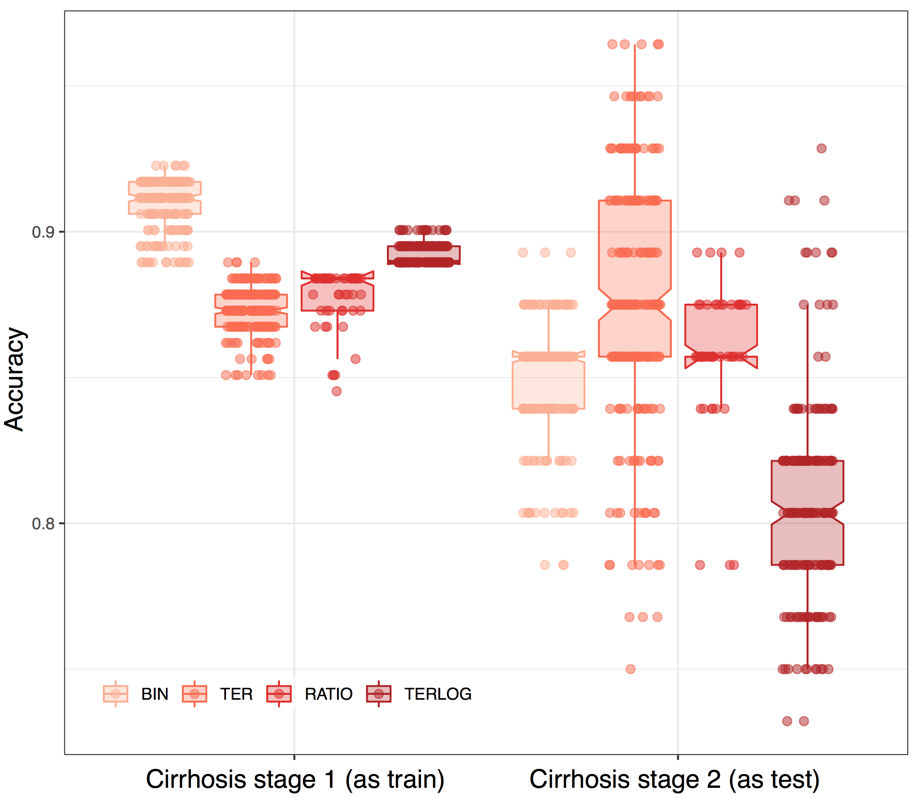


**Figure S5**: **Validation of BTR models in an external dataset**

The BTR models learned on the entire Cirrhosis stage-1 (species) dataset were tested on an independent Cirrhosis stage-2 (species) dataset. *Left* (resp. *Right*) the empirical (resp. generalization) accuracy of BTR models on the testing (resp. training) datasets is displayed as boxplots separated by model type.

The implementation of our approach in the *Predomics* package automatically provides several widely used performance scores (accuracy, precision, recall, f1, contingency table, etc.), as well as many functions allowing simple analyses and visualization of the results (see package vignettes). Here, we have compared BTR models with SOTA using also these alternative performance indicators and the main conclusions described above using accuracy remain.

We have provided additional results in **Figure S6** comparing the performance (recall, precision and F1 score) results between BTR and SOTA models on the 54 normalized abundance datasets. Panels A, D and G indicate the datasets on which the BTR models are as good or better than SOTA. Panels B, E and H, indicate datasets when BTR are statistically better than SOTA. Finally, panels C, F and I, indicate datasets when SOTA are statistically better than BTR. Overall these results are similar with results on accuracy for recall and f1-score – even though BTR outperforms SOTA for recall in most datasets. However, RF seems to display higher precision compared to BTR, while SVM and ENET display lower precision scores then BTR. This is better shown in **Figure S7** below. Results for the F1-score are quite comparable with recall.

**Figure S6: Best model performance across all experiments (penalization strategy) different measurements**

**A**: The black colour indicates for each abundance dataset whether any of the three BTR models is among the best learners (as tested with a paired T-test for the 100 CV generalization recall) and white otherwise. **B**: The black colour indicates for each abundance dataset whether any of the three BTR models is significantly better than any of the SOTA in recall and white otherwise. **C**: The black colour indicates for each abundance dataset whether any of the three SOTA models is significantly better than any of the BTR in recall and white otherwise. **D-F**: similar as **A-C** but for precision. **G-I**: similar as **A-C** but for F1-score.

**Figure S7**: **BTR and SOTA performance across different taxonomic levels in Cirrhosis stage 1 species normalized abundance data**

**A**: Recall measured in the test datasets in different taxonomic levels of gut microbiome quantification with normalized abundance (*species, genus, family, order, class and phylum, whole taxonomy*) as well as in marker gene and pathway abundance tables. **B**: Precision measured in the test datasets in different taxonomic levels of gut microbiome quantification with normalized abundance (*species, genus, family, order, class and phylum, whole taxonomy*) as well as in marker gene and pathway abundance tables. **C**: F1-score measured in the test datasets in different taxonomic levels of gut microbiome quantification with normalized abundance (*species, genus, family, order, class and phylum, whole taxonomy*) as well as in marker gene and pathway abundance tables. *k_#* indicates the model-size of the best model as identified in training. 10 times 10-fold cross-validation values are summarized as mean +/- standard errors. The *** on top indicate whether the corresponding BTR or SOTA algorithms are significantly better than others (*i.e.* without stars).

Altogether our results indicate that BTR models, although composed of just a few features, offer very good performance in classification compared to the best state-of-the-art methods, both in abundance and presence/absence derived data.

### Top features from the family of BTR models improve interpretability

For each of the three experiments, where Bin, Ter and Ratio models are searched to predict the Cirrhosis stage-1 (species) dataset, we have selected the best family of models (i.e. FBM). This is performed by applying a statistical threshold above which the performance of a model is not significantly different from the best model (see online methods). Here we applied a second condition and retained only models of a maximum size of 5 in order to reflect the observations of the average model size in this dataset. These three FBM are pooled together and used in the analyses below. **Figure S8**, illustrates the distribution of model size (*i.e.* number of features) depicted by different colours as a function of the performance (*i.e.* accuracy in the whole dataset) and this for each model type. We observe that most of the models are Ter (n=241) and more particularly of size 3 and 4. There are 4 different Ter models with the same accuracy, which is also the highest (0.89). All best Bin models (n=7) are of size 4 and 5, meaning that smaller ones were not selected in the FBM. Finally, best Ratio models (n=20) are composed of 4 and 5 features.


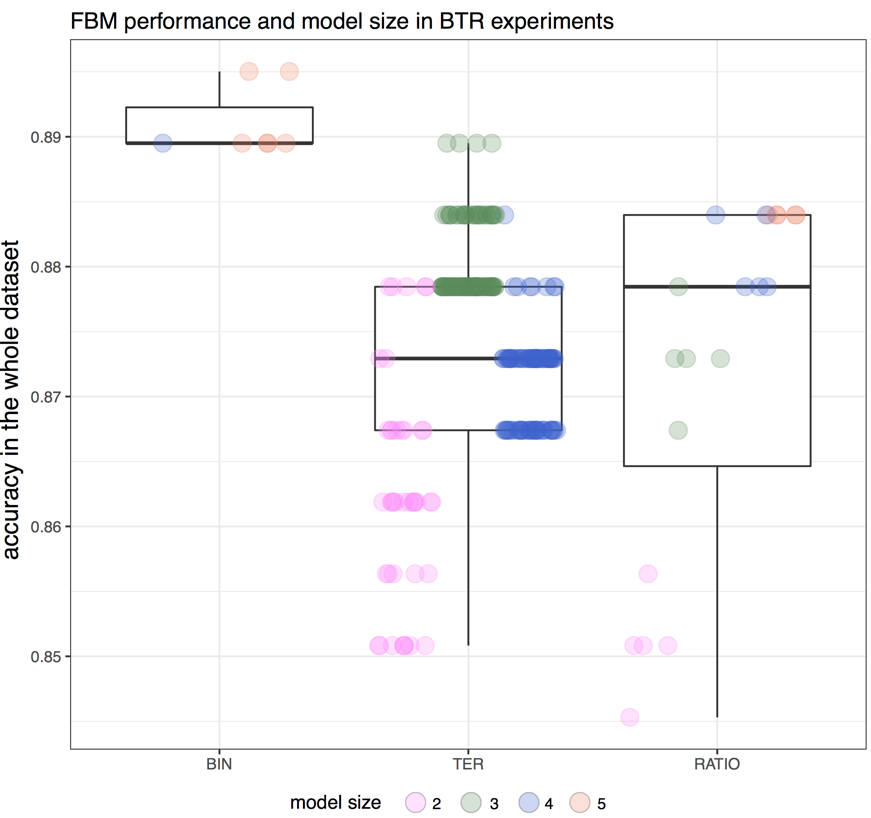


**Figure S8: Performance of the family of best models (FBM) across model-size and model type**

The accuracy of the FBM models for Bin, Ter and Ratio in the whole Cirrhosis stage-1 (species) dataset is described as a factor of model-size. Models with different size (*i.e.* number of features) are represented as points of different colours. Points are scattered in the x-axis for better visualisation of the density distribution.

Next, we analysed the 169 features (37%, out of the 459 in the whole dataset) selected by the 268 models of the three FBM. The corresponding MDA (mean decrease accuracy) as computed during the cross-validation process (see online methods) are displayed in **Figure S9** *left*. Features are ordered as the average MDA for the three experiments. Some of the most important species in the prediction process are from the *Veillonella*, *Haemophilus* and *Streptococcus* genera (see paragraph below on biological interpretation for more information). Noteworthy, the MDA may be different from one algorithm to another and can reach up to 0.3 points of the models’ accuracy. MDA, is computed for each feature found in the significantly best models for each training set in the cross-validation. In **Figure S9** *right* we illustrate the composition of each model (in column) with the corresponding coefficients {-1, 0, 1} coloured {blue, white, red} respectively. Models from the three FBM are simultaneously ordered by model-size and accuracy (in the columns). We can notice that the top most important features are also some of the most prevalent in the FBM.

Moreover, we display in **Figure S10** *right* the abundance of each of the above-mentioned features for each of the predicted class as boxplots. A grey star depicts a significant difference by a Mann-Whitney test. We notice that most of these features display differential abundance. For instance, the most important feature *Veillonella unclassified* is very abundant in the disease group (blue). Finally, **Figure S10** *middle* depicts the prevalence of the features in the global dataset (grey bars) and also inside each class (red and blue dots). Similarly, a grey star indicates a significant difference in prevalence between the healthy and disease classes as tested by a Pearson's Chi-squared Test. Noteworthy, most top-mda (important) features are also those with the most of significantly different prevalence and abundance features.

**
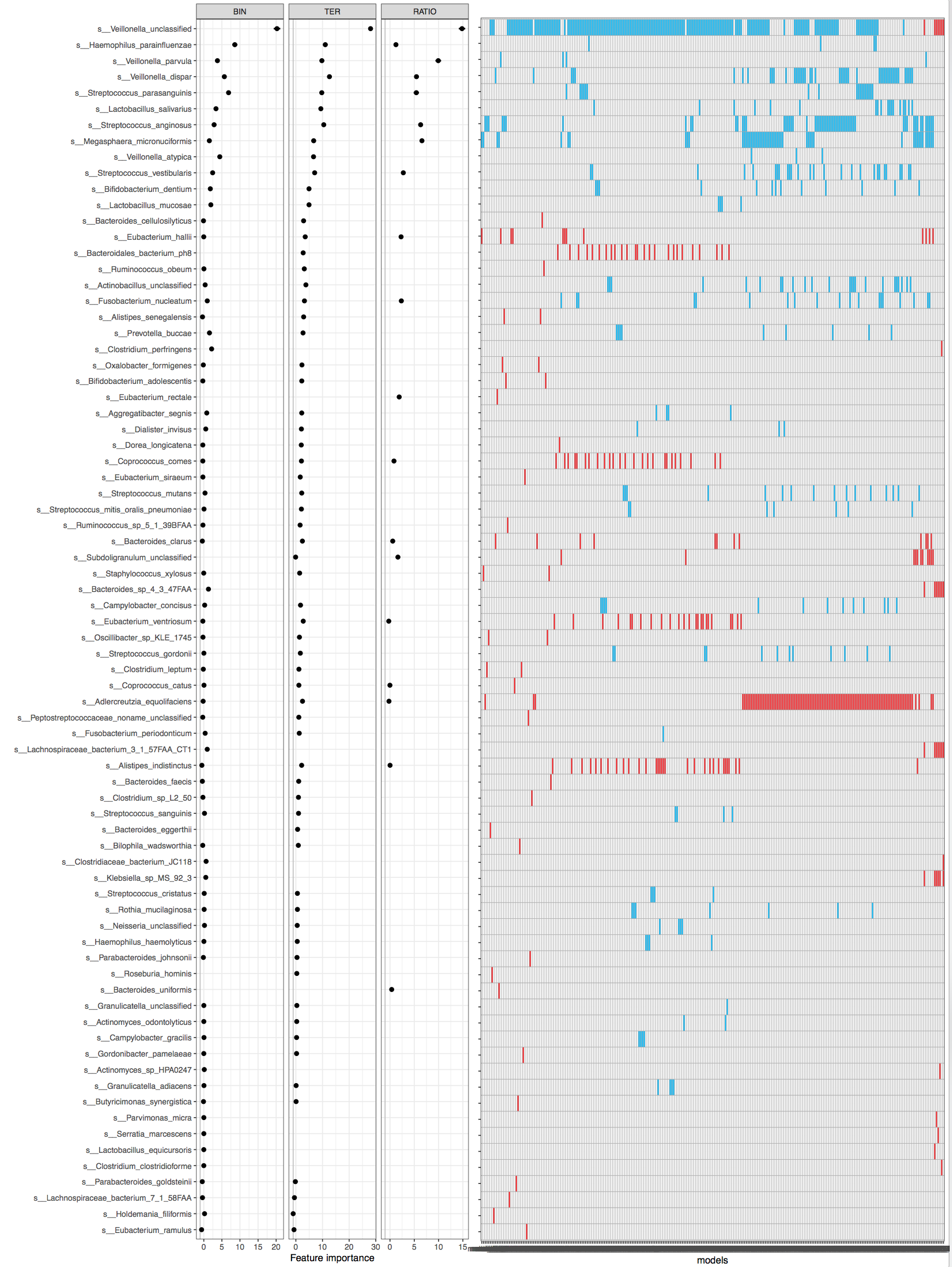
**

**Figure S9: Feature composition and feature importance of the Family of Best Models for the Cirrhosis Stage 1 dataset**

*Left*: The importance (MDA * 100) of the FBM models for Bin, Ter and Ratio in the whole Cirrhosis stage-1 (species) dataset. Features (rows) are ordered by their average MDA over the three experiments. *Right*: Each column represents a BTR model. The coefficients {-1, 0, 1} of each model are coloured {blue, white, red} respectively are ordered simultaneously by model-size and accuracy.


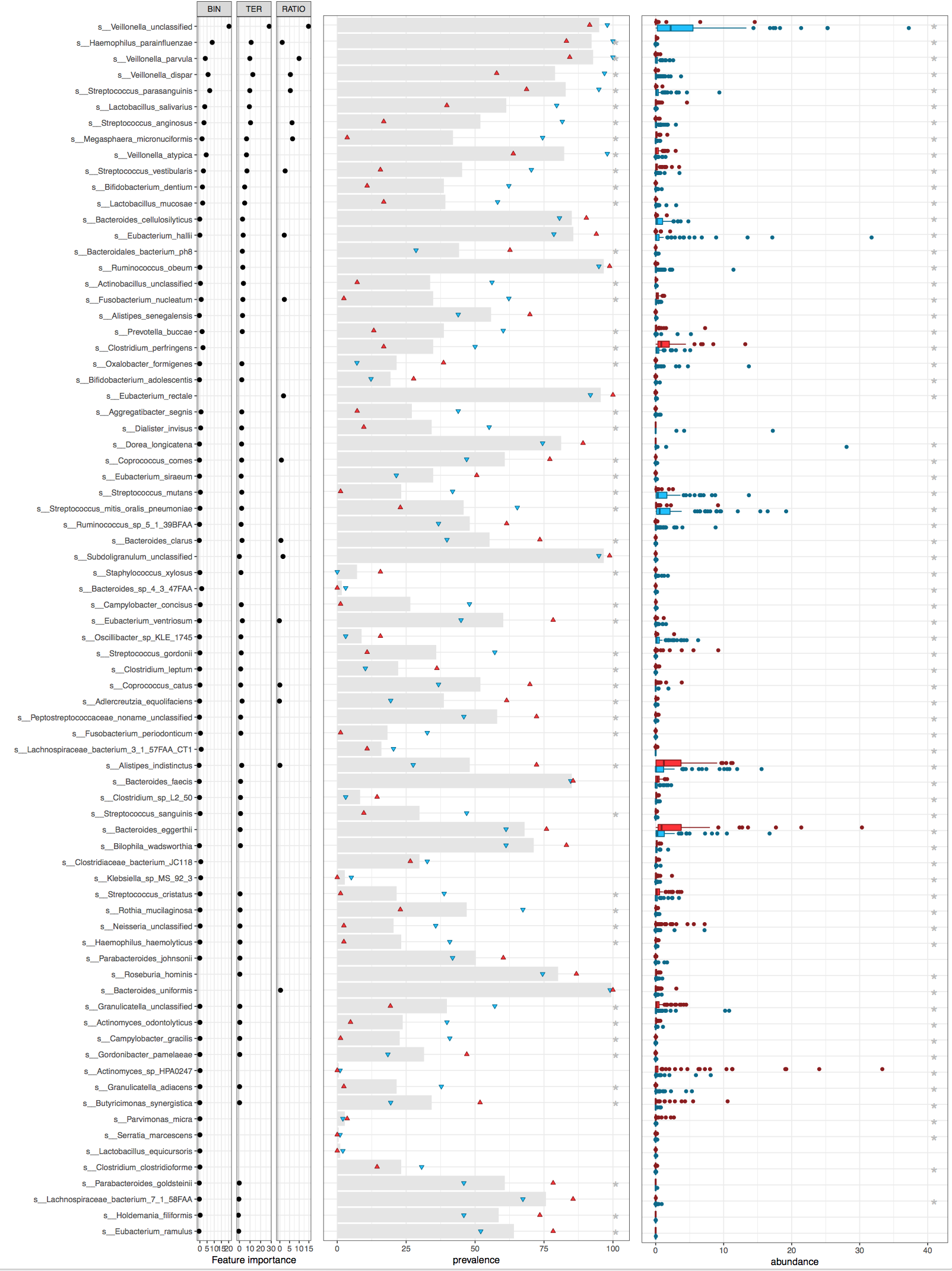


**Figure S10**: **Feature abundance and prevalence of the Family of Best Models for the Cirrhosis Stage-1 dataset**

*Left*: The importance (MDA * 100) of the FBM models for Bin, Ter and Ratio in the whole Cirrhosis stage-1 (species) dataset. Features (rows) are ordered by the average MDA in the three experiments. *Middle*: The prevalence of each feature for the whole dataset (grey bar) and in the prediction classes (disease, healthy) depicted as blue and red dots respectively. Grey stars indicate significant difference. *Right*: The abundance of each feature in the prediction classes (disease, healthy) depicted as blue and red box plots respectively. Grey stars indicate significant difference.

BTR models are simple, accurate and rely on features, which are simultaneously selected by multiple FBM models. Most of these features change significantly both in abundance and prevalence between the classes to predict. If we have provided an estimate of the predictive importance of the features in a similar way to RF, it is more difficult to propose an aggregation operator (such as *max* or *average*) that would compute a *model importance*.

Next, we compared the feature importance (FI) of the BTR models with the FI computed using random the RF, as well as SVM, ENET with the help of a non-native approach (R package *rminer v. 1.4.2*). We also computed p-values of Mann-Whitney tests – a largely used approach in the field for selecting important features. As depicted in **Figure S11**, the FI of the three BTR models (in the Cirrhosis-2 species dataset) correlates strongly (bin correlates at 0.76 and 0.83 respectively with ter and ratio, using Pearson correlation). It also correlates strongly with the FI quantified with RF (0.68, 0.81, 0.7, respectively with bin, ter and ratio). However, the FI of SVM models is quite different and does not correlate well with the above. FI of ENET models correlates on the other end better (0.3, 0.4, 0.15 0.4 and 0.29 respectively with bin, ter, ratio, RF and SVM) than SVM and also correlates with SVM itself (0.29). The correlation with the p-value of the statistical tests is negative (a smaller p-value is more significant and its importance is higher). However, these correlations are small as the p-value distribution is not normal and the relation not-linear. When the correlations are computed for each of the modalities of the “status” variable, we notice that correlations are stronger for the modality: status = -1 (blue points depicting features enriched in the patient group). This indicates that the most important features are enriched in patients as discussed below).


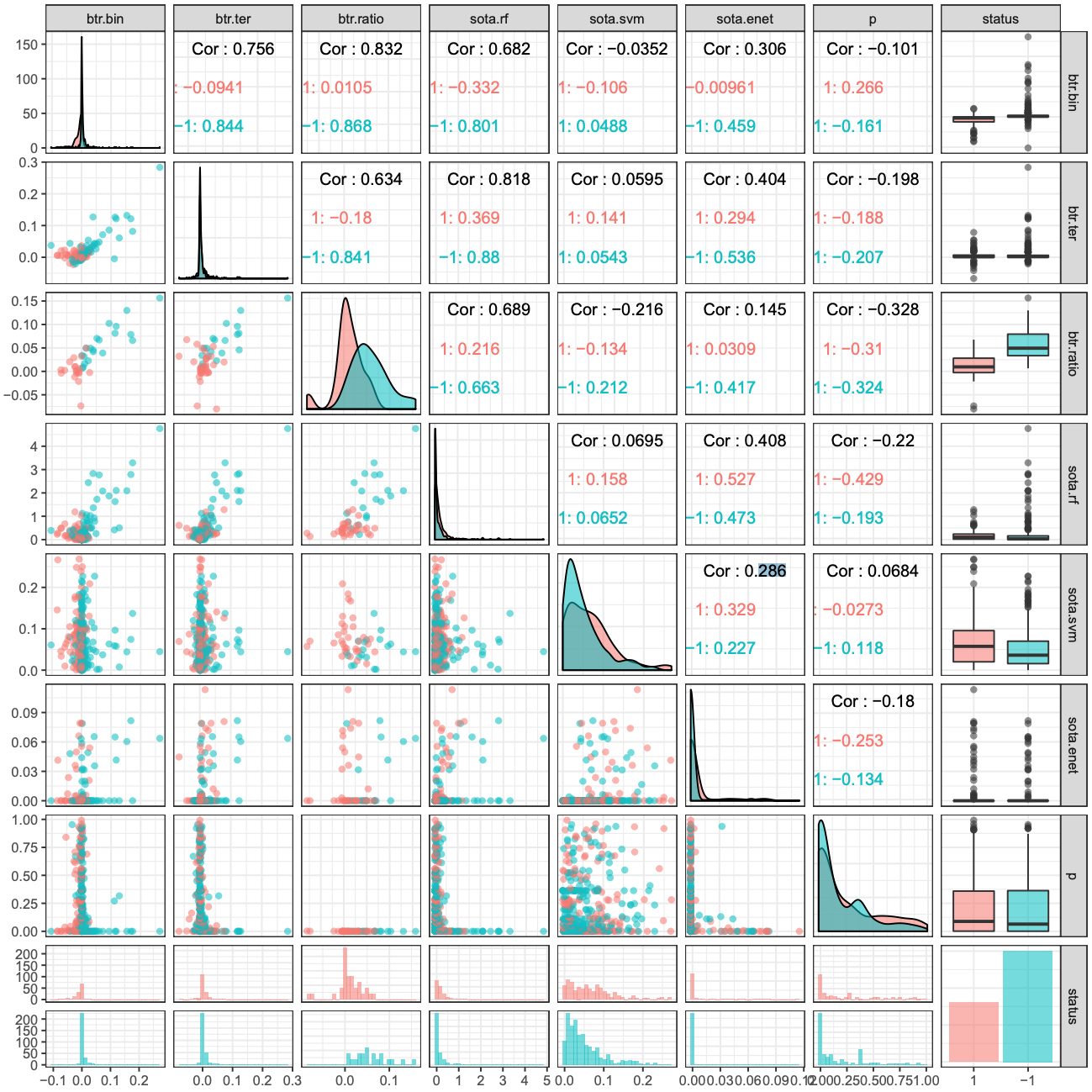


**Figure S11: Comparison of feature importance between BTR models and RF as well as statistical ranking.**

Pairwise comparison of the feature importance identified by different models. Figures are annotated by the enrichment of the variables as -1 (blue) and 1 (red) being respectively more abundant in patients and controls in the Cirrhosis-1 (species) dataset. Besides the histograms, boxplots and density plots are shown.

Next, we focused on the scatter-plot of the FI from the Ter models vs. RF (see **Figure S12**) to explore the most important features and noticed that they are very similar and largely discussed in the main text. These analyses show that FI in the BTR models (which are extremely simple) correlates well with that of well-established (but more complex) methods such as RF.


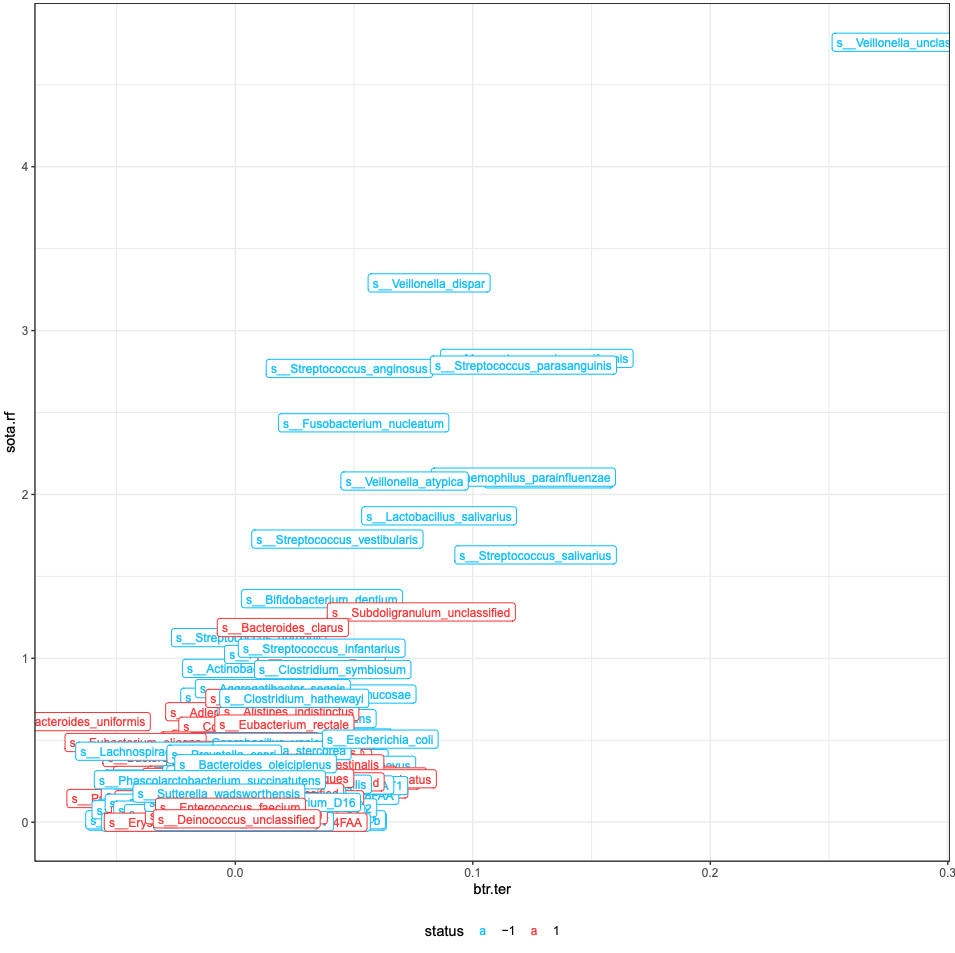


**Figure S12: Comparison of feature importance between TER models and RF**

The names of the features are placed according to their feature importance for TER and RF respectively in the x and y axis. Colors (blue and red) indicate enrichment respectively in patients and controls.

We also introduced PDA, a second concept of importance, based on the mean prevalence in FBM models (i.e. percentage of times a given feature is selected in a model composing the FBM). We compute the average model prevalence for each fold during the cross-validation process and finally, propose the CV-averaged PDA along with the standard error of the mean as measure of feature importance. Altogether, such ranking of the features supports establishing confidence on the predictive subset of the microbiome features as well as on the subsequent models discovered by *predomics*.

Finally, focusing on the set of best models is useful when trying to characterize the ecosystemic differences of the different classes, or understanding potential relationships between species. Integrating such models with other data such as phylogeny or functional annotations, may help to better understand bacterial communities, their interaction and their role in the ecosystem and host. As a proof of concept, we attempted such analysis using a network reconstruction approach on the co-presence of features in the *predomics* BTR models (see **Figure 5** in the main text).

### BTR models provide relevant biological interpretation in liver cirrhosis

In the original liver cirrhosis (LC) study, major dysbiosis was observed in LC patients characterized by decreased microbial richness with a depletion of commensal bacteria and an invasion of oral bacteria in the gut [11]. Several markers at taxonomic and functional levels were identified to be associated with the disease as identified by univariate test of abundance features. The direct interaction of liver with gut through the hepatic portal and bile acid secretions probably makes enteric dysbiosis a critical contributor in the disease progression and may involve translocation of bacteria and their derived-products through disrupted gut epithelium ^37^.

We first explored BTR models based on abundance data of taxonomic feature (see online methods), going from broad (phylum) to specific (species) taxonomic levels (**Figure 2B**). At the phylum level, the Ratio model *(S6)* points to a mutual exclusion between Bacteroidetes and the combination of Proteobacteria and Viruses, with Bacteroidetes associated to the control group and Proteobacteria and Viruses associated to the diseased group. The combination of Proteobacteria and Viruses is also picked up in the best Bin model *(S4)*. The composition of these models is in line with a significant decrease in Bacteroides and the significant increase in Proteobacteria and Fusobacteria in the liver cirrhosis group reported in the reference paper, with Fusobacteria which appears in the FBM associated to the liver cirrhosis status. The decrease in Bacteroidetes can be interpreted as a signature of decrease in highly prevalent gut bacteria, whereas the increase of Proteobacteria has been repeatedly reported in dysbiotic microbiomes of patients with liver cirrhosis, intestinal diseases and type-2 diabetes. It has also been associated with chronic inflammation in link with serum lipopolysaccharides [33, 34]. The Virus phylum was not previously identified, which could be explained by the reference database used in the original study. However, in the current study, it is observed to be more prevalent and abundant in the liver cirrhosis group. This may reflect the oral microbiome signature and/or reflect an increased incidence of viral infections together with opportunistic pathogens.

*(S4) p__Proteobacteria + p__Viruses_noname < 4.5* ***then*** *class = healthy*

*(S5) p__Bacteroidetes + p__Euryarchaeota - p__Viruses_noname > 50****then*** *class = healthy*

*(S6) p__Bacteroidetes / (p__Proteobacteria + p__Viruses_noname) > 11****then*** *class = healthy*

The potential competition between oral and gut microbes in the progression to cirrhosis reported in previous studies [35] is reflected in best by Ter and Ratio models with genus abundance data, that combine *Veillonella* (oral bacteria; opportunistic pathogen) enriched in liver cirrhosis patients at one side and *Bacteroides* plus *Eubacterium* *(S9)* or *Coprococcus* *(S8)* enriched in controls, which represents well known butyrate producers (*Coprococcus* and *Eubacterium*) and complex polysaccharide degraders (*Bacteroides* genus) of the gut microbiota [36]. *Veillonella* is also included in the best Bin model *(S7)* together with the viral genus *Podoviridae* (in line with best models with phylum data) and *Anaerostipes* (Firmicutes) in patients. Among the most important genera in the FBM we find *g__Veillonella*, *g__Streptococcus*, *g__Haemophilus*, *g__Coprococcus* and *g__Lactobacillus*, all more abundant/prevalent in the liver cirrhosis patients.

*(S7) g__Veillonella < 0.27* ***then*** *class = healthy*

*(S8) g__Veillonella - g__Coprococcus < 0.1* ***then*** *class = healthy*

*(S9) (g__Eubacterium + g__Bacteroides) / g__Veillonella > 140* ***then*** *class = healthy*

The species abundance results provide in agreement with literature additional detailed information. Best Ratio and Ter models (1,2,3 in online methods), include oral bacterial species of the genus *Veillonella (Veillonella_unclassified), Streptococcus (S. parasanguinis and S. anginosus)* and opportunistic pathogens like *Megasphaera micronuciformis* that proliferate in liver cirrhosis patients, whereas butyrate producers of the genus *Subdoligranilum* (*Subdoligranilum unclassified*) closely related to the well-known species *Faecalibacterium prausnitzii [37]* species and complex polysaccharides degrading species like *Bacteroides cellulosilyticus [38]* characterize control subjects. *Megasphaera micronuciformis* was also associated with primary biliary cirrhosis based on 16S rRNA [39].

A more in-depth exploration of the FBM (**Figure S5, S8, S9**) and the feature-model co-occurrence network (**Figure 4**) indicates the selection of not only differentially abundant species but also redundant ones that are similar in phylogeny and eventually function. Noteworthy, this network built solely using model co-presence information resembles the network from the original study constructed with presence/absence species data for each metagenome. BTR models explored jointly provide relevant biological information.

At the functional level, predictive models *(S10-S12)* from MetaCyc pathway abundance data include pathways that suggest an increased aerobic metabolism (HEMESYN2−PWY: heme biosynthesis II (anaerobic), essential for cytochromes and heme-containing globins, PWY−922: mevalonate pathway I, needed for the biosynthesis of ubiquinone and menaquinone complexes of respiratory chains). Interestingly, increase in aerobic respiration profiles has also been identified as metabolic signatures of inflammation-associated dysbiosis in models of colitis [40]. Moreover, we observe the presence of modules related with bacterial peptidoglycan biosynthesis in the FBM (PWY−6470: peptidoglycan biosynthesis V), which has been described as an elicitor of inflammatory response associated to the progression of liver cirrhosis [41], in agreement with a more inflammatory profile of cirrhotic patients.

*(S10) HEMESYN2-PWY: heme biosynthesis II (anaerobic) + PWY-922: mevalonate pathway I < 3.1e-05* ***then*** *class = healthy*

*(S11) CENTFERM-PWY: pyruvate fermentation to butanoate -
HEMESYN2-PWY: heme biosynthesis II (anaerobic) > -4.5e-06* ***then*** *class = healthy*

*(S12) (PWY-7219: adenosine ribonucleotides de novo biosynthesis + PWY-6121: 5-aminoimidazole ribonucleotide biosynthesis I+ HISDEG-PWY: L-histidine degradation I) / HEMESYN2-PWY: heme biosynthesis II (anaerobic) > 45* ***then*** *class = healthy*

Other authors have proposed a ratio index based on literature review, named Cirrhosis Dysbiosis Ratio (CDR) [32]. Features used in the CDR index correspond to abundances at the Family taxonomic level. In the Qin et al dataset [11], used here, there are three groups associated to the *Clostridiaceae* family (f_Clostridiaceae, f_Clostridiales_noname and f_Clostridiales_Family_XI_Incertae_Sedis). Based on their description we have constructed 3 models with features from the family taxonomic level.

*(S13) (f__Ruminococcaceae + f__Lachnospiraceae + f__Clostridiales_Family_XI_Incertae_Sedis) / (f__Bacteroidaceae + f__Enterobacteriaceae) > 0.83* ***then*** *class = healthy*

*(S14) (f__Ruminococcaceae + f__Lachnospiraceae + f__Clostridiales_noname) /
(f__Bacteroidaceae + f__Enterobacteriaceae) > 0.83* ***then*** *class = healthy*

*(S15) (f__Ruminococcaceae + f__Lachnospiraceae + f__Clostridiaceae) /
(f__Bacteroidaceae + f__Enterobacteriaceae) < 0.065* ***then*** *class = 1*

The performance of these models (*S13-S15*; **Figure 6B-C**) is low in accuracy (0.56, 0.56, 0.55, respectively; **Figure 6F**). Such performance could be explained by the inclusion in the CDR model of the *Bacteroidaceae* family as enriched the liver cirrhosis group together with *Enterobacteriaceae*. However, we observe the opposite association in the Qin et al WGS dataset where Bacteroidetes-related taxonomic features are enriched in controls and this is consistent for different taxonomic levels (**Figure 6E**).

On the contrary, the Ratio model (*S16*; **Figure 6A**) discovered with *predomics* provides high accuracy (acc = 0.86; **Figure 6F**). This model includes the *Actinobacteria* family *Coriobacteriaceae* enriched in controls and as well as the combined abundance of *Proteobacteria* lineages reflecting potential opportunistic pathogens (*Pasteurellaceae* and *Campylobacteraceae*) along with the *Streptococcaceae* family enriched in the gut microbiome of liver cirrhosis [11] (**Figure 6E**). This model seems to better reflect the overall changes described above.

*(S16) f__Coriobacteriaceae /
 (f__Pasteurellaceae + f__Streptococcaceae + f__Campylobacteraceae) > 0.17****then*** *class = healthy*

Literature-based interpretation of the models can be quite important to build confidence in them. Nevertheless, the microbiome field being still in its early stages it is quite difficult to construct manually curated models that generalize well. The *predomics* approach fulfils this need as illustrated by this example. Another important point to consider is the reference source of functional features used for quantitative metagenomic analysis in the sense of what we want to quantify in the context of the ecosystem under study. We observe this with the cirrhosis datasets for which we have abundance of functional features of the MetaCyc pathway space, which cover the entire knowledge of metabolic pathways from all domains of life [49]. This makes that we have abundance data for metabolic pathways not supposed to be present in human gut ecosystem like photosynthesis-related pathways, which appears among the populations of best models. In this context, more targeted approaches to quantify functional features relevant to the ecosystem under study like GMM’s [50] will be more suitable for automatic reconstruction of predictive models from quantitative metagenomic data of human gut microbiome.

Altogether, these results indicate that BTR models discover and embed important features with relevant biological information. These features are usually correlated with the class and some are complementary and others are redundant. Finally, they are far more accurate then literature based and manually curated ones.

### Models of bariatric surgery-induced metabolic improvements based on BTR regression

In addition to classification, *predomics* can perform regression tasks by searching models that correlate with the quantitative variable to predict (see methods). We used data from a recently published study where obese patients underwent Roux-en-Y gastric bypass (RYGB; n=14) and adjustable gastric band (AGB; n=10) surgery [26]. Patients’ metagenomes were measured pre-surgery and twelve months post-surgery (among others). Most patients who underwent the surgery improved their body weight, body composition and glucose homeostasis (glycemia, insulinemia and glycated haemoglobin (*i.e.* HbA1C)) with significant variation between individuals. Metabolic improvement was measured as the relative change at 12 months compared to baseline.

We searched pre-surgery metagenomic data for bacteria that could predict the improvement of BMI, trunk fat distribution, and HbA1C and discovered models composed of six, four, and three species reaching R^2^ values of 0.53, 0.62, 0.52 respectively (**Figure S13**). The algorithm generalizes well when tested in cross validation (20-times 5-fold CV), although we observe decreasing performance in testing sets likely due to the small sample size.

Interestingly, the models highlight bacterial species such as *Faecalibacterium prausnitzi, B. pseudocatenulatum and P. goldsteni*, which were previously shown to be associated with metabolic health and low-grade inflammation. Probably the relevant example is *Faecalibacterium prausnitzii*, an abundant species in the gut microbiome, depleted in patients with Crohn’s disease, ulcerative colitis and type 2 diabetes, is known to have anti-inflammatory properties associated with butyrate production [10, 51, 52]. Also, *B. pseudocatenulatum* and *P. goldsteni* have been associated with improved inflammatory profile in obesity [53, 54]. Decrease in the abundance of *Intestinibacter bartleii*, a close relative of Clostridium species present in the predictive model of glycated haemoglobin improvement, has been associated with patients under metformin treatment across different countries [15]. Altogether, these models suggest that the presence of common gut bacteria commensals associated with a healthy microbiome at baseline is a relevant signature associated with metabolic improvements and bariatric-surgery-induced weight loss in severe obesity. Nevertheless, the sample size is relatively small and these results constitute only a proof of concept. While this is a proof of concept, these results illustrate the power of the microbiome to predict change in body composition and glucose homeostasis.


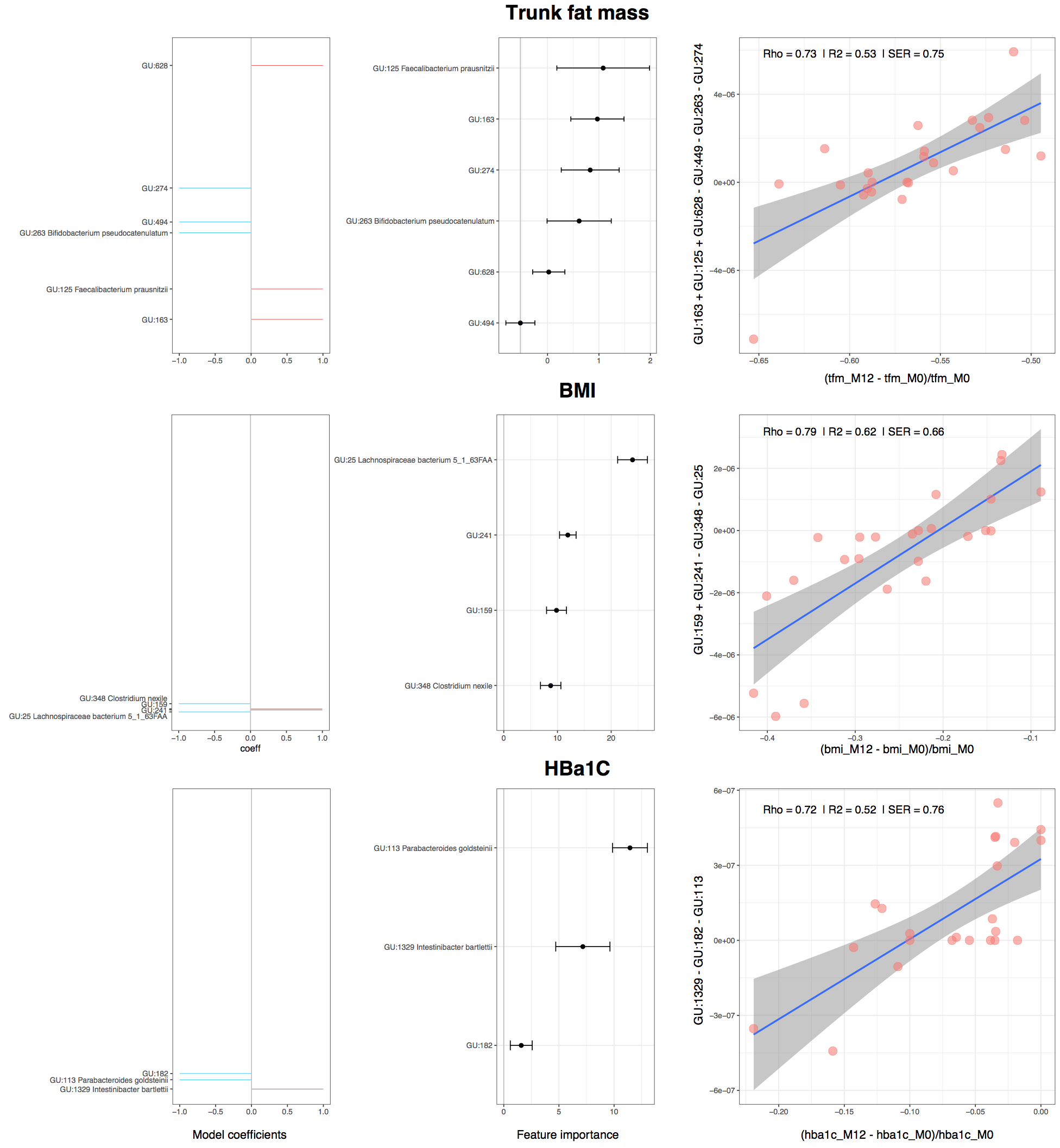


**Figure S13**: **Quantitative prediction of phenotypic outcome after bypass surgery.**

*Left*: Barcode plots indicating the coefficients of the ternary models. *Middle*: Percentage of mean decrease R^2^, measuring the importance of features in the fitting objective during the cross-validation process. *Right*: Scatter plots indicating the fitting of the model score (y-axis) as measured with baseline microbial profiles against the relative change of each of the three phenotypes (trunk fat mass, BMI and HbA1C) in the x-axis.

### Comparing TerLog models with geometric mean balances

Recently Rivera-Pinto *et al.* introduced a balance based approach to tackle compositionality in the context of prediction [30]. Independently, we proposed here more general models that encompass this latter concept of balance as a particular case of our Ter models when applied to log-transformed unnormalized data. Indeed, an additive model based on logged data corresponds to multiplicative models on the abundance where positive values are at the numerator and negative ones are at the denominator. The balance model introduced by Rivera-Pinto *et al.* is proportional to the difference between the means of the log-transformed abundance of two groups of variables.

Besides the compositionality-associated issues, balance and TerLog models embed the *multiplicative* relationship between species of a same balance side (sum of logged data). With our Ratio model, we have proposed yet another concept, since when applied to non-log-transformed data the *cumulative* relationship between species of a same balance side is discovered. In this case the models are robust to the compositionality problem because it is a ratio-based model.

To compare both the balance approach and the TerLog approach, we applied the *selbal* algorithm to the Cirrhosis stage-1 (species) dataset using raw count data, which were log-transformed after adding a pseudocount as proposed in their method. Next, we applied *predomics* to the same dataset and searched for Ter models. We explored the overall capacity of the methods to select comparable predictive signatures. To get in line with the strategy used in *selbal,* i.e. count the number of times a feature is found in the balance of each cross-validation step, we computed the mean prevalence of the features in the FBM of each cross-validation step (see online methods). Even though not identical as the algorithms and models are different, the results display an impressive overlap (**Figure S14A,B**).

Next, we used the features from the best balance discovered by *selbal*, which consisted of 22 features (7 numerator and 15 denominator) and built the corresponding Ter model by adding 1 and -1 coefficient to the numerator and denominator respectively. The model score evaluated was correlated to that from the selbal balance (R = 0.97; **Figure S14C**), indicating a strong similarity.

Finally, we compared the overall generalization performance of the algorithms in terms of AUC in the test datasets of the CV approach. The cross-validation process was performed similarly for both experiments, as a 10-times 10-fold process. The results depicted in Figure S10D indicate a difference in performance (p<0.0001, Mann-Whitney), favouring *predomics*. Noteworthy, the current implementation of the greedy stepwise algorithm used in *selbal*, made it difficult to run it to larger datasets, while the *predomics* implementation easily scale up to large datasets like state-of-the-art approaches RF, SVM or Elastic Nets do.

**
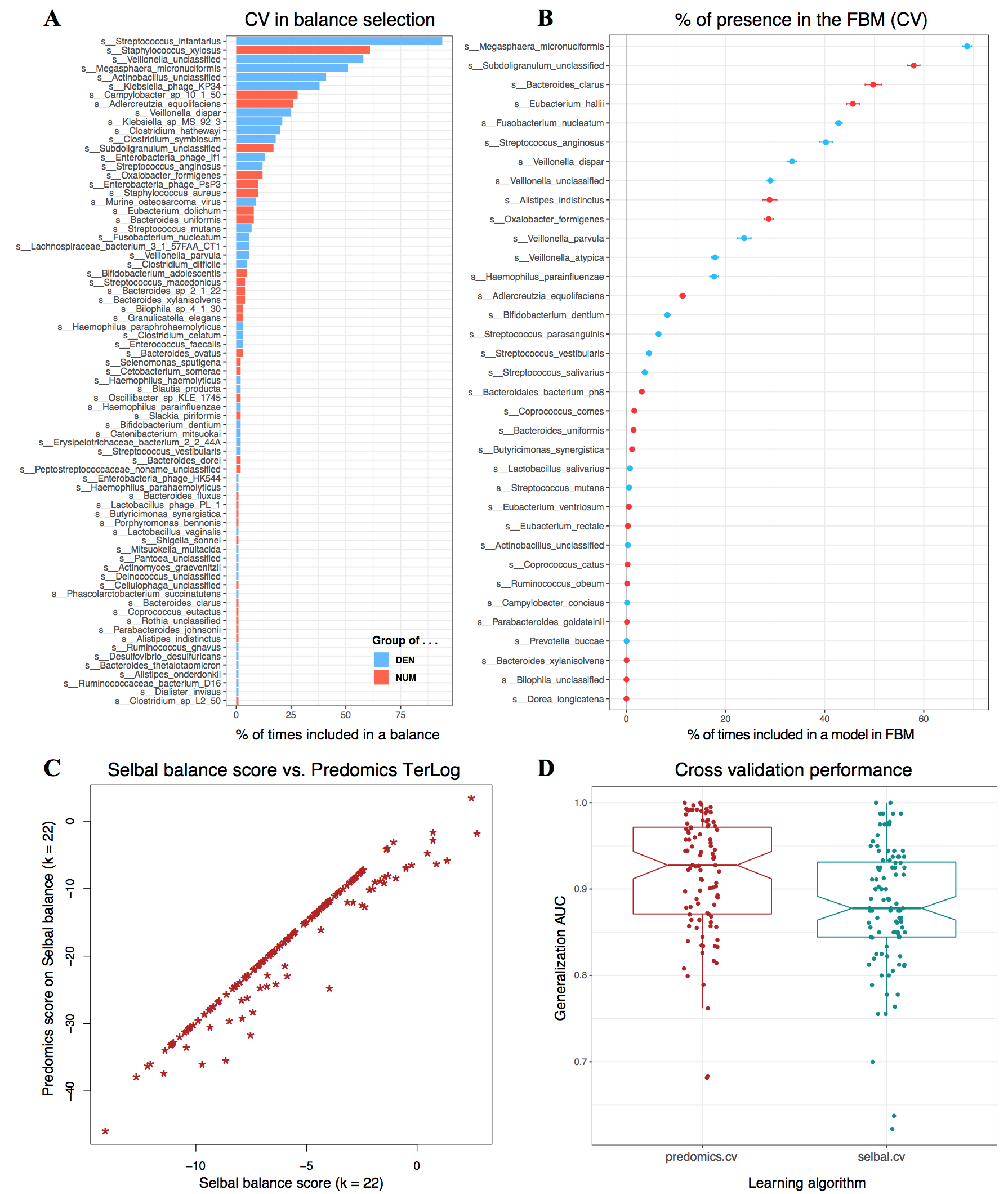
**

**Figure S14**: **Comparison of *selbal* balances with TerLog *predomics* models**

**A**: Feature selection in balances during the CV process as produced by the *selbal* algorithm. The figure was formatted for an integrated presentation. **B**: Average percentage of feature selection in the BFM discovered at each training step in the CV process. Standard error of the mean is shown. The blue and red colours indicate respectively the coefficients -1 and 1 in the Ter model, which correspond to the denominator and Numerator of the *selbal* balances. **C**: Scatter-plot of the scores for each observation computed with both models generated by *predomics* and *selbal* using the same set of 22 features from the best balance. **D**: Boxplots representing the distribution of AUC (area under the curve) of the best models discovered in the test sets of each fold of the CV-process.

**References**

48. Karlsson FH, Tremaroli V, Nookaew I, Bergström G, Behre CJ, Fagerberg B, et al. Gut metagenome in European women with normal, impaired and diabetic glucose control. Nature. 2013;498:99.

49. Caspi R, Billington R, Ferrer L, Foerster H, Fulcher CA, Keseler IM, et al. The MetaCyc database of metabolic pathways and enzymes and the BioCyc collection of pathway/genome databases. Nucleic Acids Res. 2016;44 D1:D471-80. doi:10.1093/nar/gkv1164.

50. Darzi Y, Falony G, Vieira-Silva S and Raes J. Towards biome-specific analysis of meta-omics data. 2015;10 5:1025-8. doi:10.1038/ismej.2015.188.

51. Miquel S, Martin R, Rossi O, Bermudez-Humaran LG, Chatel JM, Sokol H, et al. Faecalibacterium prausnitzii and human intestinal health. Curr Opin Microbiol. 2013;16 3:255-61. doi:10.1016/j.mib.2013.06.003.

52. Walker AW, Ince J, Duncan SH, Webster LM, Holtrop G, Ze X, et al. Dominant and diet-responsive groups of bacteria within the human colonic microbiota. The ISME Journal. 2010;1:25. doi:10.1073/pnas.0812600106.

53. Moya-Perez A, Neef A and Sanz Y. Bifidobacterium pseudocatenulatum CECT 7765 Reduces Obesity-Associated Inflammation by Restoring the Lymphocyte-Macrophage Balance and Gut Microbiota Structure in High-Fat Diet-Fed Mice. PLoS One. 2015;10 7:e0126976. doi:10.1371/journal.pone.0126976.

54. Wu T-R, Lin C-S, Chang C-J, Lin T-L, Martel J, Ko Y-F, et al. Gut commensal Parabacteroides goldsteinii plays a predominant role in the anti-obesity effects of polysaccharides isolated from Hirsutella sinensis. Gut. 2019;68 2:248. doi:10.1136/gutjnl-2017-315458.
