# Supplementary material for: Interpretable and accurate prediction models for metagenomics data
Source: Gigascience. 2020 Mar 9;9(3):giaa010. doi: 10.1093/gigascience/giaa010 (PMC7062144; doi:10.1093/gigascience/giaa010)
Supplement: giaa010_Supplemental_Files [file giaa010_supplemental_files.zip › Supplementary_package_predomics_address.pdf]

The supplementary package *predomics* is available for download at the following address
http://integromics.fr/~eprifti/supplementary_package_predomics.zip
