# Supplementary material for: Interpretable and accurate prediction models for metagenomics data
Source: Gigascience. 2020 Mar 9;9(3):giaa010. doi: 10.1093/gigascience/giaa010 (PMC7062144; doi:10.1093/gigascience/giaa010)
Supplement: giaa010_Response_to_Reviewer_Comments_Original_Submission [file giaa010_response_to_reviewer_comments_original_submission.pdf]

Author's Response To Reviewer Comments

Close

Dear Dr. Nogoy,

Please accept our gratitude in allowing our work to be reviewed in Gigascience and accompanying us during this process.

We have carefully examined both reviews and have tried our best to answer them point-by-point and in a constructive manner. We have performed additional analyses to answer some of the questions raised by the reviewers and improved upon their advice both the revised version of the manuscript as well as the supplementary material.

Moreover, we registered the predomics software in the scicrunch.org repository as requested under the accession number RRID: SCR_017415 and mentioned it in the revised version of the manuscript (page 14).

We hope that both the reviewers and the editorial board will find our answers and the new version of the manuscript satisfying.

On a more general note, we would like to stress that we particularly appreciate the principles to which the journal Gigascience adheres and that they guided us in the choice to submit a manuscript.

With our best regards
Edi Prifti and Jean-Daniel Zucker

PS: As the detailed responses to the reviewers including additional figures and analyses are provided as a supplementary document uploaded with the revised manuscrit.
The file is called "Predomics_Cover_letter_And_Response_To_Reviewers.docx"

Close
