# Supplementary material for: Interpretable and accurate prediction models for metagenomics data
Source: Gigascience. 2020 Mar 9;9(3):giaa010. doi: 10.1093/gigascience/giaa010 (PMC7062144; doi:10.1093/gigascience/giaa010)
Supplement: giaa010_Response_to_Reviewer_Comments_Revision_1 [file giaa010_response_to_reviewer_comments_revision_1.pdf]

Author's Response To Reviewer Comments

Close

Please find all answers to reviewers (which include graphics) as well as letter to editors in the attached document named Predomics_Cover_letter_And_Response_To_Reviewers_2_review.

Close
