# Supplementary material for: Interpretable and accurate prediction models for metagenomics data
Source: Gigascience. 2020 Mar 9;9(3):giaa010. doi: 10.1093/gigascience/giaa010 (PMC7062144; doi:10.1093/gigascience/giaa010)
Supplement: giaa010_Response_to_Reviewer_Comments_Revision_2 [file giaa010_response_to_reviewer_comments_revision_2.pdf]

Author's Response To Reviewer Comments

Close

Dear Dr. Nogoy,

Please accept our warm gratitude in allowing our work to be reviewed in Gigascience and accompanying us during this process.

We have carefully examined the review of Reviewer 1 and have answered them point-by-point. In particular we have carefully worked on first the wording of the abstract conclusion and second clarified the main messages of the whole discussion/conclusion section. We believe this provides clarification on the message. We also added two references as requested by the reviewer.

The other comments of the reviewer were based on fulfilling the recommendation of GigaScience regarding the paper and reference formatting. Based on a close look on research guidelines from GigaScience website https://academic.oup.com/gigascience/pages/research, we have carefully (re)formatted the document accordingly including the references that now strictly follow the Gigascience style.

We now believe we answered appropriately to the reviewer main comment (i.e. conclusion wording) and we look forward to your prompt response regarding our paper, that we believe has improved greatly.

Looking forward to your feed-back and with our best regards
Edi Prifti and Jean-Daniel Zucker

Reviewer reports:
Reviewer #1
Reviewer #1:
1. "Consequently, most models tend to overfit the training data and result in predictions arising from random sampling fluctuations." Please cite the literatures to support your view.
We added two additional and appropriate references which support our view. The references are the following :
• Zhou Y-H and Gallins P. A Review and Tutorial of Machine Learning Methods for Microbiome Host Trait Prediction. Frontiers in genetics. 2019;10:1-14. doi:10.3389/fgene.2019.00579.
• Topçuoğlu BD, Lesniak NA, Ruffin M, Wiens J and Schloss PD. Effective application of machine learning to microbiome-based classification problems. bioRxiv. 2019:816090. doi:10.1101/816090.

We added them in the full text after the sentence mentioned by the reviewer.

2. The paper should be carefully checked, such as lacing full stop at the end of the paragraph. Also found this phenome in Figure legends.
We reviewed the whole document for punctuation and other inconsistencies and have done our best to fix them.

3. The structure of the article is inconsistent, please format it.
We followed in detail the research guidelines from GigaScience website https://academic.oup.com/gigascience/pages/research and have formatted the document accordingly.

4. The unformatted font.
We followed in detail the research guidelines from GigaScience website https://academic.oup.com/gigascience/pages/research and have formatted the document accordingly.

5. The conclusion is abstract and unclear.

We have revised the whole discussion/conclusion section and clarified the main messages.
We have also revised the conclusion in the abstract. It now reads “Predomics is a new algorithm that helps in providing reliable and trustworthy diagnostic decisions in the microbiome field. Predomics agrees with societal and legal requirements that plead for an explainable Artificial Intelligence approach in the medical field.”.

6. Mixed use of full names and abbreviations of Journal in Reference, what's more some have Vol, and some don't, such as:

"5.Walsh, C.J., et al., Beneficial modulation of the gut microbiota. FEBS Letters, 2014. 588(22): p. 4120-4130.
6. Ley, R.E., et al., Obesity alters gut microbial ecology. Proceedings of the National Academy of Sciences, 2005. 102(31): p. 11070.
7. Qin, N., et al., Alterations of the human gut microbiome in liver cirrhosis. Nature, 2014. 513: p. 59.
8. Wen, L., et al., Innate immunity and intestinal microbiota in the development of Type1 diabetes. Nature, 2008. 455(7216): p. 1109-1113.
9. Qin, J., et al., A metagenome-wide association study of gut microbiota in type 2 diabetes. Nature, 2012. 490: p. 55."

We followed in detail the research guidelines from GigaScience website https://academic.oup.com/gigascience/pages/research and have formatted both the references and the document accordingly. We used the Gigascience Endnote style. Nevertheless, please note that one article does not have a volume.

Close
