# Supplementary material for: Interpretable and accurate prediction models for metagenomics data
Source: Gigascience. 2020 Mar 9;9(3):giaa010. doi: 10.1093/gigascience/giaa010 (PMC7062144; doi:10.1093/gigascience/giaa010)
Supplement: giaa010_Reviewer_1_Report_Original_Submission — Kang Ning -- 6/27/2019 Reviewed [file giaa010_reviewer_1_report_original_submission.pdf]

Reviewer Report

Title: Interpretable and accurate prediction models for metagenomics data

Version: Original Submission **Date:** 6/27/2019

Reviewer name: Kang Ning

Reviewer Comments to Author:

In this paper, the author provided a machine learning approach named Predomics, aiming to conduct microbiome biomarker discovery. In comparison to popular machine learning methods including support vector machines and elastic-net regularization, it was based on simple biological principles which meant it was interpretable. With experiment on 109 datasets, Predomics achieved comparable accuracy with state-of-art methods like SVM in some conditions. Besides feature extracting, author further constructed feature co-presence network in models to show the biological interpretation behind selected features.

Major concern about the result:

(1)It could be observed from figure 2 that in the training set, the accuracy of Predomics fluctuated a lot as the model size grew. In the paper, author regarded it represented Predomics was able to avoid overtraining. However, the fluctuation or decrease in accuracy was much too severe in large scale model. For example, the accuracy of TER in Predomics droped to 75% in k_24. I wonder whether it means that Predomics method are not capable of predicting in large scale model.

(2)When comparing Predomcis and SOTA methods, author provided a normalized coefficition to reflect the interpretability of Predomics' results. But when author used mean decrease accuracy plots to indicate feature, the importance score of SVM and ENET was missed. I wonder the importance score comparison between these SOTA methods and Predomics.

Methods

Are the methods appropriate to the aims of the study, are they well described, and are necessary controls included? Choose an item.

Conclusions

Are the conclusions adequately supported by the data shown? Choose an item.

Reporting Standards

Does the manuscript adhere to the journal's guidelines on minimum standards of reporting? Choose an item.

Choose an item.

Statistics

Are you able to assess all statistics in the manuscript, including the appropriateness of statistical tests used? Choose an item.

Quality of Written English

Please indicate the quality of language in the manuscript: Choose an item.

Declaration of Competing Interests

Please complete a declaration of competing interests, considering the following questions:

- Have you in the past five years received reimbursements, fees, funding, or salary from an organisation that may in any way gain or lose financially from the publication of this manuscript, either now or in the future?
- Do you hold any stocks or shares in an organisation that may in any way gain or lose financially from the publication of this manuscript, either now or in the future?
- Do you hold or are you currently applying for any patents relating to the content of the manuscript?
- Have you received reimbursements, fees, funding, or salary from an organization that holds or has applied for patents relating to the content of the manuscript?
- Do you have any other financial competing interests?
- Do you have any non-financial competing interests in relation to this paper?

If you can answer no to all of the above, write 'I declare that I have no competing interests' below. If your reply is yes to any, please give details below.

NA

I agree to the open peer review policy of the journal. I understand that my name will be included on my report to the authors and, if the manuscript is accepted for publication, my named report including any attachments I upload will be posted on the website along with the authors' responses. I agree for my report to be made available under an Open Access Creative Commons CC-BY license (http://creativecommons.org/licenses/by/4.0/). I understand that any comments which I do not wish to be included in my named report can be included as confidential comments to the editors, which will not be published.

Choose an item.

To further support our reviewers, we have joined with Publons, where you can gain additional credit to further highlight your hard work (see: https://publons.com/journal/530/gigascience). On publication of this paper, your review will be automatically added to Publons, you can then choose whether or not to claim your Publons credit. I understand this statement.

Yes Choose an item.
