# Supplementary material for: Interpretable and accurate prediction models for metagenomics data
Source: Gigascience. 2020 Mar 9;9(3):giaa010. doi: 10.1093/gigascience/giaa010 (PMC7062144; doi:10.1093/gigascience/giaa010)
Supplement: giaa010_Reviewer_1_Report_Revision_1 — Kang Ning -- 9/16/2019 Reviewed [file giaa010_reviewer_1_report_revision_1.pdf]

**Reviewer Report**

**Title: Interpretable and accurate prediction models for metagenomics data**

**Version: Revision 1** **Date:** 9/16/2019

**Reviewer name: Kang Ning**

**Reviewer Comments to Author:**

This paper proposed a simple and interpretable disease classification prediction model, called predomics. It achieves the advantages that complex models do not have: the simplicity and interpretability of the model, while the overall performance on 54 datasets is better than the complex model (SOTA). The innovation is to infer the treatment effect and prognosis of patients through species abundance characteristics.
However, the problem with the article is:
In the method section, the importance of features can be calculated. However, how to select the features that will be calculated is not directly explained. Maybe the species abundance of biomarkers?
In the results section, there still remain unclear of calculating Bin and Ter ratio which will be compared with a prediction threshold. For example, how to optimize the prediction threshold.
In the evaluation and comparison of the predomics with other models, such as classification tasks, in addition to the accuracy rate, more evaluation indicators should also be introduced, such as precision, recall, F1-score, etc.
In the discussion section, a more thorough discussion is needed on the interpretable characteristics of the model. The recommended way is to combine the important features given by the predomics model with the patient's gut microbiome data to find potential biomarkers and then make a further explanation.

**Methods**

Are the methods appropriate to the aims of the study, are they well described, and are necessary controls included? Choose an item.

**Conclusions**

Are the conclusions adequately supported by the data shown? Choose an item.

**Reporting Standards**

Does the manuscript adhere to the journal's guidelines on minimum standards of reporting? Choose an item.

Choose an item.

**Statistics**

Are you able to assess all statistics in the manuscript, including the appropriateness of statistical tests used? Choose an item.

**Quality of Written English**

Please indicate the quality of language in the manuscript: Choose an item.

**Declaration of Competing Interests**

Please complete a declaration of competing interests, considering the following questions:

- Have you in the past five years received reimbursements, fees, funding, or salary from an organisation that may in any way gain or lose financially from the publication of this manuscript, either now or in the future?
- Do you hold any stocks or shares in an organisation that may in any way gain or lose financially from the publication of this manuscript, either now or in the future?
- Do you hold or are you currently applying for any patents relating to the content of the manuscript?
- Have you received reimbursements, fees, funding, or salary from an organization that holds or has applied for patents relating to the content of the manuscript?
- Do you have any other financial competing interests?
- Do you have any non-financial competing interests in relation to this paper?

If you can answer no to all of the above, write 'I declare that I have no competing interests' below. If your reply is yes to any, please give details below.

I declare that I have no competing interests

I agree to the open peer review policy of the journal. I understand that my name will be included on my report to the authors and, if the manuscript is accepted for publication, my named report including any attachments I upload will be posted on the website along with the authors' responses. I agree for my report to be made available under an Open Access Creative Commons CC-BY license (http://creativecommons.org/licenses/by/4.0/). I understand that any comments which I do not wish to be included in my named report can be included as confidential comments to the editors, which will not be published.

Choose an item.

To further support our reviewers, we have joined with Publons, where you can gain additional credit to further highlight your hard work (see: https://publons.com/journal/530/gigascience). On publication of this paper, your review will be automatically added to Publons, you can then choose whether or not to claim your Publons credit. I understand this statement.

Yes Choose an item.
