# Supplementary material for: Interpretable and accurate prediction models for metagenomics data
Source: Gigascience. 2020 Mar 9;9(3):giaa010. doi: 10.1093/gigascience/giaa010 (PMC7062144; doi:10.1093/gigascience/giaa010)
Supplement: giaa010_Reviewer_1_Report_Revision_2 — Kang Ning -- 12/15/2019 Reviewed [file giaa010_reviewer_1_report_revision_2.pdf]

Reviewer Report

Title: Interpretable and accurate prediction models for metagenomics data

Version: Revision 2   **Date:** 12/15/2019

Reviewer name: Kang Ning

Reviewer Comments to Author:

1. "Consequently, most models tend to overfit the training data and result in predictions arising from random sampling fluctuations." Please cite the literatures to support your view.
2. The paper should be carefully checked, such as lacing full stop at the end of the paragraph.
Also found this phenome in Figure legends.
3. The structure of the article is inconsistent, please format it.
4. The unformatted font.
5. The conclusion is abstract and unclear.
6. Mixed use of full names and abbreviations of Journal in Reference, what's more some have Vol, and some don't, such as:
"
5.Walsh, C.J., et al., Beneficial modulation of the gut microbiota. FEBS Letters, 2014.
588(22): p. 4120-4130.
6. Ley, R.E., et al., Obesity alters gut microbial ecology. Proceedings of the National
Academy of Sciences, 2005. 102(31): p. 11070.
7. Qin, N., et al., Alterations of the human gut microbiome in liver cirrhosis. Nature,
2014. 513: p. 59.
8. Wen, L., et al., Innate immunity and intestinal microbiota in the development of Type1 diabetes.
Nature, 2008. 455(7216): p. 1109-1113.
9. Qin, J., et al., A metagenome-wide association study of gut microbiota in type 2
diabetes. Nature, 2012. 490: p. 55."

Methods

Are the methods appropriate to the aims of the study, are they well described, and are necessary controls included? Choose an item.

Conclusions

Are the conclusions adequately supported by the data shown? Choose an item.

Reporting Standards

Does the manuscript adhere to the journal's guidelines on minimum standards of reporting? Choose an item.

Choose an item.

Statistics

Are you able to assess all statistics in the manuscript, including the appropriateness of statistical tests used? Choose an item.

Quality of Written English

Please indicate the quality of language in the manuscript: Choose an item.

Declaration of Competing Interests

Please complete a declaration of competing interests, considering the following questions:

- Have you in the past five years received reimbursements, fees, funding, or salary from an organisation that may in any way gain or lose financially from the publication of this manuscript, either now or in the future?
- Do you hold any stocks or shares in an organisation that may in any way gain or lose financially from the publication of this manuscript, either now or in the future?
- Do you hold or are you currently applying for any patents relating to the content of the manuscript?
- Have you received reimbursements, fees, funding, or salary from an organization that holds or has applied for patents relating to the content of the manuscript?
- Do you have any other financial competing interests?
- Do you have any non-financial competing interests in relation to this paper?

If you can answer no to all of the above, write 'I declare that I have no competing interests' below. If your reply is yes to any, please give details below.

I declare that I have no competing interests

I agree to the open peer review policy of the journal. I understand that my name will be included on my report to the authors and, if the manuscript is accepted for publication, my named report including any attachments I upload will be posted on the website along with the authors' responses. I agree for my report to be made available under an Open Access Creative Commons CC-BY license (http://creativecommons.org/licenses/by/4.0/). I understand that any comments which I do not wish to be included in my named report can be included as confidential comments to the editors, which will not be published.

Choose an item.

To further support our reviewers, we have joined with Publons, where you can gain additional credit to further highlight your hard work (see: https://publons.com/journal/530/gigascience). On publication of this paper, your review will be automatically added to Publons, you can then choose whether or not to claim your Publons credit. I understand this statement.

Yes Choose an item.
