# Supplementary material for: Interpretable and accurate prediction models for metagenomics data
Source: Gigascience. 2020 Mar 9;9(3):giaa010. doi: 10.1093/gigascience/giaa010 (PMC7062144; doi:10.1093/gigascience/giaa010)
Supplement: giaa010_Reviewer_2_Report_Original_Submission — Bonnie Hurwitz -- 8/2/2019 Reviewed [file giaa010_reviewer_2_report_original_submission.pdf]

Reviewer Report

Title: Interpretable and accurate prediction models for metagenomics data

Version: Original Submission **Date:** 8/2/2019

Reviewer name: Bonnie Hurwitz

Reviewer Comments to Author:

The authors present a well-written manuscript that addresses the potential for explainable machine learning approaches to diagnose disease using metagenomics data. Specifically, the authors present a machine-learning approach that they call "predomics" to search for predictive signatures that are interpretable. This is an important step forward in converting raw metagenomic data into actionable predictive analyses that can inform clinical decision-making. To validate their method, they compare and contrast SOTA algorithms to BTR models using 109 datasets taken from a curated metagenomic dataset (Pasolli et al. 2017) comprised of studies that used shotgun metagenomic sequencing and were analyzed using a consistent pipeline. Datasets included in this analysis are a subset of the curated dataset including gut microbiome studies with varied disease states such as liver cirrhosis, IBD, type 2 diabetes, and obesity-related phenotypes. I commend the authors on their use of a curated dataset (Pasolli et al. 2017), as it helps to align this study with other studies. Further, the consistent annotation makes the data comparable across studies in this analysis. Finally, I think their experiments show that BTR models offer a simplified interpretation compared to the more complex models, which is needed for translating clinical data. The authors are thorough in their analysis and produce compelling results, however, I have a few remaining questions and note a few typos they should correct in the final proof.

1. Abstract has two words misspelled, should say "state-of-the-art" and "distill"
2. BTR is introduced in the Methods section on page 4, but the acronym isn't defined until page 6.
3. The code is not currently available here: https://git.integromics.fr/published/predomics
4. The statement that "The Virus phylum was not previously identified but is more prevalent and abundant in the liver cirrhosis group, which may reflect the oral microbiome signature and/or reflect an increased incidence of viral infections together with opportunistic pathogens", needs to be more carefully considered. First, the original paper that the data are from did not compare against viral reference genomes, only bacteria and archaea (see supplemental Table 2 in Qin et. al). Thus, no viruses were detected simply because they were not used in the analysis. Second, the curated dataset used in this paper from Pasolli et al. uses MetaPhlAn to assign taxonomy. MetaPhlAn uses a genome size correction in the algorithm when assigning organismal abundance, rather than read abundance alone. As a result, small genomes (from viruses and plasmids) can be overamplified in the resulting counts. Meaning that a small number of viral reads can have a big effect on the overall abundance of viruses in the sample, and may have an impact on the ratio of organisms which highly variable genome sizes and thus affect this conclusion. Can the authors provide further clarification of the impact of this algorithmic choice in calculating abundance and their resulting analyses?
5. Recently, shallow whole genome shotgun sequencing has been proposed as a more cost-effective approach to provide more accurate taxonomic information (compared to 16S rRNA) for clinical use. Can

the authors comment on how shallow WGS sequencing might affect their algorithm and interpretation of results? Further, sequencing experiments have a varied depth of coverage, and therefore organisms/functional traits that are rarer might not be detected with shallow sequencing. Because the authors are focused on clinical interpretations and guidance for clinicians and patients, a discussion of these points is important and relevant in this manuscript.
6. Have the authors considered the runtime for their method to produce clinically relevant data and results? The method requires extensive annotation including genes, pathways, taxonomic annotation, and then curation and association analysis based on co-occurrence networks and functional analysis (e.g whether or not organisms are butyrate producers). Would the algorithm be better suited for biomarker discovery and validation, where biomarkers are then used in the microbiology lab via simple qPCR-based tests? Logistically, this approach might be best suited for less-time sensitive tasks. If so, the authors should note this.
7. Please convert all instances of "16S" to "16S rRNA", as in "16S surveys" on page 11
8. The authors use rich WGS datasets that have multiple features that can be used in training the algorithm such as taxonomic abundance from all domains of life (bacteria, archaea, fungi) and viruses, metabolic profiles, and curated patient metadata. But, suggest that species-level abundances are a key feature of their approach in defining ecosystem-level interactions for prediction. Can this approach be used with 16S rRNA data alone? Have the authors tested this? Given issues with human contamination in the vast number of clinical samples, the utility of this approach on 16S rRNA datasets would be of interest to the community and readers.
Overall, really nicely written paper, and careful analyses that is worthy of publication.

Methods

Are the methods appropriate to the aims of the study, are they well described, and are necessary controls included? Choose an item.

Conclusions

Are the conclusions adequately supported by the data shown? Choose an item.

Reporting Standards

Does the manuscript adhere to the journal's guidelines on minimum standards of reporting? Choose an item.

Choose an item.

Statistics

Are you able to assess all statistics in the manuscript, including the appropriateness of statistical tests used? Choose an item.

Quality of Written English

Please indicate the quality of language in the manuscript: Choose an item.

Declaration of Competing Interests

Please complete a declaration of competing interests, considering the following questions:

- Have you in the past five years received reimbursements, fees, funding, or salary from an organisation that may in any way gain or lose financially from the publication of this manuscript, either now or in the future?
- Do you hold any stocks or shares in an organisation that may in any way gain or lose financially from the publication of this manuscript, either now or in the future?
- Do you hold or are you currently applying for any patents relating to the content of the manuscript?
- Have you received reimbursements, fees, funding, or salary from an organization that holds or has applied for patents relating to the content of the manuscript?
- Do you have any other financial competing interests?
- Do you have any non-financial competing interests in relation to this paper?

If you can answer no to all of the above, write 'I declare that I have no competing interests' below. If your reply is yes to any, please give details below.

I declare that I have no competing interests

I agree to the open peer review policy of the journal. I understand that my name will be included on my report to the authors and, if the manuscript is accepted for publication, my named report including any attachments I upload will be posted on the website along with the authors' responses. I agree for my report to be made available under an Open Access Creative Commons CC-BY license (http://creativecommons.org/licenses/by/4.0/). I understand that any comments which I do not wish to be included in my named report can be included as confidential comments to the editors, which will not be published.

Choose an item.

To further support our reviewers, we have joined with Publons, where you can gain additional credit to further highlight your hard work (see: https://publons.com/journal/530/gigascience). On publication of this paper, your review will be automatically added to Publons, you can then choose whether or not to claim your Publons credit. I understand this statement.

Yes Choose an item.
